# Understanding the Modes of Action of β‑Ketoiminato Iridium(III) Complexes in Cancer Cells

**DOI:** 10.1021/acs.inorgchem.5c02026

**Published:** 2025-08-22

**Authors:** Tameryn Stringer, Büşra Yildirim, Baris Sergi, Benjamin J. Hofmann, Yi-Hsuan Lee, Ceyda Acilan, Rianne M. Lord

**Affiliations:** † Department of Chemistry, 2707 University of Warwick, Coventry CV4 7AL, United Kingdom; ‡ School of Chemistry, Pharmacy and Pharmacology, 6106 University of East Anglia, Norwich, Norfolk NR4 7TJ, United Kingdom; § School of Science, The University of Waikato, Hamilton 3210, New Zealand; ∥ Graduate School of Health Sciences, 52979 Koç University, Istanbul 34450, Turkey; ⊥ Koç University Translational Research Center, KUTTAM, Istanbul 34450, Turkey; # School of Medicine, Koç University, Sariyer, Istanbul 34450, Turkey

## Abstract

Four new charged iridium­(III) 1,2,3,4,5-pentamethylcyclopentadienyl (Cp*) complexes, **1–4**, of the type [Cp*Ir­(**L1–4**)­(PTA)]­(PF_6_) (where **L1–4** = functionalized β-ketoiminate ligands and PTA = 1,3,5-triaza-7-phosphaadamantane), have been successfully synthesized and characterized. Single crystal X-ray crystallographic data have been obtained for all compounds and confirm a typical *pseudo*-octahedral half-sandwich geometry. Cytotoxicity values have been determined against a range of cancerous and noncancerous cell lines and highlight high cytotoxicity and selectivity toward breast cancers. Among these compounds, the unfunctionalized β-ketoiminate Ir­(III) complex (**1**) emerged as the most promising candidate, demonstrating activity that was comparable to or exceeded that of cisplatin, especially after 24 h against the triple-negative MDA-MB-231 cell line. Morphological and molecular analyses confirmed that **1** triggers apoptotic cell death, involving caspase activation and PARP cleavage, which is consistent with its DNA-damaging characteristics, highlighting the future anticancer potential of compound **1**.

## Graphical abstract

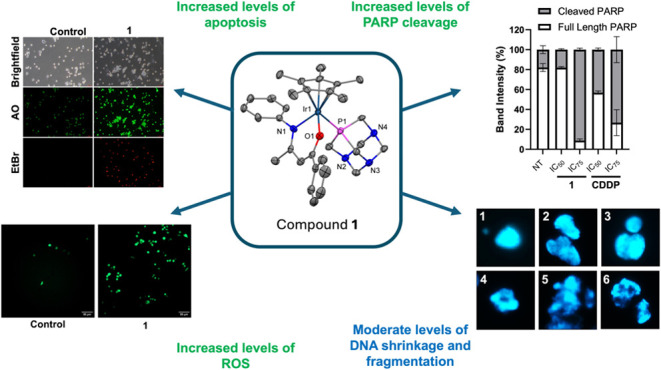

## Introduction

Platinum-based anticancer drugs such as cisplatin have a range of complications associated with their administration, including negative and toxic side effects and intrinsic/acquired resistance.^1^ These drugs are also limited to treating specific types of cancers.^2^ Medicinal inorganic chemists have since focused on developing molecules which can overcome these constraints, creating new drugs with high but targeting cytotoxicity, different intracellular modes of action and the ability to treat platinum-resistant tumors; all in the hope to reduce the devastating patient side-effects associated with current clinical metallodrugs.^3^

Metallodrugs containing platinum-group metals (PGMs), e.g., ruthenium, osmium, rhodium, and iridium, have shown promising biological properties in recent years.^4–8^ Organometallic half-sandwich compounds, specifically those based on Ru­(II), have generated significant interest for their bioapplications,^9^ including RM175 (Figure 1A) a ethylenediamine compound by Sadler and co-workers,^10^ and both RAPTA-T and RAPTA-C 1,3,5-triaza-7-phosphoadamantane (PTA) compounds (Figure 1B,C) by Dyson and co-workers,^11^ which all show impressive activity.

**Figure 1:**
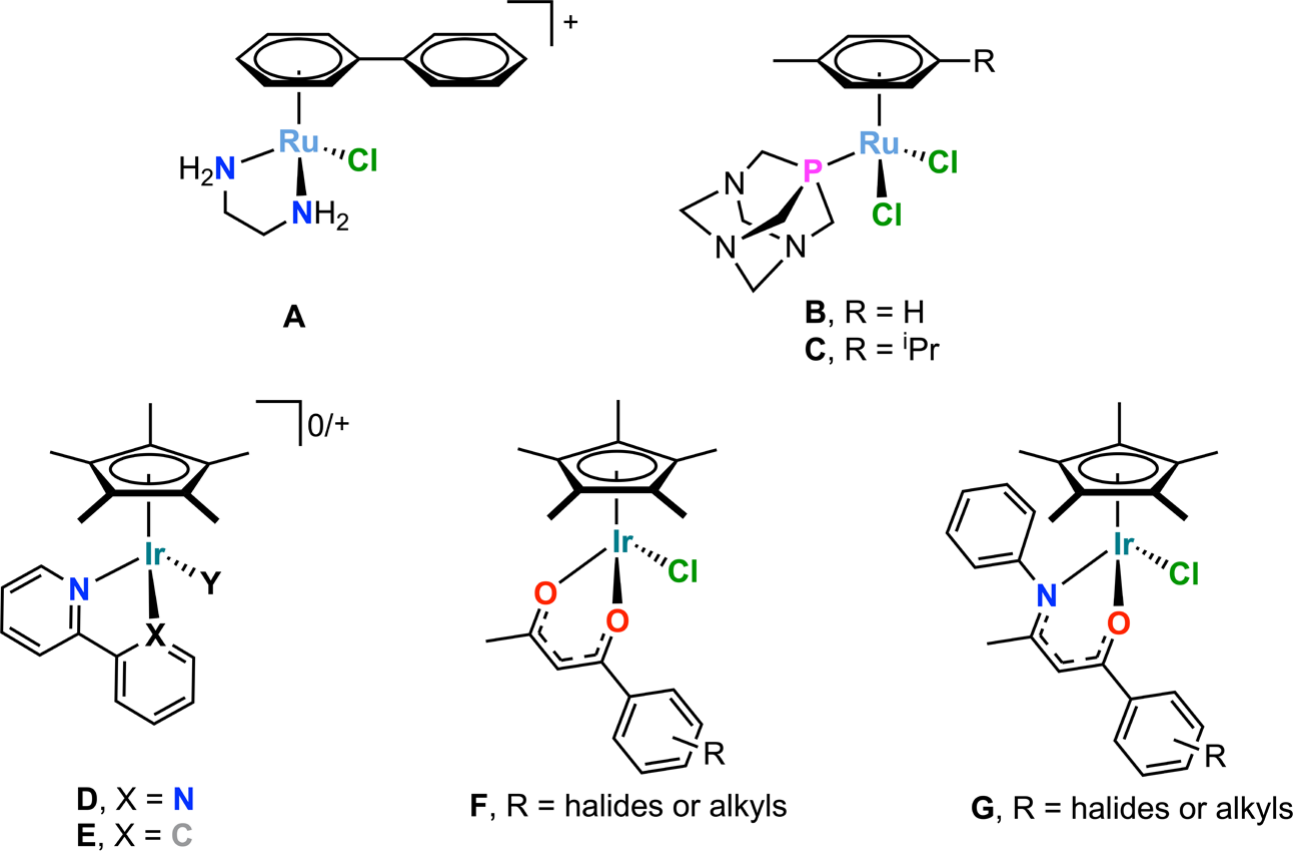
A range of bioactive half-sandwich PGM compounds.

Other PGM compounds, for example, those based on un/functionalized pentamethylcyclopentadienyl (Cp*)-Ir­(III) and Rh­(III) motifs, have been readily modified to improve lipophilicity and subsequently improve activity.^8,12,13^ Both neutral and cationic Cp*-Ir­(III) compounds containing a variety of bidentate ligands, for example, coordination through *N*,*N* and *N*,*C* ligands (e.g., Figure 1D,E), are reported to induce apoptosis,^14^ and changing the chelating *N*,*N*-ligand for a *N*,*C*-ligand led to a significant improvement in bioactivity.^15^ PGM compounds with β-diketone (*O*,*O*) ligands have been reported by us and others (e.g., Figure 1F); however, the *O*,*O*-ligands yielded poorly cytotoxic compounds when compared to previously reported *N*,*N* and *N*,*C* compounds.^16,17^

To allow comparisons of ligand effects, we also reported both arene-Ru­(II) and Cp*-Ir­(III) compounds containing *N*,*O* β-ketoiminate ligands,^17,18^ and showed their cytotoxicity was significantly higher than the corresponding *O*,*O* β-diketonato compounds. Notably, compounds of the type [Cp*Ir­(*N*,*O*)­Cl] (Figure 1G) were shown to have moderate to high cytotoxicity against various cancer cell lines, with increased activity against colorectal cancer, and higher cancer cell line selectivity than cisplatin.^19^

PGM compounds, which are modified with PTA ligands, have consistently shown improvements in solubility while either maintaining or improving activity.^20^ This study focuses on understanding the biological effects of incorporating PTA ligands into our previously reported β-ketoiminato Cp*-Ir­(III) chlorido compounds. Comparisons have been made with a small range of compounds that exhibited low-moderate (R = H, 2′,4′-diCl) or moderate-high (R = 4′-Br, 4′-OEt) cytotoxicity.^19^ Herein, we report their preparation, structural characterization and evaluation of their biological *in vitro* modes of action using DNA, apoptosis, caspase, and microscopy assays.

## Results and Discussion

### Synthesis and Characterization

The β-ketoiminate ligands **L1–4** were synthesized and isolated according to our previously reported methods,^17–19^ and used in the preparation of four new charged Cp*-Ir­(III) PTA compounds **1–4** (Scheme 1). All new compounds were isolated in moderate yields (46–69%) and characterized by ^1^H, ^13^C­{^1^H} and ^31^P­{^1^H} NMR spectroscopy (Figures S1–S12), elemental analysis, high-resolution mass spectrometry (Figures S33–S36), and single-crystal X-ray diffraction (scXRD). ^1^H NMR spectra show the characteristic resonances for the methine singlet between 6.0–5.4 ppm, the disappearance of the free ligand NH at *ca*. 13 ppm and new PTA resonances between 4.4–4.7 ppm. ^13^C­{^1^H} NMR spectra show successful formation with the PTA ligand appearing as doublets at *ca*. 73 and 50 ppm due to the ^3^ *J*(^13^C–^31^P) and ^1^ *J*(^13^C–^31^P) coupling, respectively. While the ^31^P­{^1^H} NMR spectra show the two phosphorus environments for the PTA ligand, a singlet at *ca*. −61 ppm, and the PF_6_ anion, a septet at *ca*. −144 ppm.

**Scheme 1:**
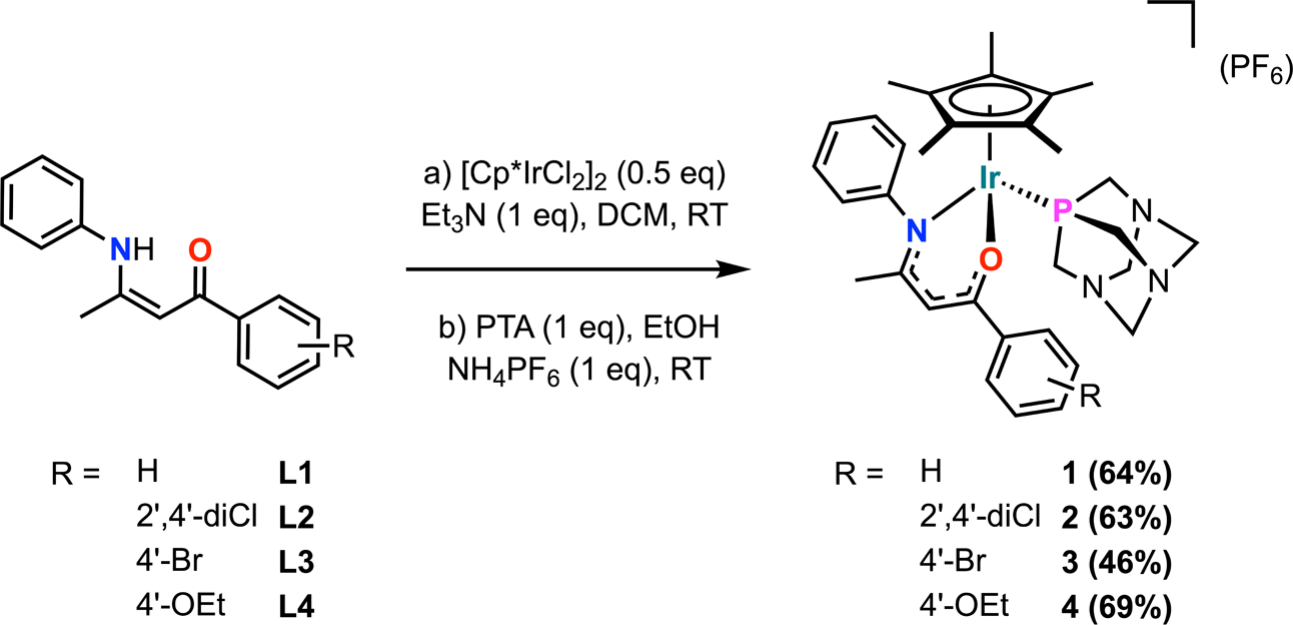
Synthetic Procedures for Compounds **1-4** from Ligands **L1-4**

### Stability Studies

^1^H and ^31^P NMR spectra were used to support the stability studies, and compounds **1–4** were dissolved in a mixture of 90% DMSO-*d*_6_ and 10% D_2_O and NMR spectra collected over 96 h. Representative NMR spectra at the start, and then after 12, 24, and 96 h are presented in the Supporting Information (Figures S13–S20), and show no hydrolysis or decomposition of the compounds over this period, which is in line with previously reported β-diketonato Cp*-Ir­(III) PTA complexes by Pettinari et al.^21^

Pettinari et al. showed that in the presence of elevated chloride concentrations, similar to those found in blood serum or cell culture media (DMEM, RPMI), the ligands dissociate, and the respective Cp*-Ir­(III) chlorido complexes are formed. To test whether this is the case for complexes **1–4**, stabilities were assessed over 96 h in 70% DMSO-*d*_6_ and 30% D_2_O mixtures spiked with 0.1 M NaCl (Figures S21–S28). In contrast to the discussed β-diketonato complexes, no decomposition was observed with complexes **1–4**, demonstrating the stronger donor properties of the β-ketoiminate ligands and higher stability of the complexes. This was also confirmed by analyzing the HR-MS spectra after the compounds (in the same conditions) were incubated for 96 h. The spectra show no DMSO adducts and only *m*/*z* values for [M-PF_6_] and [M-PF_6_–PTA] (Figures S37–S40) were observed.

Due to low solubility, the percentage of water could not be increased further in the NMR studies; therefore, ultraviolet/visible (UV/vis) stability studies were conducted using compounds **1–4** in fully supplemented phenol-red free media over 96 h at 37 °C. Unlike the NMR experiments, the UV/vis spectra (Figures S29–S32) show that all compounds hydrolyze in high concentrations of water and the complex environment of the media. The rate of decomposition is dependent on the substitution pattern where compound **1** fully decomposes after *ca.* 90 h, the compounds (**2** and **4**) are significantly less stable and no changes in the spectra are observed after 64 and 72 h, respectively, and the highest stability is observed for compound **3** (>96 h).

### Single Crystal X-ray Diffraction (scXRD)

Yellow-orange single crystals of complexes **1–4** suitable for scXRD analysis were grown by slow evaporation of a concentrated solution of acetone at room temperature (CSD: 239282–2392865). The structural solutions were performed in monoclinic (**1–3**; *P*2_1_/*c* and *P*2_1_/*n*) or triclinic (**4**; *P*1̅) space groups, with either one or three molecules in the unit cell. The molecular structures are shown in Figure 2, and all bond lengths (Table 1) and bond angles (Table S2) are in the range of the previously reported Cp*-Ir­(III) complexes.^19^ Other crystallographic data are presented in Table S1 of the Supporting Information.

**Figure 2:**
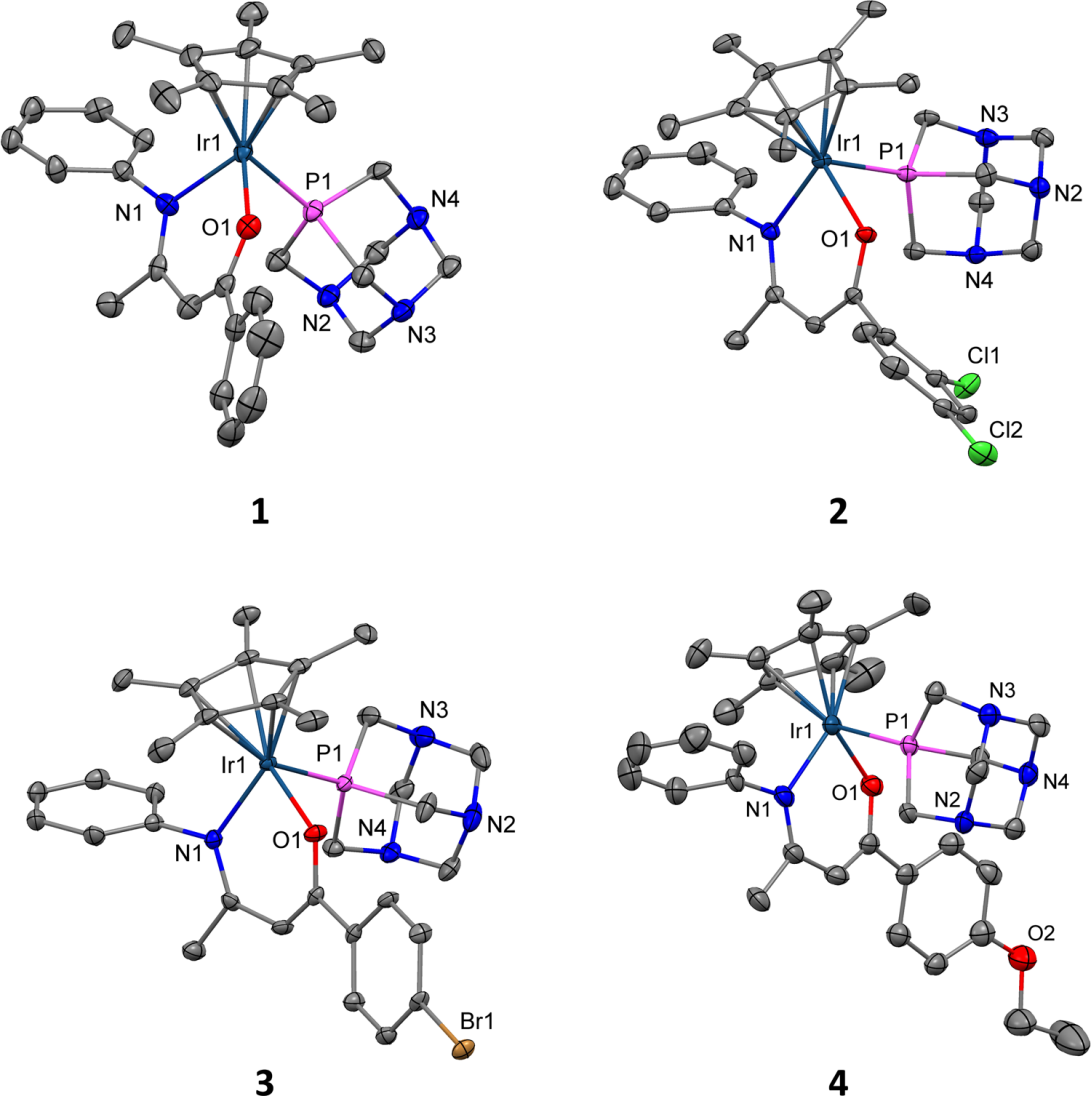
Molecular structures of complexes **1–4**. Hydrogen atoms and hexafluorophosphate anions are omitted for clarity, and displacement ellipsoids are placed at the 50% probability level.

**Table 1:** Bond Lengths (Å) of Complexes **1-4** with s.u.s in Parentheses

bond lengths (Å)	**1**	**2**	**3**	**4**
**Ir1–P1**	2.3164(12)	2.2964(7)	2.3190(9)	2.3022(16)/2.3049(17)/2.3212(19)
**Ir1–O1**	2.115(4)	2.1014(18)	2.088(2)	2.093(5)/2.097(4)/2.080(5)
**Ir1–N1**	2.108(4)	2.122(2)	2.101(3)	2.108(5)/2.112(6)/2.106(5)
**C1–O1**	1.294(6)	1.296(3)	1.292(4)	1.313(7)/1.300(8)/1.290(8)
**C3–N1**	1.324(7)	1.317(3)	1.326(4)	1.322(9)/1.300(9)/1.339(9)
Ir-Cp* centroid	1.832	1.843	1.842	1.832/1.834/1.844

### Chemosensitivity Studies

Chemosensitivity studies were conducted using human cell lines: breast adenocarcinomas MDA-MB-231 and MCF-7, pancreatic carcinoma MIA PaCa-2 and a noncancerous retinal epithelial cell line ARPE-19, after being incubated for 96 h with compounds **1–4**, cisplatin (CDDP) or carboplatin (CARB) (Table 2 and Figure 3). The ligands and the Cp*-Ir­(III) dimer precursor have already been screened and show no or low cytotoxicity.^17,19,22^ Generally, **1–4** exhibit moderate to high cytotoxicity against MDA-MB-231 and MIA PaCa-2, with IC_50_ values ranging from 4.1 ± 0.5 μM (MDA-MB-231, **4**) to 14.9 ± 0.7 μM (MIA PaCa-2, **2**). However, the cytotoxicity is significantly reduced against MCF-7, with IC_50_ values ranging from 32.4 ± 0.3 μM (**3**) to 48 ± 3 μM (**1**). Compounds **1–4** are less active than CDDP against all cell lines tested (Figure S41); however, they have increased selectivity when compared to CARB, with IC_50_ values between 2.7–4.4x higher against MIA PaCa-2 and 2.5–3.9x higher against MDA-MB-231 (Figure S42).

**Figure 3:**
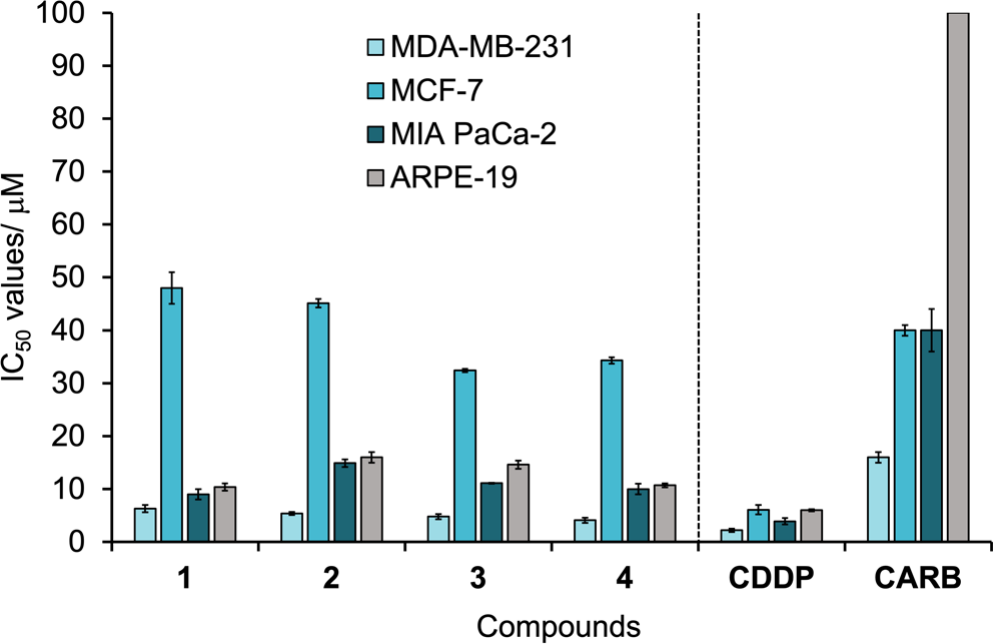
IC_50_ values (μM) ± SD for compounds **1–4**, cisplatin (CDDP) and carboplatin (CARB): Cancer cell lines MDA-MB-231, MCF-7, MIA PaCa-2, and noncancerous cell line ARPE-19 were exposed to the compounds for 96 h (*n* = 9).

**Table 2:** IC_50_ Values (μM) ± SD for Compounds **1-4**, Cisplatin (CDDP) and Carboplatin (CARB) When Tested against MDA-MB-231, MCF-7, MIA PaCa-2 and ARPE-19 Cells for 96 h (or 24 h) (*n* = 9)^a^

	96 h				24 h
complex	MDA-MB-231	MCF-7	MIA PaCa-2	ARPE-19	MDA-MB-231
**1**	6.3 ± 0.7 (1.7)	48 ± 3 (0.2)	9 ± 1 (1.2)	10.4 ± 0.7	18 ± 1
**2**	5.4 ± 0.3 (3.0)	45.1 ± 0.8 (0.4)	14.9 ± 0.7 (1.1)	16 ± 1	21 ± 1
**3**	4.8 ± 0.5 (3.0)	32.4 ± 0.3 (0.5)	11.2 ± 0.1 (1.3)	14.6 ± 0.8	22 ± 1
**4**	4.1 ± 0.5 (2.6)	34.3 ± 0.6 (0.3)	10 ± 1 (1.1)	10.7 ± 0.4	36 ± 1
**CDDP**	2.2 ± 0.3 (2.7)	6.1 ± 0.9 (1.0)	3.9 ± 0.6 (1.5)	6.0 ± 0.2	>100
**CARB**	16 ± 1 (>6.3)	40 ± 1 (>2.5)	40 ± 4 (>2.5)	>100	>100

a Selectivity index (SI) values are shown in parentheses.

The cytotoxicity of the compounds against the cancer cell lines was also compared to the noncancerous retinal epithelial cell line, ARPE-19, to determine the possibility of cancer cell selectivity. The cytotoxic values are generally high for all compounds, meaning low selectivity is observed, e.g., SI = 0.2–0.5 or 1.1–1.3 against MCF-7 and MIA PaCa-2, respectively (Table 2 parentheses). Slight improvements in selectivity were observed against MDA-MB-231, with SI = 1.7–3.3.

The compounds generally performed consistently better against the triple-negative breast cell line, MDA-MB-231; therefore, the activity was also investigated after 24 h against this cell line (Table 2). Compound **1** exhibits slightly higher activity when compared to **2**-**3** and significantly higher activity when compared to compound **4**, CDDP and CARB. Therefore, in the following sections, an in-depth analysis of the modes of action was performed using compound **1** against MDA-MB-231, and in several cases, the activity is compared to the hormone-dependent breast cancer cell line, MCF-7 (results are shown in the Supporting Information).

### Generation of Reactive Oxygen Species (ROS)

ROS production in cells has been reported after treatment with Cp*-Ir­(III) complexes, by a range of pathways including NADH oxidation and induction of oxidative stress,^23–25^ indirectly due to the expression of specific proteins, whose levels are regulated by oxidative stress defense pathways,^26,27^ or via interaction with biomolecules such as glutathione (GSH), which is a crucial antioxidant in cells.^28^

To measure the degree of ROS produced in MDA-MB-231 cells, 2′,7′-dichlorodihydrofluorescein diacetate (H_2_DCFDA), a cell-permeable fluorescent dye, was used. H_2_DCFDA is deacetylated by esterases and consecutively oxidized by intracellular ROS into a fluorescent molecule, dichlorofluorescein (DCF).^29^ Due to its slightly enhanced activity in the MDA-MB-231 cell line after 24 h, the cell line was treated for 4 h with 100 μM of compound **1** (IC_50_ = 18 ± 1 μM after 24 h), and compared to a nontreated control. As shown in Figure 4, treatment with **1** leads to an increase in fluorescence, which suggests that ROS generation could be a plausible mode of action; however, further studies would be required to determine the pathways by which this ROS is generated.

**Figure 4:**
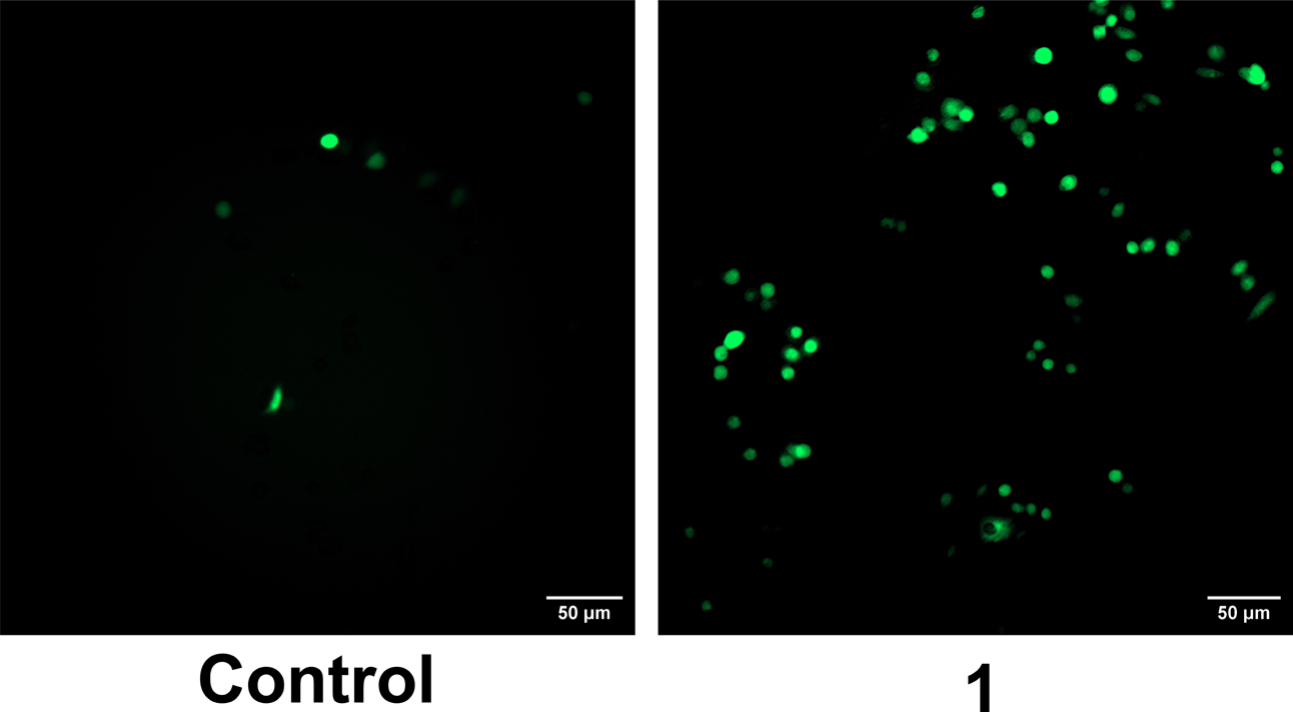
Reactive oxygen species (ROS) after MDA-MB-231 cells were treated with 100 μM of compound **1** for 4 h at 37 °C and compared to an untreated control. Scale bar = 50 μm.

### DNA Studies

The ability of compounds to intercalate, nick, or cleave DNA can be evaluated using gel electrophoresis with plasmid DNA. In this study, *in vitro* plasmid DNA assays were performed by incubating 200 ng of plasmid DNA with different concentrations (100–400 μM) of **1** or CDDP, which served as a positive control. The DNA fragments were then separated via agarose gel electrophoresis, and potential changes in DNA migration patterns were analyzed.

Treatment with **1** did not induce any apparent shift in plasmid DNA migration. While a slight reduction in the nicked circular form was observed, this difference was not statistically significant (*p* = 0.73), and the distribution of both supercoiled and nicked forms remained largely unchanged across concentrations. In contrast, CDDP treatment resulted in faster DNA migration, consistent with its well-known DNA coordination and our previously reported findings.^30^ These results suggest that **1** does not significantly interact with or damage DNA under cell-free conditions (Figure 5). These results are in line with other reported Ir­(III) complexes, where no DNA cleavage was observed even at very high concentrations of the complexes. DNA was found not to be the target for the complexes, but potentially targeted lysosomes instead.^24,25^ This experiment does not rule out DNA as a target of these complexes but does suggest that the complexes could act by more than one mechanism or have more than one biomolecular target.

**Figure 5:**
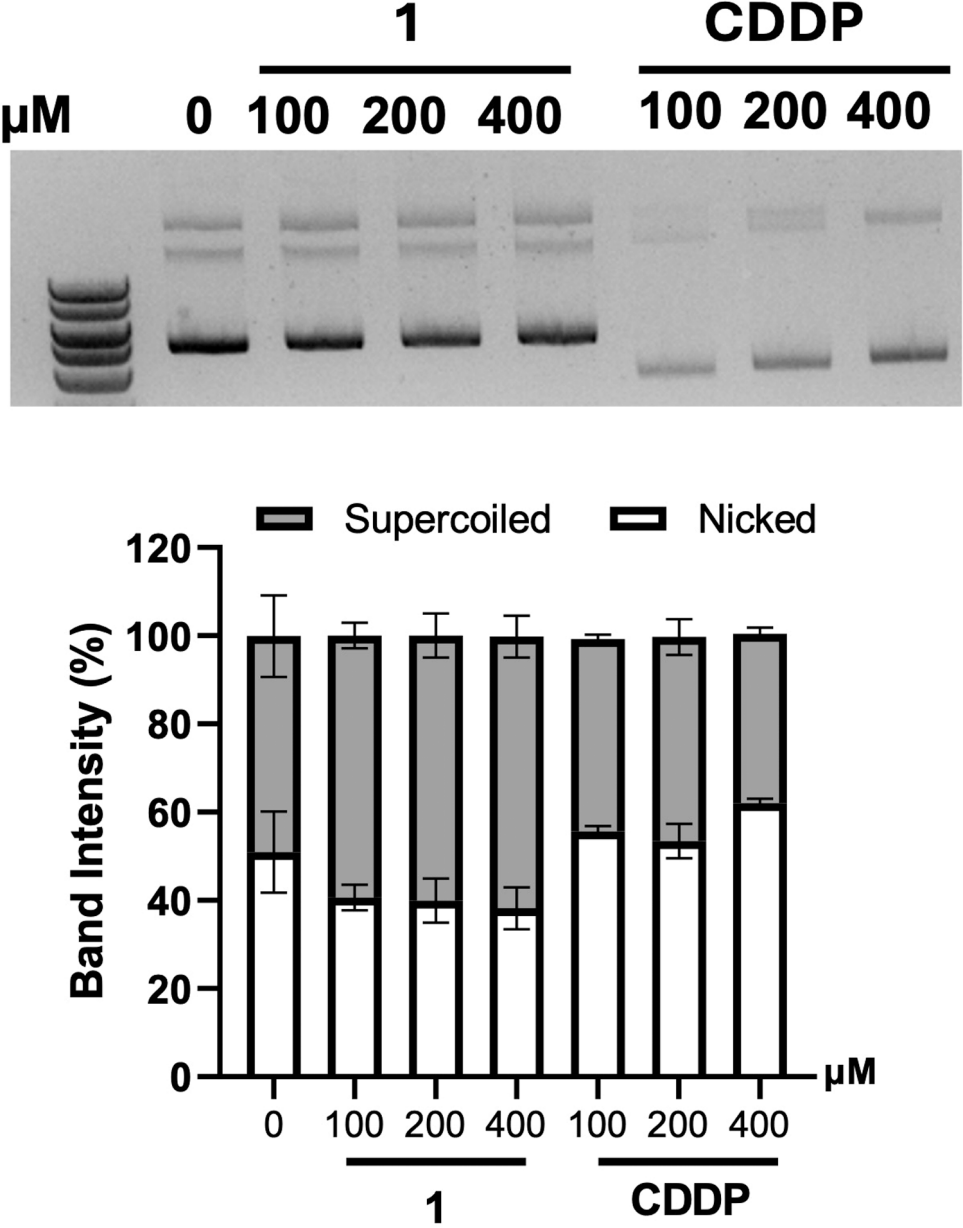
Plasmid DNA (200 ng) was incubated with varying concentrations (100, 200, and 400 μM) of compound **1** or CDDP for 24 h at room temperature. The DNA was then separated via agarose gel electrophoresis, and the intensity of supercoiled and nicked circular DNA bands was quantified and expressed as mean ± SEM (*n* = 4).

#### Evaluation of Cell Death through Morphological Analysis

To investigate how the drugs impacted cell viability, acridine orange (AO)/ethidium bromide (EtBr) staining was employed to identify the MDA-MB-231 cells' mode of death in response to compound **1** or CDDP, with AO marking early apoptotic cells in green and EtBr marking late apoptotic or necrotic cells in red. As shown in Figure 6, untreated controls displayed a faint AO signal and no EtBr staining, as expected. In contrast, treatment with **1** resulted in increased staining for both dyes, suggesting a predominant increase in apoptotic cell death. This effect was more pronounced than with CDDP, indicating its higher efficacy.

**Figure 6:**
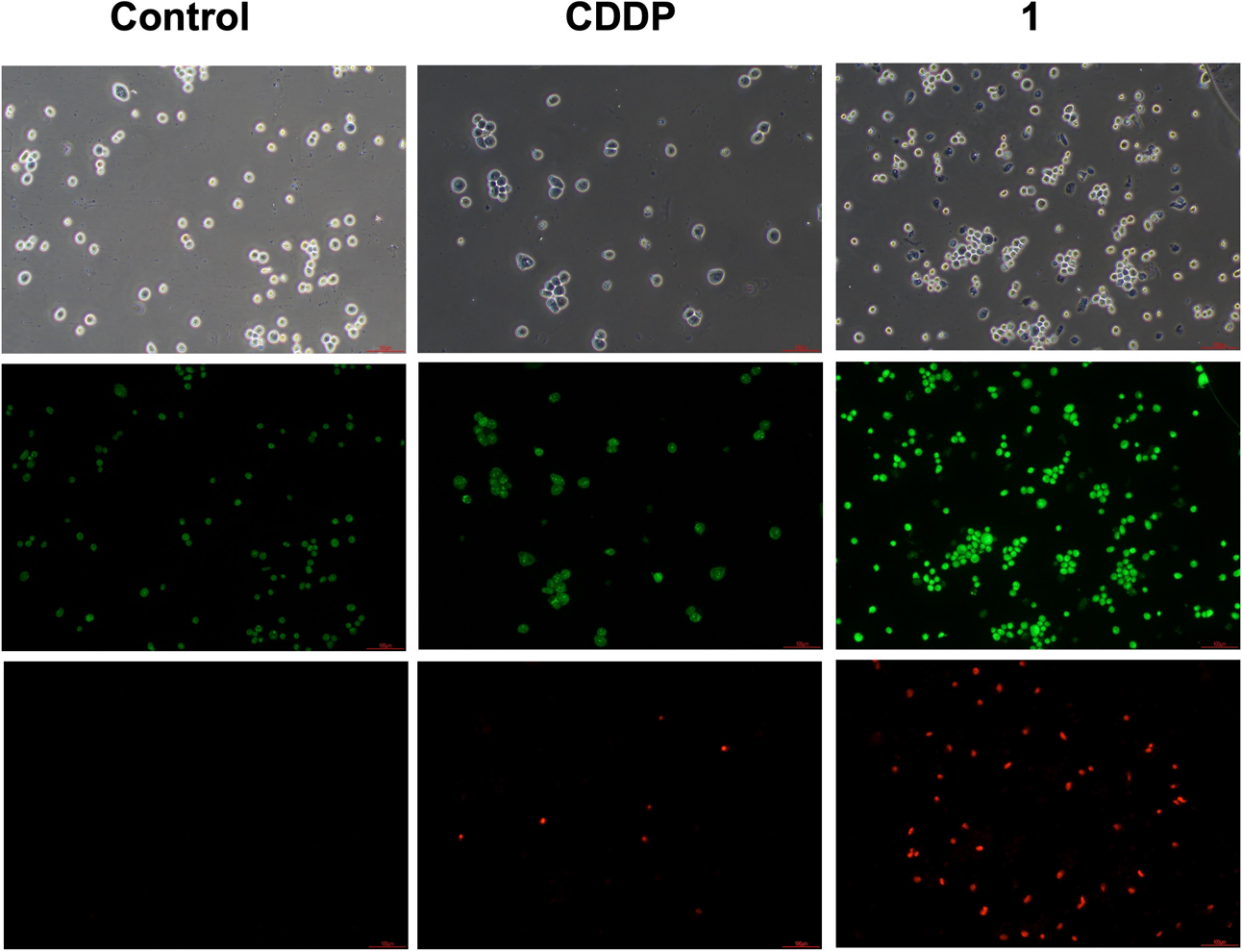
MDA-MB-231 cells after treatment with compound **1** or CDDP and evaluation of apoptosis using acridine orange/ethidium bromide (AO/EB) staining. The stained cells were observed under an Eclipse TS100 Nikon fluorescence microscope. Scale = 100 μm.

The potential of compound **1** to trigger apoptosis in breast cancer cells was then studied. Morphological analysis of cellular nuclei revealed typical apoptotic features like DNA fragmentation and condensation, confirming apoptosis as the primary form of cell death in MDA-MB-231 cells (Figure 7). High-magnification insets (1–6) illustrate representative nuclear morphologies captured in the assay. Condensed, hyperintense nuclei with reduced area (e.g., images 1, 2 and 3), irregular nuclear blebs with discontinuous chromatin (e.g., images 3 and 4), and fragmented chromatin consistent with apoptotic bodies (e.g., images 5 and 6). These results were consistent with the findings in MCF-7 cells (see Figure S43), suggesting that the results are not specific to a single cell line.

**Figure 7:**
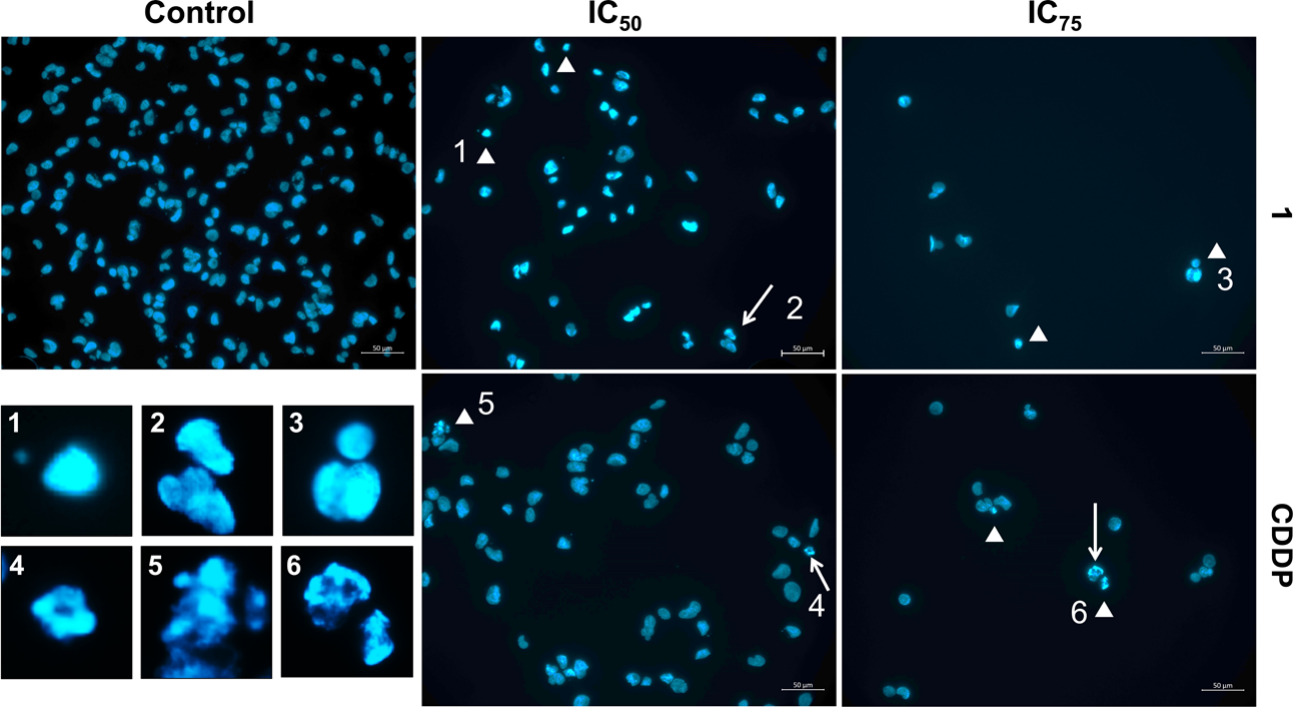
Microscopic visualization of nuclear morphology changes in MDA-MB-231 breast cancer cells after 48 h exposure to compound **1** or CDDP. Nuclear DNA was stained with DAPI, which intercalates into double-stranded DNA and fluoresces blue, allowing visualization of chromatin organization. Apoptotic alterations were observed, including nuclear shrinkage (arrowheads), chromatin condensation (appearing as smaller, hyperintense nuclei due to higher DNA packing), and DNA fragmentation forming apoptotic bodies (arrows). High-magnification insets (1–6) illustrate representative nuclear morphologies: condensed, intensely stained nuclei with reduced area (1–3), irregular nuclear blebs with discontinuous chromatin (3–4), and fragmented chromatin forming discrete apoptotic bodies (5–6). These morphological features confirm apoptosis as the predominant form of cell death. Images were acquired using a Leica DMI8 fluorescence microscope at 40× magnification. Scale bar = 50 μm.

#### Molecular Assessment of Cell Death

To assess whether apoptotic markers responded as anticipated, changes in Annexin V staining (Figure 8, top) and caspase activity in MDA-MB-231 cells (Figure 8, bottom) with compound **1** and CDDP were measured. Annexin V detects changes in membrane integrity, while caspases function as executioner enzymes, cleaving specific cellular proteins during apoptosis. Both assays showed a significant increase in apoptotic cells following treatment with **1**. Interestingly, while morphological assays revealed a higher proportion of apoptotic cells compared to CDDP, Annexin V staining and caspase activity were less pronounced, likely due to differences in the timing of the experiment’s end point (which was 48 h for morphological assays and 72 h for flow cytometric assays). This suggests distinct cellular response kinetics between **1** and CDDP, despite both treatments ultimately leading to apoptosis. Similar trends were observed in MCF-7 cells across both assays (Figure S44).

**Figure 8:**
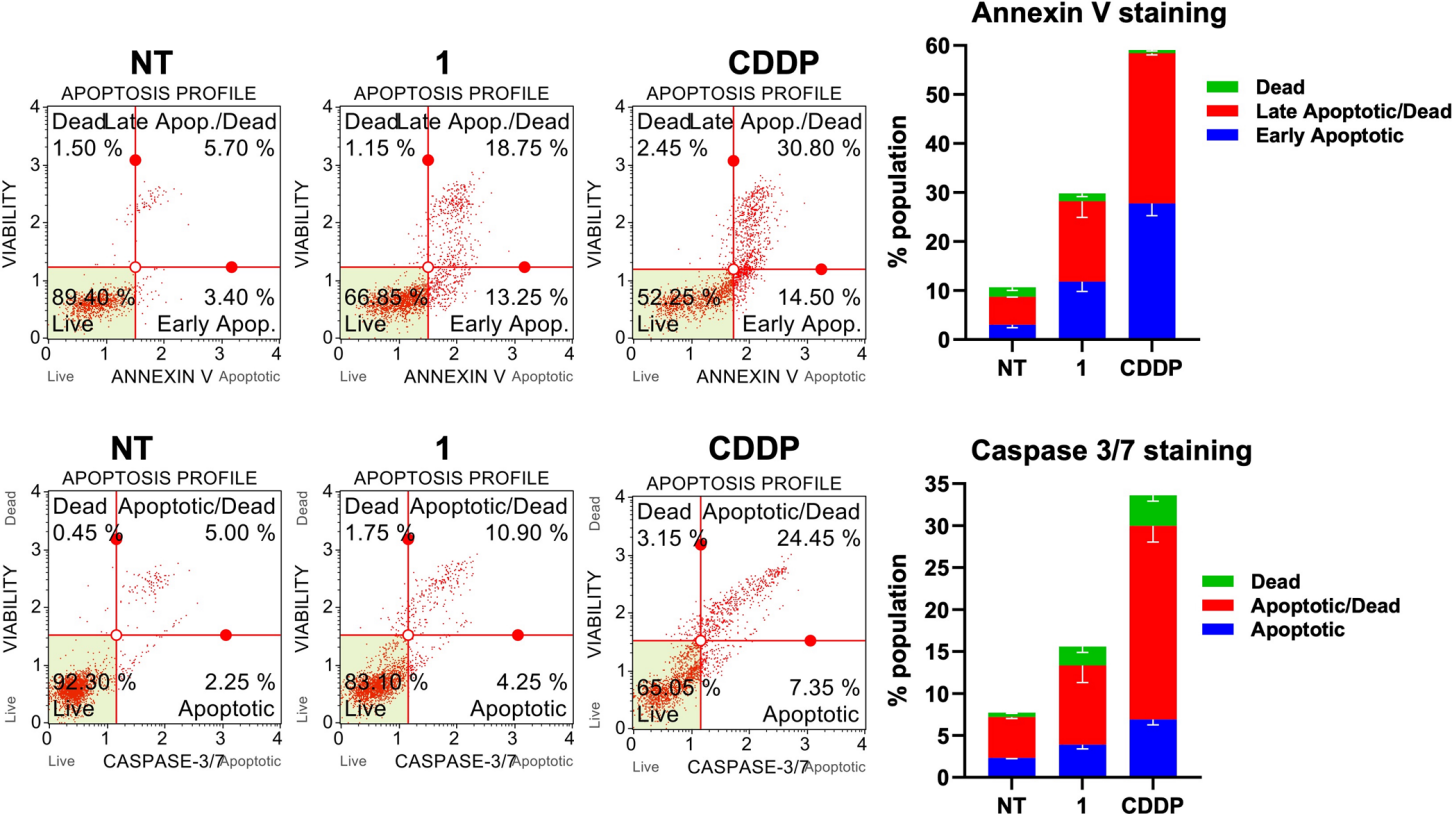
Flow cytometric analysis to assess induction of apoptosis by Annexin V/7-AAD positivity (top) and Caspase 3/7 activity (bottom). MDA-MB-231 cells were treated with compound **1** or CDDP at half inhibitory concentrations for 72 h and analyzed using the Muse Cell Analyzer.

Lastly, PARP cleavage was used to assess apoptosis, as it serves as a hallmark of caspase activation and confirms the apoptotic process by indicating DNA damage response inactivation (Figure 9). The results once again supported apoptosis as the primary form of cell death, with a clear increase in cleaved PARP observed at levels comparable to those seen with cisplatin. Consistent findings were again observed in MCF-7 cells (Figure S45).

**Figure 9:**
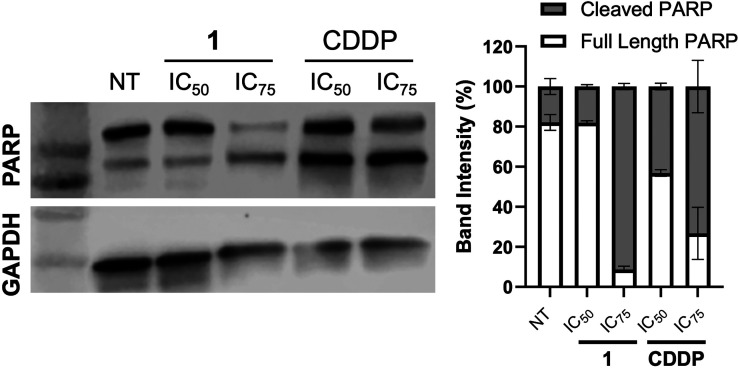
Quantification of PARP cleavage activation as an apoptosis marker after MDA-MB-231 cells were treated for 48 h with IC_50_ and IC_75_ doses of compound **1** and CDDP as a positive control. Left panel shows the Western blot images of cleaved and full-length PARP while the graph displays the quantification of band intensities.

### Three-Dimensional (3D) Cell Culture

3D cell cultures of cancer are good models for preclinical cancer research, as they are more representative of tumor microenvironments.^31^ MDA-MB-231 cells were maintained using the standard cell culture methods before seeding into round-bottom ultralow-attachment plates for 3D culture. Once the cells were seeded, the plate was centrifuged to assist with cell clumping at the bottom of the well and facilitate uniform spheroid formation. After 24 h, medium containing collagen I was added, and the cells were allowed to aggregate further. After sufficient growth of the spheroids (Day 4 – Figure 10), the cells were treated with 100 μM of **1** for 48 h. The higher concentration was chosen, as it has been reported that spheroids require higher drug doses than two-dimensional (2D) experiments.

**Figure 10:**
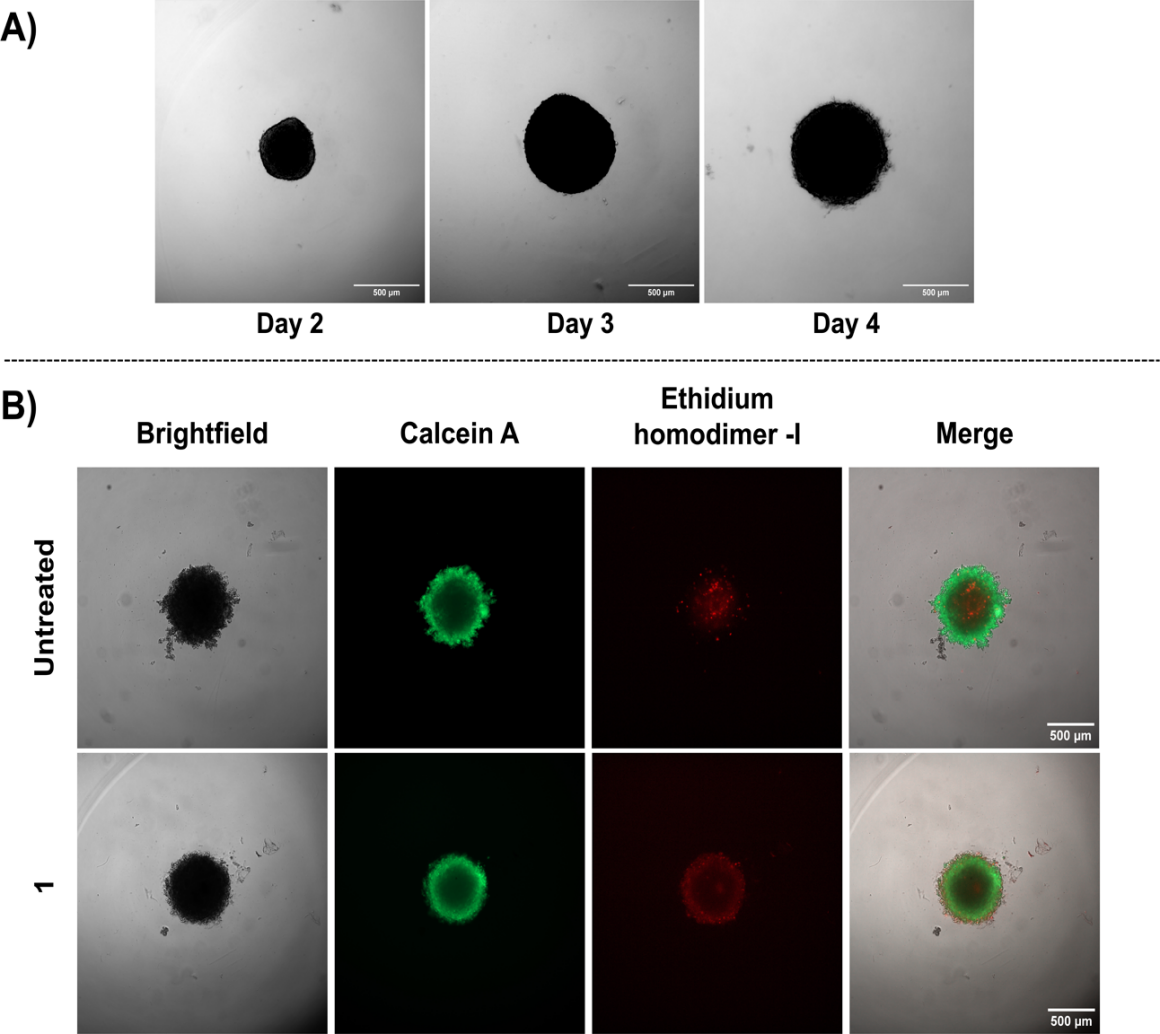
(A) Brightfield monitoring of 3D spheroid growth size over 4 days and (B) Brightfield and fluorescent images of 3D spheroids using a live/dead viability assay kit after 48 h of treatment with 100 μM of compound **1** (bottom row) or untreated cells (top row). The green fluorescent Calcein A stains proliferating cells, and red ethidium homodimer-I stains dead cells. Microscope images were collected using a Zeiss Observer 7 inverted microscope. Scale bar = 500 μm.

Live and dead cells were then visualized using the LIVE/DEAD Viability/Cytotoxicity Kit by Invitrogen. The green fluorescent Calcein A stains proliferating cells, and red ethidium homodimer-I stains dead cells. Based on the images in Figure 10, the treated sample shows more dead cells, evidenced by increased red fluorescence, particularly at the spheroid’s periphery, compared to the control. This suggests that the compound is effective at targeting peripheral cells, likely due to greater drug availability in the outer layer. Given that the tumor core often lacks oxygen and is naturally more prone to cell death, the ability to target and affect the peripheral cells becomes especially crucial for effective treatment.

## Conclusions

Four charged Cp*-Ir­(III) complexes of the type [Cp*Ir­(**L1–4**)­(PTA)]­(PF_6_) (where **L1–4** = a functionalized β-ketoiminate ligand and PTA = 1,3,5-triaza-7-phosphaadamantane ligand) have been successfully synthesized and characterized. NMR studies demonstrate excellent stability of the compounds in DMSO-*d*_6_/D_2_O mixtures, even in the presence of NaCl, as no changes are observed over 96 h. However, when exposed to fully supplemented media at 37 °C, decomposition is evident and dependent on the substitution pattern, with compounds **1** and **3** being the most stable, which fully decompose after 90 and >96 h, respectively. Their cytotoxicity values have been determined against a range of cancerous and noncancerous cell lines and highlight high cytotoxicity and selectivity toward breast cancers.

This study provides insights into the DNA interaction and apoptotic effects of the most active compound **1**, comparing its efficacy with that of cisplatin (CDDP). Cell death assessments indicated that **1** effectively induces apoptosis, as evidenced by increased staining with AO/EtBr, Annexin V positivity, caspase activation, and PARP cleavage. Morphological analysis further confirmed typical apoptotic features such as DNA condensation and fragmentation, with **1** demonstrating greater apoptotic induction than CDDP in both MDA-MB-231 and MCF-7 cells. These findings suggest that **1** is capable of triggering apoptosis through mechanisms involving caspase activation and DNA damage response inactivation, with similar mechanisms of action as other reported Cp*-Ir­(III) complexes.^32,33^

The 3D spheroid models offered a more realistic evaluation of the potential for **1** to act as an effect anticancer agent, showing significant cytotoxicity, particularly targeting the peripheral cells of the spheroid. This observation highlights the compound’s ability to impact regions where drug availability is higher, making it a promising candidate for further development.

Overall, the findings suggest that compound **1** has potential as an anticancer agent, selectively inducing apoptosis in cancer cells while showing distinct response kinetics compared to cisplatin. Further studies are needed to better understand its mechanism of action, optimize its efficacy, and confirm its potential for clinical applications.

## Experimental Section

The general synthetic method and characterization data of compounds **1–4** are stated here, and all other general methods, protocols, spectroscopic analysis, and biological assays are given in the Supporting Information.

### General Synthetic Method of Compounds **1–4**

β-ketominate ligands **L1–4** were all prepared according to our previous literature methods.^17–19^ Compounds **1–4** were prepared by stirring the functionalized ligand **L1–4** (2 equiv) and triethylamine (2 equiv) in dichloromethane (10 mL). The mixture was stirred at RT for 10 min before adding [Cp*IrCl_2_]_2_ (1 equiv), and stirring was continued overnight. The solvent was removed *in vacuo*, resuspended in ethanol (10 mL), and 1,3,5-triaza-7-phosphaadamantane (PTA, 2 equiv) added. The mixture was stirred at RT for 1 h, before adding ammonium hexafluorophosphate (2 equiv) and stirring was continued for 30 min. An orange precipitate formed, which was filtered and washed with cold ethanol and diethyl ether and left to dry under reduced pressure.

### Characterization of Compounds **1–4**

#### [Cp*Ir­(C_16_H_14_NO)­(PTA)]­(PF_6_) (**1**)

**L1** (24.9 mg, 0.105 mmol); Et_3_N (15 μL, 0.108 mmol); [Cp*IrCl_2_]_2_ (40.4 mg, 0.0507 mmol); PTA (16.7 mg, 0.106 mmol); NH_4_PF_6_ (17.0 mg, 0.104 mmol) were used. ***Yield:*** 58 mg, 0.067 mmol, 64%; ^1^H NMR ((CD_3_)_2_CO, 500 MHz, 298 K, δ): 8.06–8.03 (m, 2H, Ar–CH), 7.56 (m, 3H, Ar–CH), 7.49 (app tt, 2H, ^3^ *J*(^1^H–^1^H) = 7.8 Hz, ^4^ *J*(^1^H–^1^H) = 1.4 Hz Ar–CH), 7.28 (tt, 1H, ^3^ *J*(^1^H–^1^H) = 7.5 Hz, ^4^ *J*(^1^H–^1^H) = 1.2 Hz, Ar–CH), 7.21 (br. d, 2H, ^3^ *J*(^1^H–^1^H) = 7.6 Hz, Ar–CH), 6.01 (s, 1H, methine CH), 4.65–4.53 (m, 6H, NCH_2_N PTA), 4.48 (s, 6H, PCH_2_N PTA), 1.92 (s, 3H, CH_3_), 1.49 (d, 15H, ^4^ *J*(^1^H–^31^P) = 2.3 Hz, Cp*-CH_3_); ^31^P­{^1^H} NMR ((CD_3_)_2_CO, 202 MHz, 298 K, δ): −61.35 (s, 1P, PTA), −144.25 (sept, 1P, ^1^ *J*(^31^P–^19^F) = 705 Hz, PF_6_); ^13^C­{^1^H} NMR ((CD_3_)_2_CO, 100 MHz, 298 K, δ): 170.2 (Q, C–O), 166.7 (Q, C–N), 153.2 (Q, Ar–C), 138.6 (Q, Ar–C), 131.4 (Ar–CH), 129.7 (Ar–CH), 127.6 (Ar–CH), 127.5 (Ar–CH), 100.8 (methine CH), 95.7 (Q, d, ^2^ *J*(^13^C–^31^P) = 2.2 Hz, Cp*-C), 73.5 (d, ^3^ *J*(^13^C–^31^P) = 5.8 Hz, NCH_2_N PTA), 50.4 (d, ^1^ *J*(^13^C–^31^P) = 13.5 Hz, PCH_2_N PTA), 25.6 (CH_3_), 9.1 (Cp*-CH_3_); HR-MS calculated for the complex cation C_32_H_41_IrN_4_OP^+^ (*m*/*z*, 100%): 721.2643; Found: 721.2647; Elemental analysis calculated for C_32_H_41_F_6_IrN_4_OP_2_: C 44.39, H 4.77, N 6.47%, Found: C 44.05, H 4.74, N 6.38%.

#### [Cp*Ir­(C_16_H_12_Cl_2_NO)­(PTA)]­(PF_6_) (**2**)

**L2** (22.8 mg, 0.0745 mmol); Et_3_N (10 μL, 0.717 mmol); [Cp*IrCl_2_]_2_ (29.2 mg, 0.0366 mmol); PTA (11.3 mg, 0.0719 mmol); NH_4_PF_6_ (12.1 mg, 0.0745 mmol) were used. Yield: 44 mg, 0.047 mmol, 63%; ^1^H NMR ((CD_3_)_2_CO, 500 MHz, 298 K, δ): 7.65 (d, 1H, ^3^ *J*(^1^H–^1^H) = 8.3 Hz, Ar–CH), 7.61 (d, 1H, ^4^ *J*(^1^H–^1^H) = 2.1 Hz, Ar–CH), 7.61–7.53 (br. s, 2H, Ar–CH), 7.50 (dd, 1H, ^3^ *J*(^1^H–^1^H) = 8.3 Hz, ^4^ *J*(^1^H–^1^H) = 2.1 Hz, Ar–CH), 7.28 (br. tt, 1H, ^3^ *J*(^1^H–^1^H) = 7.4 Hz, ^4^ *J*(^1^H–^1^H) = 1.2 Hz, Ar–CH), 7.22 (br. d, 2H, ^3^ *J*(^1^H–^1^H) = 7.8 Hz, Ar–CH), 5.42 (s, 1H, methine CH), 4.71–4.63 (m, 6H, NCH_2_N PTA), 4.53 (s, 6H, PCH_2_N PTA), 1.87 (s, 3H, CH_3_), 1.46 (d, 15H, ^4^ *J*(^1^H–^31^P) = 2.4 Hz, Cp*-CH_3_); ^31^P­{^1^H} NMR ((CD_3_)_2_CO, 202 MHz, 298 K, δ): −61.05 (s, 1P, PTA), −144.26 (sept, 1P, ^1^ *J*(^31^P–^19^F) = 705 Hz, PF_6_); ^13^C­{^1^H} NMR ((CD_3_)_2_CO, 100 MHz, 298 K, δ): 170.5 (Q, C–O), 166.9 (Q, C–N), 153.0 (Q, ArC-N), 138.7 (Q, ArC-O), 135.9 (Q, ArC-Cl), 132.2 (Q, ArC-Cl), 131.7 (Ar–CH), 130.7 (Ar–CH), 128.8 (Ar–CH), 127.9 (Ar–CH), 105.7 (methine CH), 95.8 (Q, d, ^2^ *J*(^13^C–^31^P) = 2.1 Hz, Cp*-C), 73.5 (d, ^3^ *J*(^13^C–^31^P) = 5.8 Hz, NCH_2_N PTA), 50.20 (d, ^1^ *J*(^13^C–^31^P) = 13.6 Hz, PCH_2_N PTA), 25.5 (CH_3_), 9.2 (Cp*-CH_3_); HR-MS calculated for the complex cation C_32_H_39_Cl_2_IrN_4_OP^+^ (*m*/*z*, 100%): 789.1849; Found: 789.1856; Elemental analysis calculated C_32_H_39_Cl_2_F_6_IrN_4_OP_2_: C 41.12, H 4.21, N, 5.99%, Found: C 41.21, H 3.87, N 5.82%.

#### [Cp*Ir­(C_16_H_13_BrNO)­(PTA)]­(PF_6_) (**3**)

**L3** (19.8 mg, 0.0625 mmol); Et_3_N (10 μL, 0.0717); [Cp*IrCl_2_]_2_ (24.9 mg, 0.0313 mmol); PTA (9.80 mg, 0.0624 mmol); NH_4_PF_6_ (10.2 mg, 0.0626 mmol) were used. Yield: 27 mg, 0.029 mmol, 46%; ^1^H NMR ((CD_3_)_2_CO, 500 MHz, 298 K, δ): 8.00 (app. dt, 2H, ^3^ *J*(^1^H–^1^H) = 8.7 Hz, ^4^ *J*(^1^H–^1^H) = 2.6 Hz Ar–CH), 7.66 (app. dt, 2H, ^3^ *J*(^1^H–^1^H) = 8.7 Hz, ^4^ *J*(^1^H–^1^H) = 2.6 Hz, Ar–CH), 7.56 (br. s, 2H, Ar–CH), 7.28 (tt, 1H, ^3^ *J*(^1^H–^1^H) = 7.5 Hz, ^4^ *J*(^1^H–^1^H) = 1.1 Hz, Ar–CH), 7.20 (br. d, 2H, ^3^ *J*(^1^H–^1^H) = 7.8 Hz, Ar–CH), 6.02 (s, 1H, methine CH), 4.65–4.54 (m, 6H, NCH_2_N PTA), 4.46 (s, 6H, PCH_2_N PTA), 1.91 (s, 3H, CH_3_), 1.48 (d, 15H, ^4^ *J*(^31^P–^1^H) = 2.3 Hz, Cp*-CH_3_); ^31^P­{^1^H} NMR ((CD_3_)_2_CO, 202 MHz, 298 K, δ): −61.35 (s, 1P, PTA), −144.25 (sept, 1P, ^1^ *J*(^31^P–^19^F) = 705 Hz, PF6); ^13^C­{^1^H} NMR ((CD_3_)_2_CO, 100 MHz, 298 K, δ): 168.6 (Q, C–O), 166.9 (Q, C–N), 153.2 (Q, ArC-N), 137.8 (Q, ArC-O), 132.8 (Ar–CH), 129.5 (Ar–CH), 127.7 (Ar–CH), 125.3 (Q, ArC-Br), 100.7 (methine CH), 95.7 (Q, d, ^2^ *J*(^13^C–^31^P) = 2.2 Hz, Cp*-C), 73.5 (d, ^3^ *J*(^13^C–^31^P) = 5.9 Hz, NCH_2_N PTA), 50.3 (d, ^1^ *J*(^13^C–^31^P) = 13.5 Hz, PCH_2_N PTA), 25.6 (CH_3_), 9.1 (Cp*-CH_3_); HR-MS calculated for the complex cation C_32_H_40_BrIrN_4_OP^+^ (*m*/*z*, 100%): 799.1732; Found: 799.1739; Elemental analysis calculated C_32_H_40_BrF_6_IrN_4_OP_2_: C 40.68, H 4.27, N 5.93%, Found: C 40.66, H 3.86, N 5.74%.

#### [Cp*Ir­(C_18_H_18_NO_2_)­(PTA)]­(PF_6_) (**4**)

**L4** (23.7 mg, 0.0842 mmol); Et_3_N (11 μL, 0.0789 mmol); [Cp*IrCl_2_]_2_ (32.2 mg, 0.0404 mmol); PTA (13.2 mg, 0.0840 mmol); NH_4_PF_6_ (13.6 mg, 0.0834 mmol) were used. Yield: 53 mg, 0.058 mmol, 69%; ^1^H NMR ((CD_3_)_2_CO, 500 MHz, 298 K, δ): 8.01 (app. dt, 2H, ^3^ *J*(^1^H–^1^H) = 9.0 Hz, ^4^ *J*(^1^H–^1^H) = 3.0 Hz, Ar–CH), 7.59–7.50 (br s, 2H, Ar–CH), 7.26 (tt, 1H, ^3^ *J*(^1^H–^1^H) = 7.4 Hz, ^4^ *J*(^1^H–^1^H) = 1.0, Ar–CH), 7.19 (br. dd, 2H, ^3^ *J*(^1^H–^1^H) = 7.2 Hz, ^4^ *J*(^1^H–^1^H) = 1.3 Hz, Ar–CH), 7.00 (app. dt, 2H, ^3^ *J*(^1^H–^1^H) = 8.9 Hz, ^4^ *J*(^1^H–^1^H) = 3.1 Hz, Ar–CH), 5.94 (s, 1H, methine CH), 4.65–6.51 (m, 6H, NCH_2_N PTA), 4.45 (s, 6H, PCH_2_N PTA), 4.14 (q, 2H, ^3^ *J*(^1^H–^1^H) = 7.0 Hz, Ar-OCH_2_CH_3_), 1.90 (s, 3H, CH_3_), 1.48 (d, 15H, ^4^ *J*(^31^P–^1^H) = 2.3 Hz, Cp*-CH_3_), 1.40 (t, 3H, ^3^ *J*(^1^H–^1^H) = 7.0 Hz, Ar-OCH_2_CH_3_); ^31^P­{^1^H} NMR ((CD_3_)_2_CO, 202 MHz, 298 K, δ): −61.43 (s, 1P, PTA), – 144.25 (sept, 1P, ^1^ *J*(^31^P–^19^F) = 705 Hz, PF6); ^13^C­{^1^H} NMR ((CD_3_)_2_CO, 100 MHz, 298 K, δ): 170.2 (Q, C–O), 166.2 (Q, C–O), 162.2 (Q, ArC-N), 153.3 (Q, ArC-O), 130.6 (Q, ArC-O), 129.4 (Ar–CH), 127.4 (Ar–CH), 126.3 (Q, Ar–C), 115.4 (Ar–CH), 99.7 (methine CH), 95.6 (Q, d, ^2^ *J*(^13^C–^31^P) = 2.2 Hz, Cp*-C), 73.45 (d, ^3^ *J*(^13^C–^31^P) = 6.1 Hz, NCH_2_N PTA), 64.5­(Ar-OCH_2_CH_3_), 50.4 (d, ^1^ *J*(^13^C–^31^P) = 13.6 Hz, PCH_2_N PTA), 25.5 (CH_3_), 15.2 (Ar-OCH_2_CH_3_), 9.1 (Cp*-CH_3_); HR-MS calculated for the complex cation C_34_H_45_IrN_4_O_2_P^+^ (*m*/*z*, 100%): 765.2906; Found: 765.2912; Elemental analysis calculated for C_34_H_45_F_6_IrN_4_O_2_P_2_·H_2_O: C 44.01, H 5.11, N 6.04%, Found: C 43.79, H 4.65, N 5.81%.

## Supplementary Material



## Notes

General methods; NMR spectroscopy; scxXRD data; NMR stability studies in 90/10 DMSO-*d*_6_/D_2_O; stability of compounds **1-4** in supplemented media over 96 h; high resolution mass spectrometry for compounds **1–4** (PDF)

## Abbreviations

AO: acridine orange
ARPE-19: noncancerous retinal epithelial cells
CARB: carboplatin
CDDP: cisplatin
Cp*: 1,2,3,4,5-pentamethylcyclopentadienyl
DCF: dichlorofluorescein
DNA: DNA
EtBr: ethidium bromide
H_2_DCFDA: 2′,7′-dichlorodihydrofluorescein diacetate
IC_50_: half maximal inhibitory concentration
MCF-7: epithelial breast adenocarcinoma
MDA-MB-231: epithelial breast adenocarcinoma
MIA PaCa-2: epithelial pancreas carcinoma
NMR: nuclear magnetic resonance
PARP: poyl­(ADP-ribose) polymerase
PF_6_: hexafluorophosphate
PGM: platinum group metals
PTA: 1,3,5-triaza-7-phosphaadamantane
ROS: reactive oxygen species
scXRD: single crystal X-ray diffraction
SD: standard deviation
SEM: standard error of measurement
SI: selectivity index
