# Supplementary material for: Understanding the Modes of Action of β‑Ketoiminato Iridium(III) Complexes in Cancer Cells
Source: Inorg Chem. 2025 Aug 22;64(34):17189–99. doi: 10.1021/acs.inorgchem.5c02026 (PMC12406195; doi:10.1021/acs.inorgchem.5c02026)
Supplement: Supplementary file 1 [file ic5c02026_si_001.pdf]

Supporting Information

# Understanding the Modes of Action of β-Ketoiminate Iridium(III) Complexes in Cancer Cells

*Tameryn Stringer,‡[b,c] Busra Yildirim,‡[d,f] Baris Sergi,[d,f] Benjamin J. Hofmann,[a] Yi-Hsuan Lee,[b] Ceyda Acilan*[e,f] and Rianne M. Lord*[a,b]*

[a]Department of Chemistry, University of Warwick, Coventry, CV4 7AL, United Kingdom; Rianne.lord@warwick.ac.uk; [b]School of Chemistry, Pharmacy and Pharmacology, University of East Anglia, Norwich, Norfolk, NR4 7TJ, United Kingdom; [c]School of Science, The University of Waikato, Hamilton, 3210, New Zealand; [d]Graduate School of Health Sciences, Koç University, Istanbul, 34450, Turkey; [e]Koç University Translational Research Center, KUTTAM, Istanbul, 34450, Turkey; [f]School of Medicine, Koç University, Sariyer, Istanbul, 34450, Turkey; cayhan@ku.edu.tr

# Table of Contents


*Experimental* ..... *S4*

General Methods ..... S4

Single Crystal X-ray Diffraction ..... S4

*Protocols* ..... *S4*

High Resolution Mass Spectrometry ..... S4

Stability Assay by NMR Spectroscopy ..... S5

Stability Assays by UV/vis Spectroscopy ..... S5

Cell Viability Assays ..... S5

Reactive Oxygen Species ..... S6

DNA Interactions ..... S6

Acridine Orange/Ethidium Bromide (AO/EB) Assay ..... S6

Nuclei Morphology Assay ..... S6

Western Blot ..... S6

Flow Cytometry Analyses ..... S7

Annexin-V Staining ..... S7

Caspase 3/7 Activation Staining ..... S7

3D Cell Culture ..... S7

*NMR Spectroscopy* ..... *S8*

Compound 1 ..... S8

Compound 2 ..... S10

Compound 3 ..... S12

Compound 4 ..... S14

*NMR Stability Studies in 90/10 DMSO-$d_6$/$D_2O$* ..... *S16*

Compound 1 ..... S16

Compound 2 ..... S18

Compound 3 ..... S20

Compound 4 ..... S22

*NMR Stability Studies in 0.1 M NaCl 70/30 DMSO-$d_6$/$D_2O$* ..... *S24*

Compound 1 ..... S24

Compound 2 ..... S26

Compound 3 ..... S28

Compound 4 ..... S30

*Stability of Compounds 1-4 in Supplemented Media over 96 hours* ..... *S32*

*High Resolution Mass Spectrometry for Compounds 1-4* ....... *S34*
*High Resolution Mass Spectrometry for Compounds 1-4 after 96 hours in DMSO* ....... *S36*
*Single Crystal X-ray Diffraction* ....... *S39*
*Chemosensitivity Studies* ....... *S40*
*LogP Predictions* ....... *S42*
*Results against the MCF-7 cell line* ....... *S43*
DNA Morphology with Compound 1 ....... S43
Induction of Apoptosis with Compound 1 ....... S44
Quantification of PARP with Compound 1 ....... S44
*References* ....... *S45*

## Experimental

### General Methods

Chemicals were purchased from Sigma-Aldrich (Merck KGaA), Fischer Scientific and Fluorochem and used without further purification. $^{1}H$, $^{13}C$ and $^{31}P$ NMRs are recorded on a Bruker Avance III 500 (Ascent 500) and referenced to TMS using the respective residual solvent signal as secondary standard. The spectra are processed in MestReNova 14.0.1, and multiplicities are abbreviated as s = singlet, d = doublet, t = triplet, q = quartet, br = broad, m = multiplet or respective combinations. UV/vis spectra were collected using Jasco V-730 spectrometer equipped with a PAC-743R temperature control unit, and the data were processed using Origin 2024b. HR-MS samples were measured on a Bruker Compact Q-TOF mass spectrometer, and the samples analyzed in positive ion mode in a mass scan range of 50–3000 *m/z* through direct infusion. Ionization settings: nebulizer gas 0.3 bar, desolation gas ($N_2$) 4 L/min, dry temperature 200 °C, capillary voltage of -4000 V, capillary end plate offset of 500 V. Sodium formate (10 mM) was used for calibration.

### Single Crystal X-ray Diffraction

A suitable single crystal was selected and immersed in fomblin. The crystal was then mounted to a goniometer head on an XtaLAB Synergy Dualflex, HyPix diffractometer fitted with a Hybrid Pixel Array Detector and a goniometer head, using mirror monochromated Mo-Kα radiation (λ = 0.71073 Å) source. The crystal was cooled to 100 K by an Oxford cryostream low-temperature device.[1] The full data set was recorded, and the images processed using CrysAlis Pro.[2] Structure solution by direct methods was achieved through the use SHELXT and SHELXL programs,[3] and the structural model refined by full matrix least squares on $F^2$ using the program Olex2 1.5.[4] Hydrogen atoms were placed using idealized geometric positions (with free rotation for methyl groups), allowed to move in a "riding model" along with the atoms to which they were attached, and refined isotropically. Editing of the CIFs and construction of tables of bond lengths and angles were also achieved using Olex2 1.5. All molecular images were generated using Mercury 4.0, and the crystal data were uploaded to the CCDC, with submission numbers 2392862-2392865.

## Protocols

### High Resolution Mass Spectrometry

The compounds were dissolved in DMSO (~8 mg/mL) and diluted to 1 µg/mL using a mixture of methanol and water (80/20). The samples were analyzed in positive ion mode in a mass scan range of 50–3000 *m/z* through direct infusion on a Bruker Compact Q-TOF mass spectrometer. Mass spectra of all compounds are shown in **Figures S33-S36**. Fresh samples were also made and left to stand in DMSO-

$d_6/D_2O$ mixture (70/30) spiked with 0.1 M NaCl for 96 hours, before HR-MS was conducted; these are shown in **Figures S37-S40**.

**Stability Assay by NMR Spectroscopy**

Compounds **1-4** (3-4 mg) were dissolved in a 500 μL mixture of 90% DMSO-$d_6$ and 10% $D_2O$. $^1H$ and $^{31}P$ NMR spectra were obtained by a Bruker Avance 500 MHz at room temperature and multiple time points over 4 days. A solution of sodium chloride in $D_2O$ (26.3 g/L, 143 μL) was added to the same samples (final concentration 0.1 M NaCl in 70 % DMSO-$d_6$ and 30% $D_2O$) to mimic the chloride concentration in blood. $^1H$ and $^{31}P$ NMR spectra were obtained by a Bruker Avance 500 MHz at room temperature and multiple time points over 4 days.

**Stability Assays by UV/vis Spectroscopy**

The compounds were dissolved in DMSO and added to fully supplemented DMEM phenol-red free media (see Cell Viability Assays section for all additives) under sterile conditions to obtain a final concentration of 60 μg/mL. UV/vis spectra were obtained every 30 min for 96 h at 37 °C.

**Cell Viability Assays**

All cytotoxicity assays were conducted using human cell lines: breast adenocarcinomas (MDA-MB-231 and MCF-7), pancreatic carcinoma (MIA PaCa-2) and non-cancerous epithelial retinal (ARPE-19) cell lines. All cell lines were routinely maintained as monolayer cultures in appropriate complete media: MCF-7, MIA PaCa-2 and ARPE-19 in high glucose DMEM and MDA-MB-231 in RPMI-1640, and both media were supplemented with 10% FBS, 1 mM sodium pyruvate, 2 mM L-glutamine, and 1% pen-strep. All cells were initially grown in T-25 or T-75 flasks at 37 °C and 5% $CO_2$. Before chemosensitivity studies, cell monolayers were passaged using trypsin-EDTA (0.05%) and diluted to a concentration of 1 x $10^4$ cells/mL (or 4 x $10^4$ cells mL for 24 h assays). All assays were conducted in 96-well plates, in which 100 μL of the cell suspension was added for 24 h at 37 °C and 5% $CO_2$, and then 100 μL of compound/medium dilutions for a further 96 h (or 24 h). All compound stocks were made using sterile DMSO at 100 mM before dilution, and a maximum of 0.1% DMSO was used in each assay. After incubation, 3-(4,5-dimethylthiazol-2-yl)-2,5-diphenyltetrazolium bromide (20 μL, 5 mg/mL in PBS) was added to each well and incubated for 3 h at 37°C and 5% $CO_2$. All solutions were then aspirated, DMSO (150 μL) added to each well, gently mixed, and the absorbance was measured at 540 nm using a ClarioStar spectrophotometer microplate reader. Results were plotted on a logarithmic scale, and the half maximal inhibitory concentration ($IC_{50}$) was determined from a triplicate of triplicate repeats (n = 9) and reported as an $IC_{50}$ ± Standard Deviation (SD).

**Reactive Oxygen Species**

MDA-MB-231 cells at 4000 cells/well were seeded into a black clear-bottom 96-well plate using phenol-red free complete RMPI medium, and incubated for 48 h. The cells were treated with 100 μM of **1** for 4 h alongside an untreated sample (control). 2′,7′-dichlorodihydrofluorescein diacetate ($H_2$DCFDA, 20 μM final concentration in PBS) was added for 45 min. The cells were washed with PBS (2 x 100 μL), and phenol-red free complete RMPI medium (100 μL) was added to each well. Fluorescence images were obtained using a Zeiss Observer 7 inverted microscope at excitation at 488 nm and emission at 509 nm.

**DNA Interactions**

Plasmid DNA (200 ng, pBOS-H2B-GFP, BD Biosciences) was incubated overnight at room temperature with **1** or CDDP at concentrations between 100 and 400 μM in double-distilled $H_2O$ (20 μL total volume). After incubation, the samples were electrophoresed on a 1% agarose gel at 100 V for 60 min. The experiment was conducted in two biological replicates, and the band intensities were quantified and analyzed using ImageJ software.

**Acridine Orange/Ethidium Bromide (AO/EB) Assay**

MDA-MB-231 cells were seeded in 6-well plates at a density of $1.2 \times 10^5$ cells/well. After 48 h of treatment with **1** or CDDP, both floating and adherent cells were collected, centrifuged (400 g for 4 min), and resuspended in 25 μL PBS supplemented with 1% FBS. AO/EB staining solution (1 μL from a 100 μg mL mixture of AO and EB) was added to the cell suspension. The samples were then placed on microscope slides, covered with a coverslip, and their morphology was examined using a fluorescence microscope (Eclipse TS100, Nikon). Images were captured within 20 min of adding the AO/EB stain.

**Nuclei Morphology Assay**

MDA-MB-231 or MCF-7 cells were seeded onto glass slides at a density of 1 x $10^4$ cells/glass and 24 h later treated with **1** or CDDP at $IC_{50-75}$ concentrations for 48 h. Following treatment, cells were fixed with cold methanol for 10 min at room temperature. After fixation, the slides were briefly air-dried, and the cells were stained using DAPI mounting medium. Coverslips were placed on the slides, and the samples were examined under a fluorescence microscope (Leica DMI8) at 40X magnification. DNA shrinkage and fragmentation, indicative of apoptosis, were visualized, and images were captured.

**Western Blot**

MDA-MB-231 and MCF-7 cells were seeded in 6-well plates at a density of 2 x $10^5$ cells/well and after 24 h, treated with **1** or CDDP at $IC_{50-75}$ concentrations. After 48 h of treatment, proteins were isolated using a RIPA lysis buffer on ice for 30 min, and total protein concentrations were determined with the BCA assay. Equal amounts of protein from each sample (15 μg) were separated on SDS-PAGE gels and

transferred to PVDF membranes. The membranes were blocked with 5% non-fat dry milk in TBST and probed with primary antibodies against cleaved PARP (Cell Signaling Technologies, 5625S) and GAPDH (Abcam, ab313650, used as a housekeeping control). After incubation with the appropriate HRP-conjugated secondary antibodies, the proteins were visualized using an enhanced chemiluminescence (ECL) detection system.

**Flow Cytometry Analyses**

MDA-MB-231 and MCF-7 cells were seeded to a total of $4 \times 10^4$ cells/well in 12-well plates. After 24 h, the cells were treated with either **1** or CDDP at $IC_{50\text{-}75}$ concentrations. After 72 h of treatment, detached or trypsinized cells were collected and suspended in PBS containing 1% FBS at a concentration of 1 x $10^6$ cells/mL. The following analyses were performed according to the specified protocols.

**Annexin-V Staining**

An equal volume of Annexin V and 7-AAD solution was added to 100 µL of the cell suspension, followed by a 20 min incubation at room temperature. The cells were then analyzed using the Muse Cell Analyzer (Merck Millipore).

**Caspase 3/7 Activation Staining**

A mixture of 50 µL of cell suspension and 5 µL of Caspase 3/7 antibody (diluted 1:8 in PBS) was prepared and incubated at room temperature for 30 minutes. Then, 150 µL of the 7-AAD antibody solution (diluted 1:75 in 1× Assay Buffer) was added, followed by a 5 min incubation at room temperature. Apoptotic cells were analyzed using the Muse Cell Analyzer.

**3D Cell Culture**

MDA-MB-231 cells were seeded at 8000 cells/well in Nunclon™ Sphera™ 96-Well U-bottom microplates. The plate was centrifuged at 290 g for 3 min, and the cells were placed in the incubator for 24 h (Day 0). Complete medium containing collagen I (3 µg/mL final concentration) was added. The plate was centrifuged at 100 g for 3 min and placed back in the incubator (Day 1). Brightfield images were taken on Day 2, Day 3 and Day 4 to monitor the growth of the spheroids using the Zeiss Observer 7 inverted microscope. On Day 4, the cells were then subjected to treatment with **1** at 100 µM for 48 h, alongside an untreated control. Invitrogen™ LIVE/DEAD™ staining kit was then used as per the manufacturer's instructions to visualize live/dead cells. The spheroids were imaged and visualized using the Zeiss Observer 7 inverted microscope at ex/em 494/517 nm (green) and 528/617 nm (red).

## NMR Spectroscopy

### Compound 1

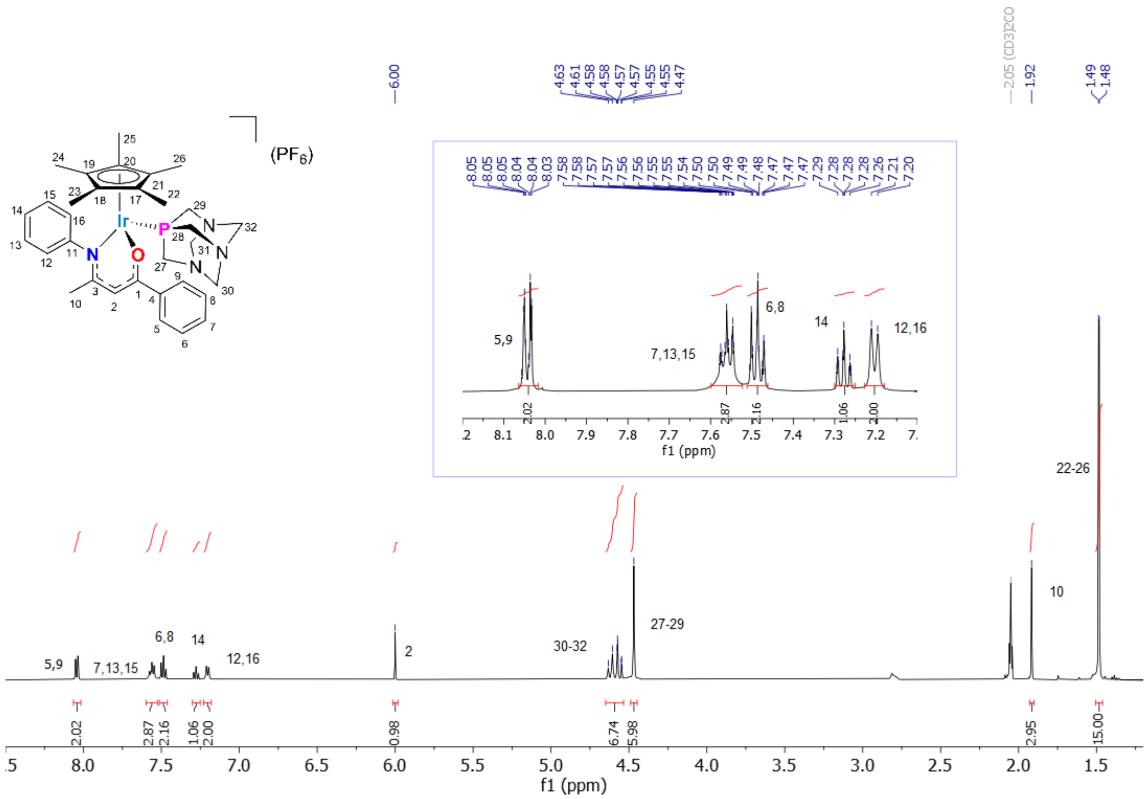

***Figure S 1.*** *¹H NMR spectrum of compound **1** ((CD$_3$)$_2$CO, 500 MHz, 298 K).*

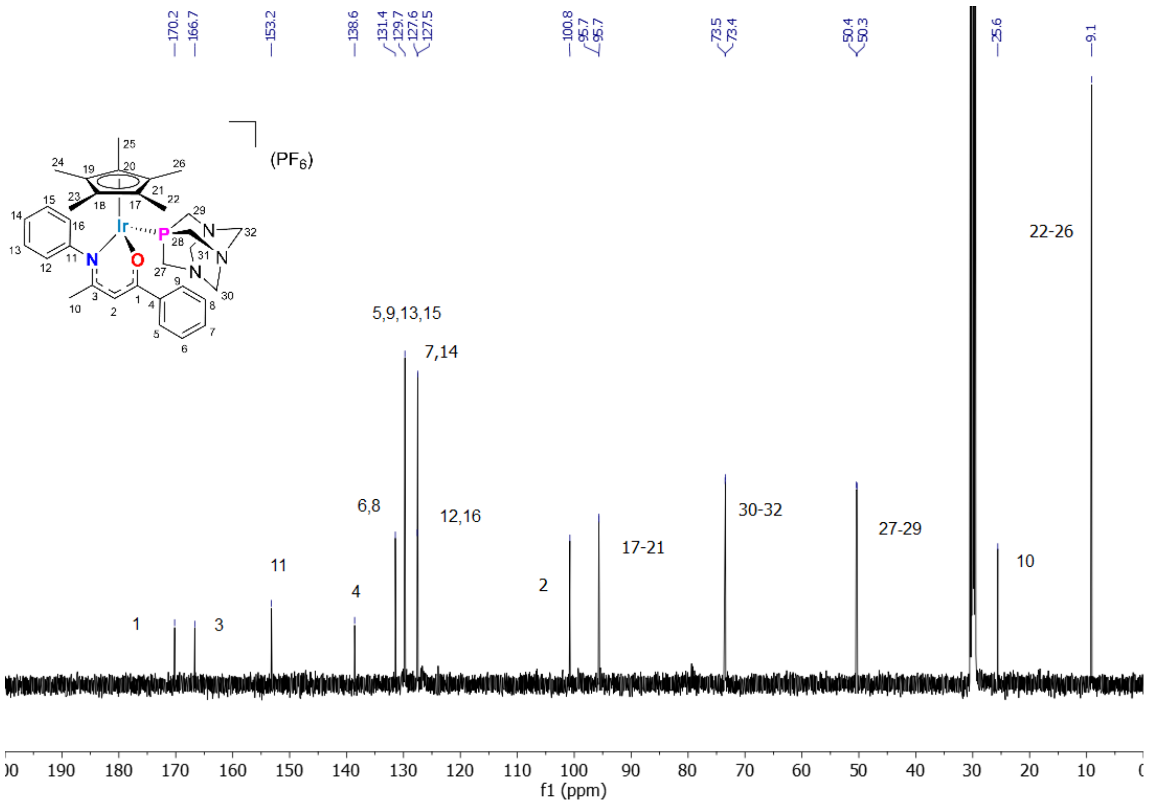

*Figure S 2.* $^{13}C\{^{1}H\}$ *NMR spectrum of compound* ***1*** *($(CD_3)_2CO$, 100 MHz, 298 K).*

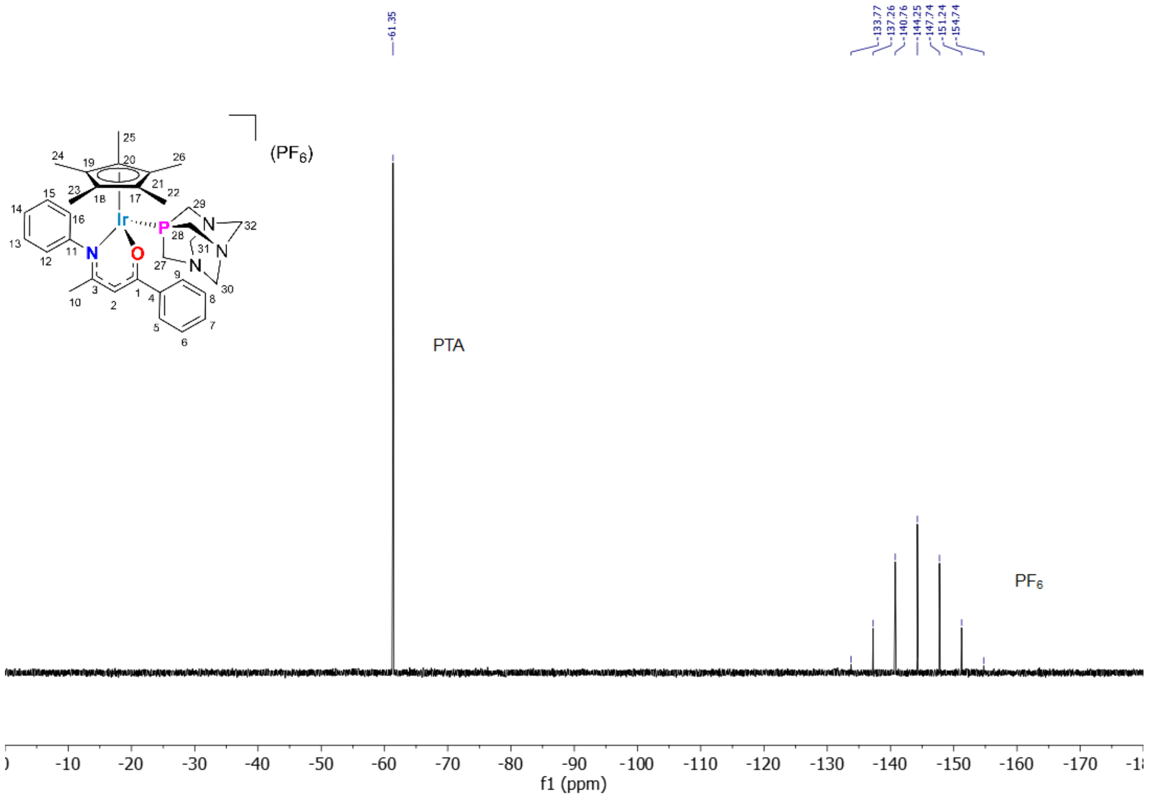

***Figure S 3.*** $^{31}P\{^{1}H\}$ *NMR spectrum of compound* ***1*** *($(CD_3)_2CO$, 202 MHz, 298 K).*

## Compound 2

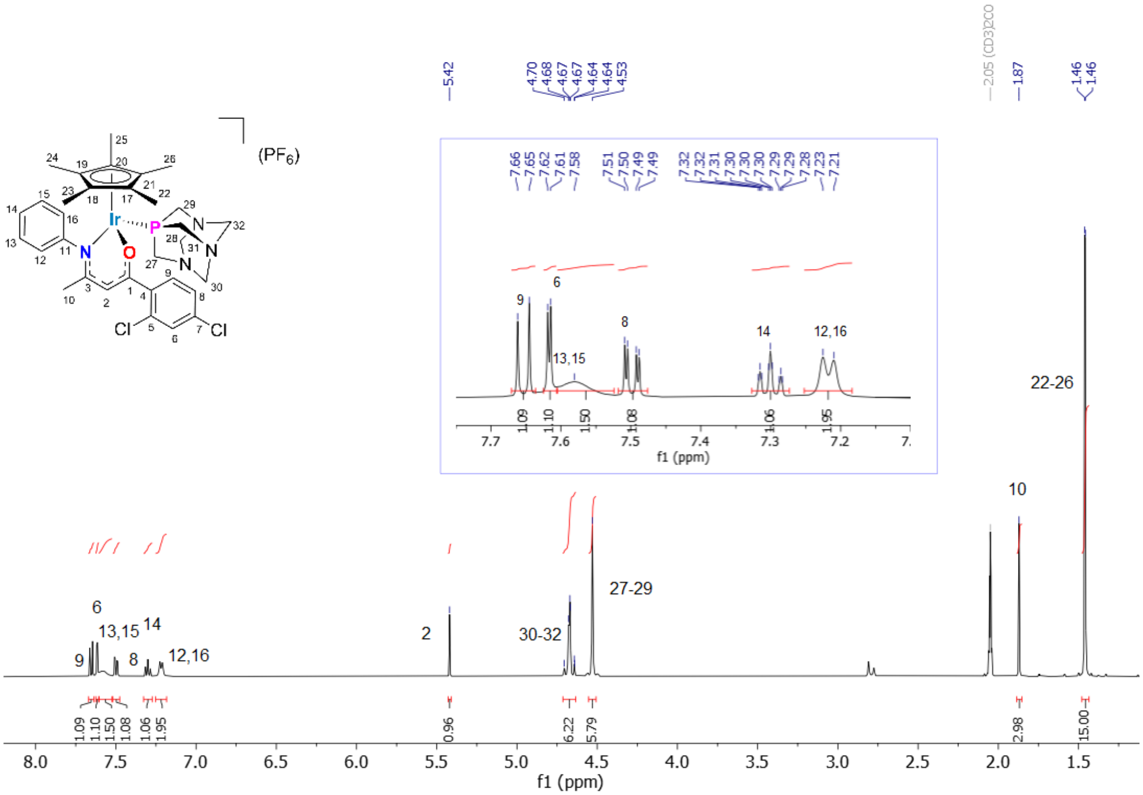


***Figure S 4.*** *$^{1}$H NMR spectrum of compound* ***2*** *($(CD_3)_2CO$, 500 MHz, 298 K).*

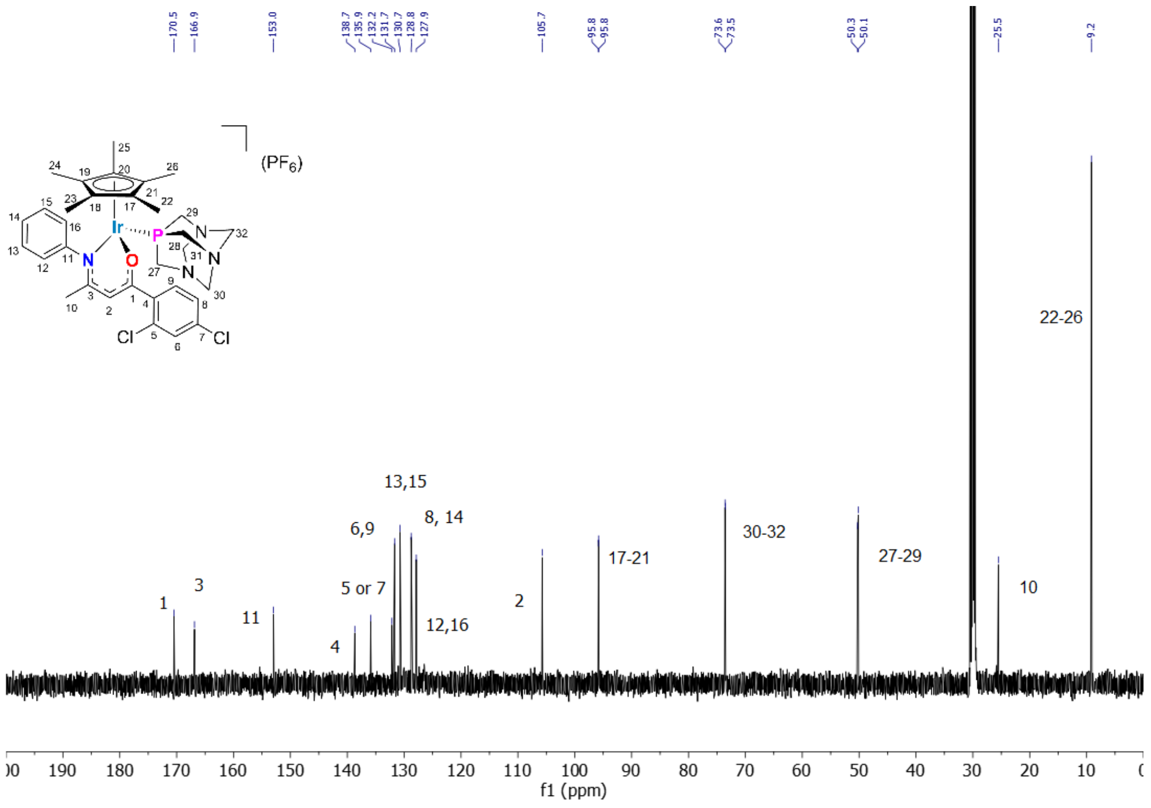


***Figure S 5.*** *$^{13}C\{^1H\}$ NMR spectrum of compound* ***2*** *($(CD_3)_2CO$, 100 MHz, 298 K).*

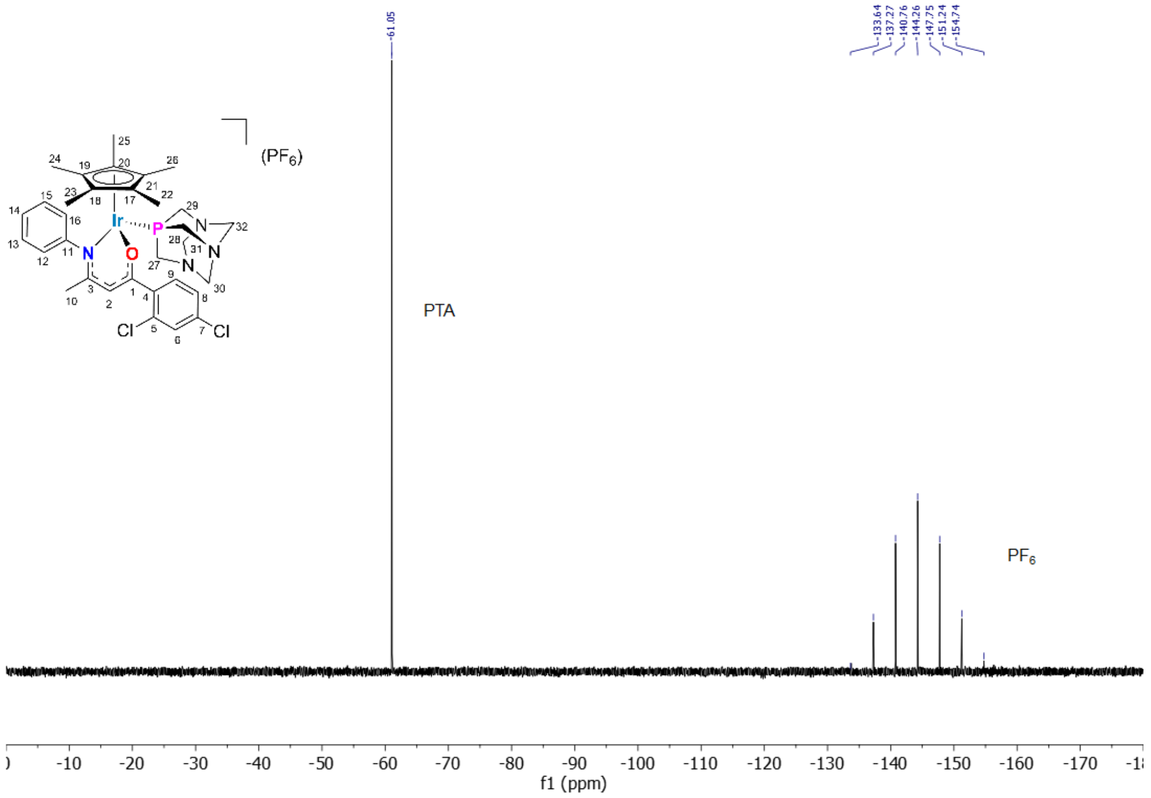


***Figure S 6.*** *$^{31}P\{^1H\}$ NMR spectrum of compound* ***2*** *($(CD_3)_2CO$, 202 MHz, 298 K).*

**Compound 3**

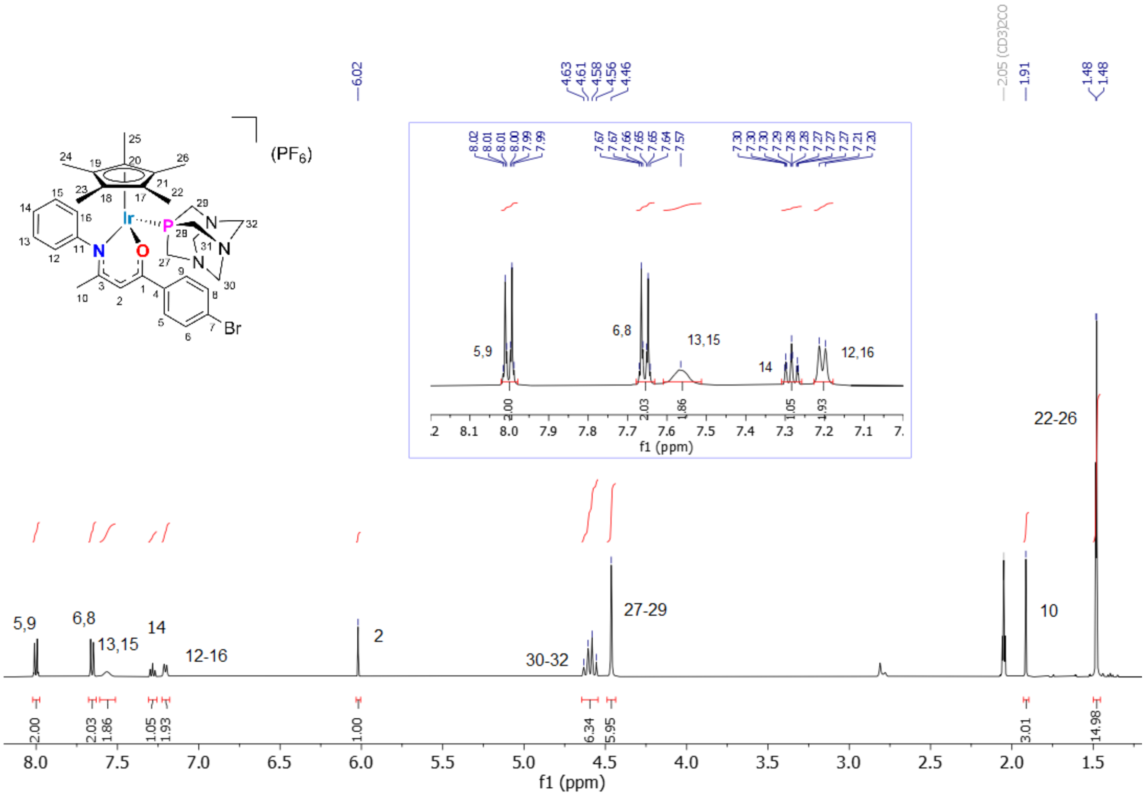

***Figure S 7.*** *$^{1}$H NMR spectrum of compound **3** ((CD$_3$)$_2$CO, 500 MHz, 298 K).*

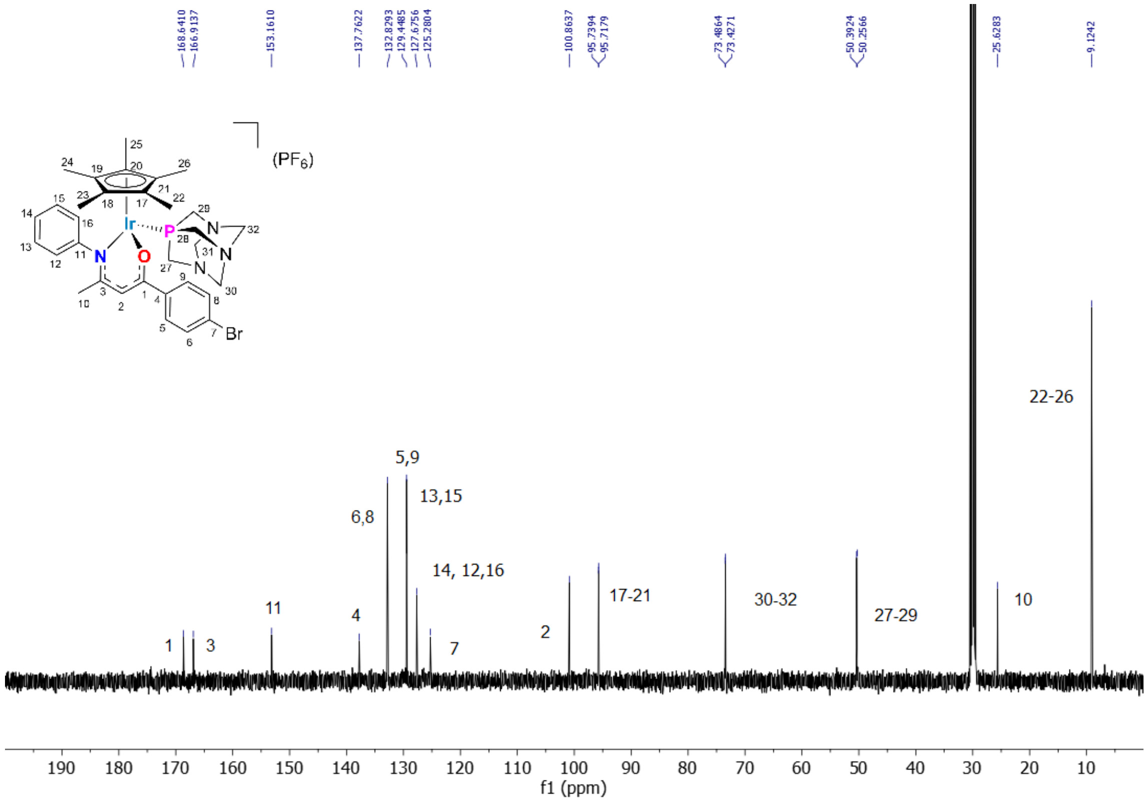


***Figure S 8.*** *$^{13}C\{^{1}H\}$ NMR spectrum of compound **3** ($(CD_3)_2CO$, 100 MHz, 298 K).*

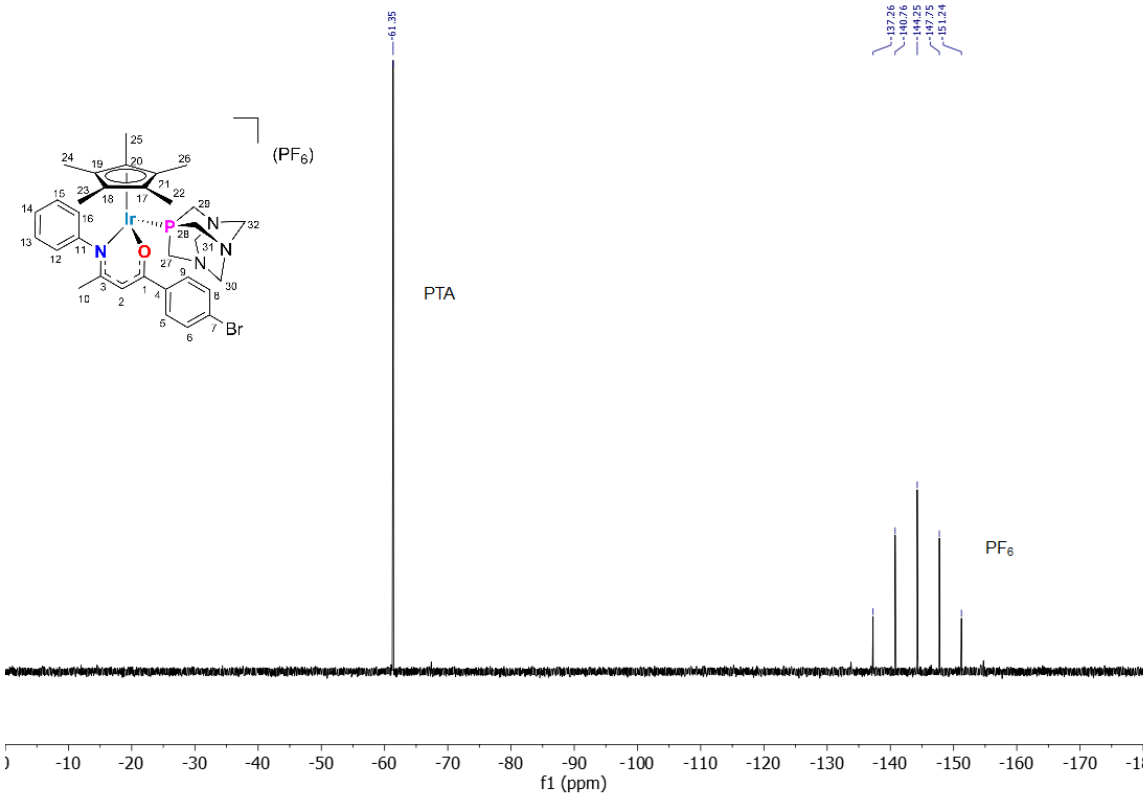


***Figure S 9.*** *$^{31}P\{^{1}H\}$ NMR spectrum of compound **3** ($(CD_3)_2CO$, 202 MHz, 298 K).*

### Compound 4

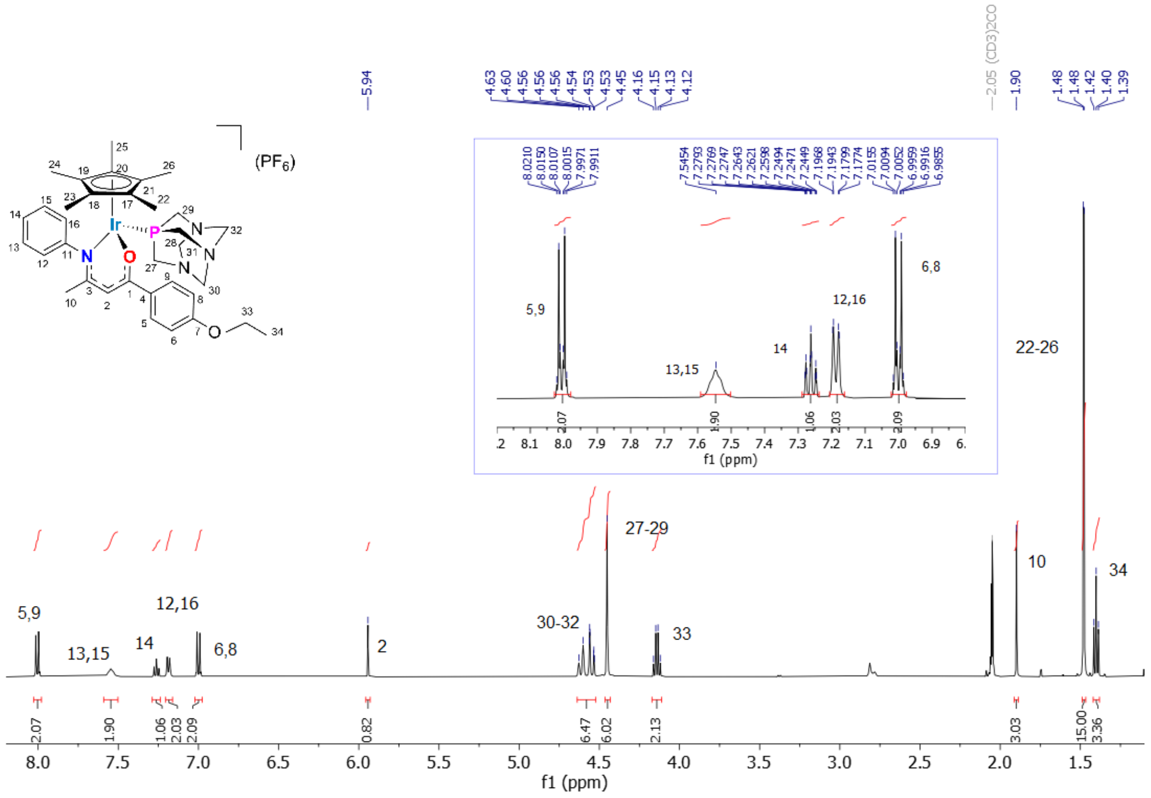

***Figure S 10.*** *$^{1}$H NMR spectrum of compound* ***4*** *($(CD_3)_2CO$, 500 MHz, 298 K).*

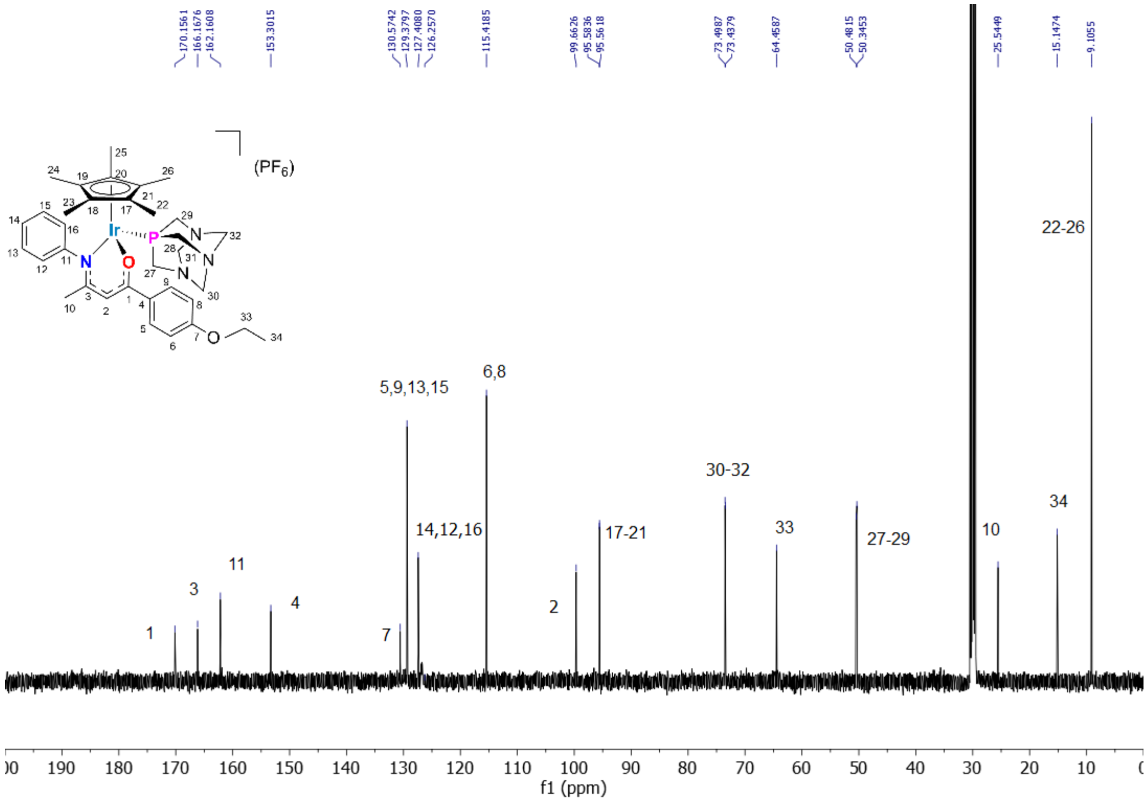


***Figure S 11.*** *$^{13}C\{^{1}H\}$ NMR spectrum of compound **4** ($(CD_3)_2CO$, 100 MHz, 298 K).*

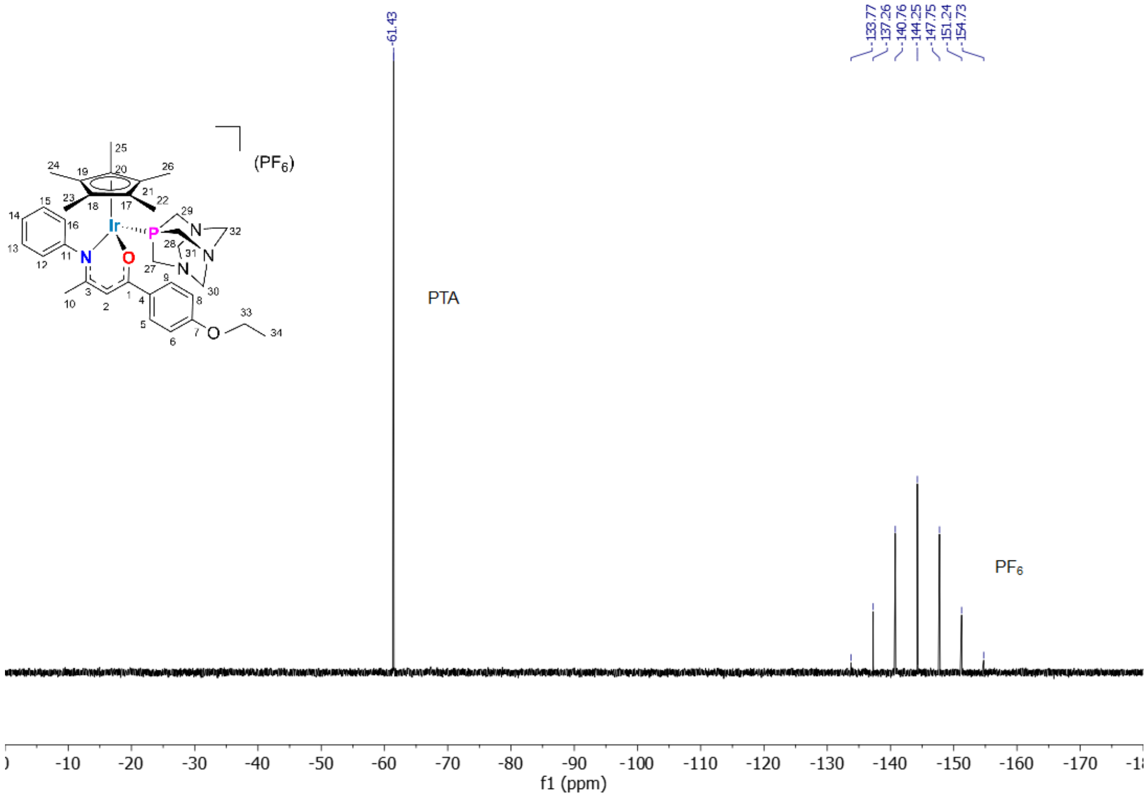


***Figure S 12.*** *$^{31}P\{^{1}H\}$ NMR spectrum of compound **4** ($(CD_3)_2CO$, 202 MHz, 298 K).*

## NMR Stability Studies in 90/10 DMSO-$d_6$/$D_2O$

### Compound 1

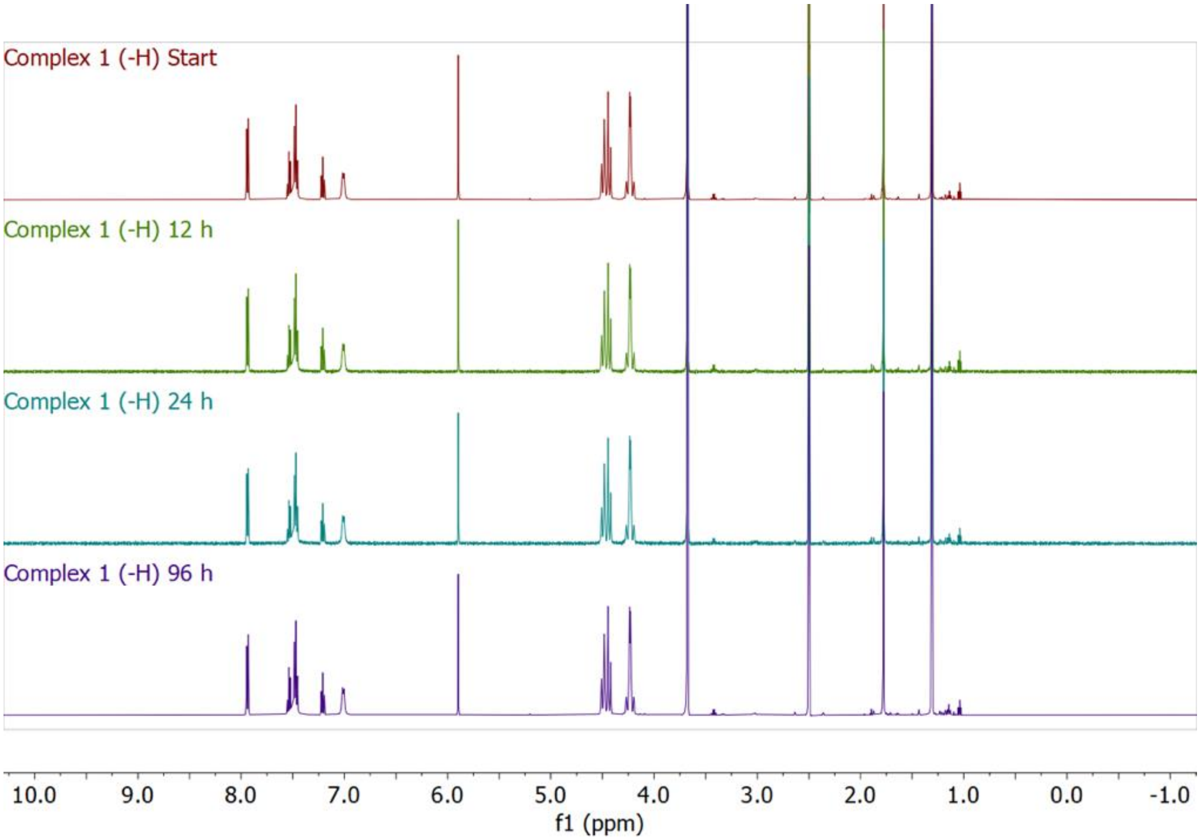


***Figure S 13.*** *$^1$H NMR spectra of compound **1** in 90% DMSO-$d_6$ and 10% $D_2O$. Spectra are shown for the start (red), then after 12 h (green), 24 h (blue) and 96 h (purple) (500 MHz, 298 K).*

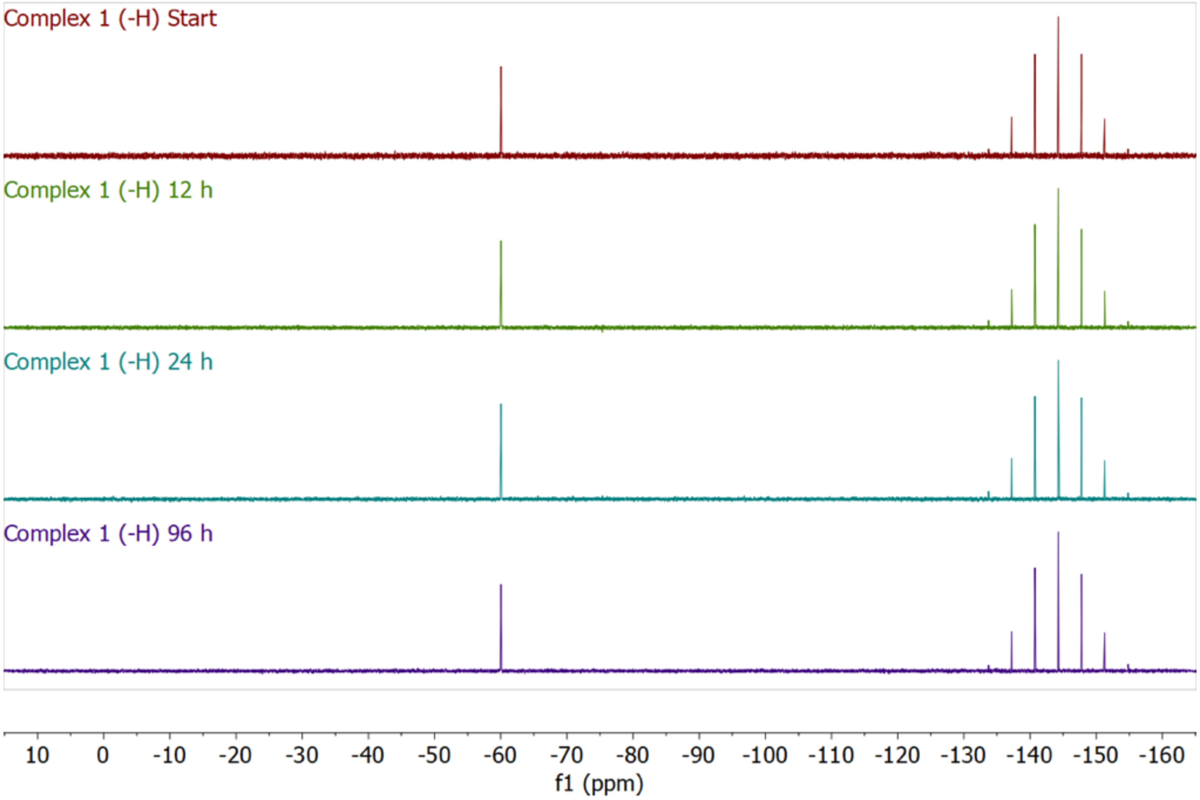


***Figure S 14.*** *$^{31}$P NMR spectra of compound **1** in 90% DMSO-$d_6$ and 10% $D_2O$. Spectra are shown for the start (red), then after 12 h (green), 24 h (blue) and 96 h (purple) (500 MHz, 298 K).*

**Compound 2**

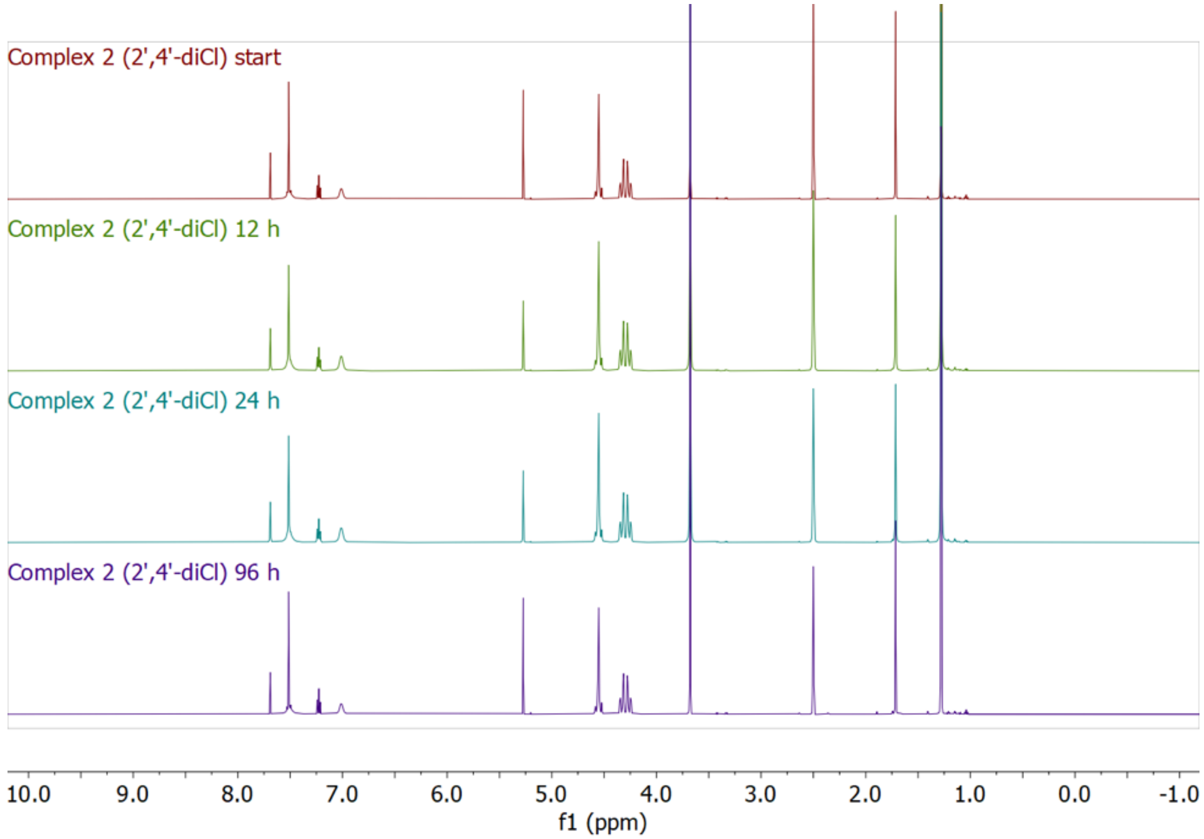


***Figure S 15.*** *$^{1}$H NMR spectra of compound* ***2*** *in 90% DMSO-d$_6$ and 10% $D_2O$. Spectra are shown for the start (red), then after 12 h (green), 24 h (blue) and 96 h (purple) (500 MHz, 298 K).*

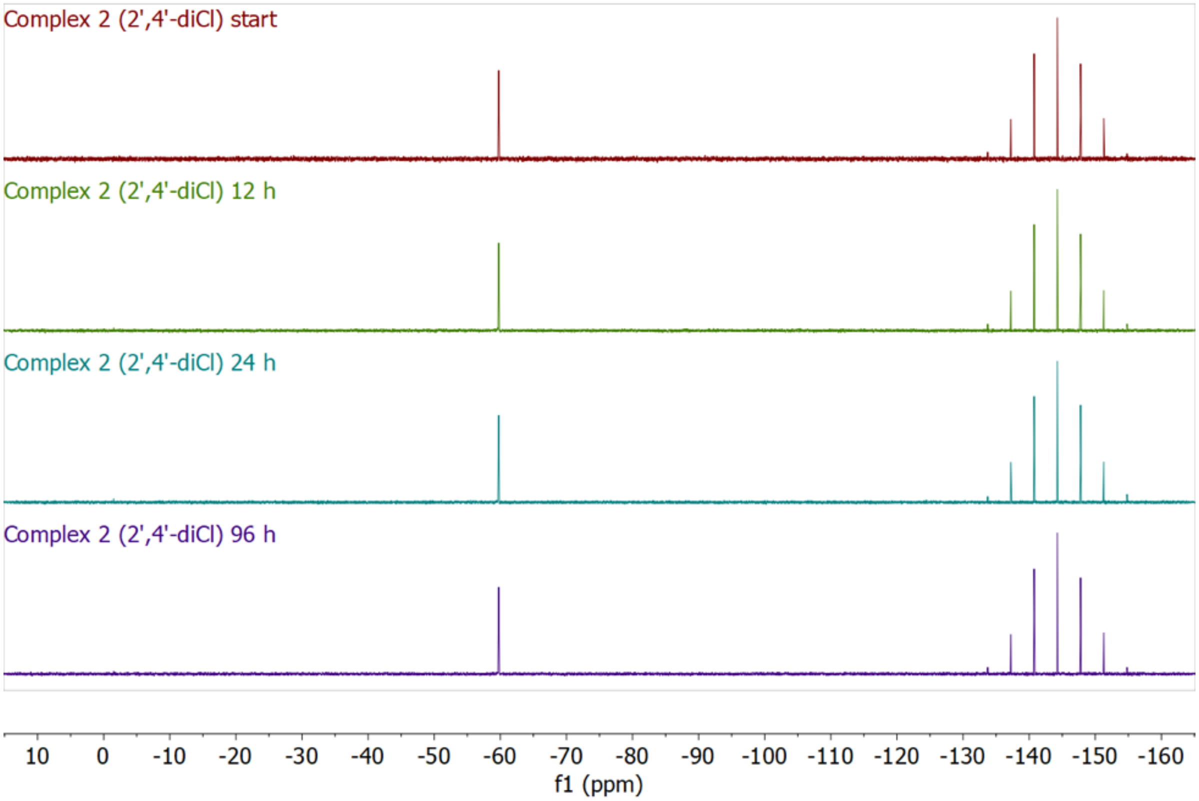


***Figure S 16.*** *$^{31}$P NMR spectra of compound* ***2*** *in 90% DMSO-$d_6$ and 10% $D_2O$. Spectra are shown for the start (red), then after 12 h (green), 24 h (blue) and 96 h (purple) (500 MHz, 298 K).*

**Compound 3**

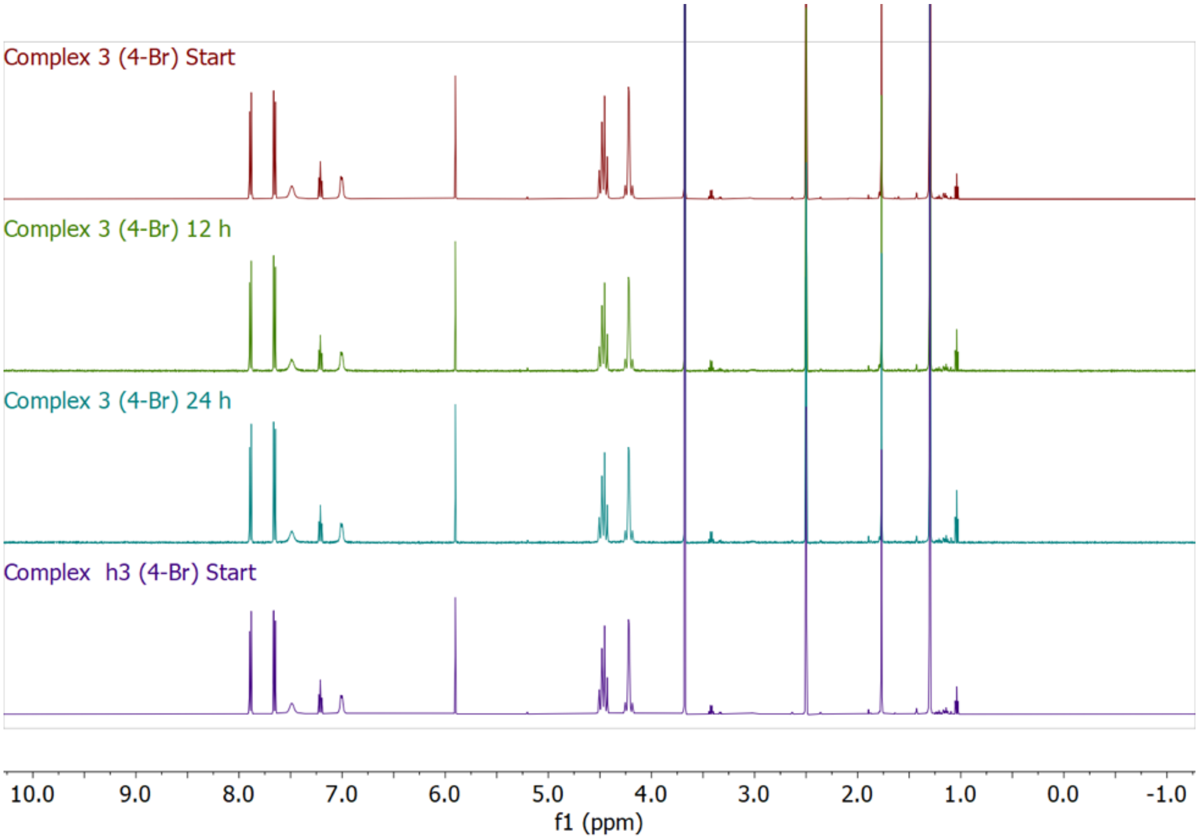


***Figure S 17.*** *$^{1}$H NMR spectra of compound **3** in 90% DMSO-$d_6$ and 10% $D_2O$. Spectra are shown for the start (red), then after 12 h (green), 24 h (blue) and 96 h (purple) (500 MHz, 298 K).*

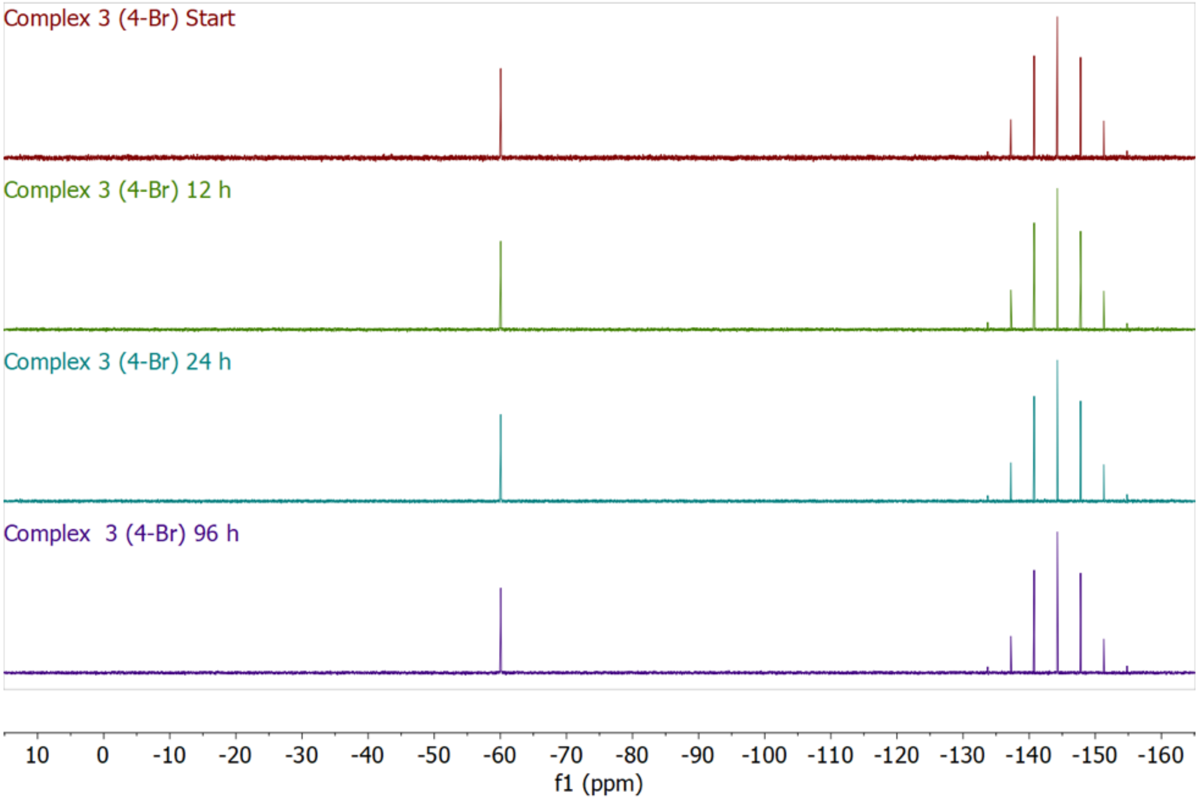


***Figure S 18.*** *$^{31}$P NMR spectra of compound* ***3*** *in 90% DMSO-$d_6$ and 10% $D_2O$. Spectra are shown for the start (red), then after 12 h (green), 24 h (blue) and 96 h (purple) (500 MHz, 298 K).*

**Compound 4**

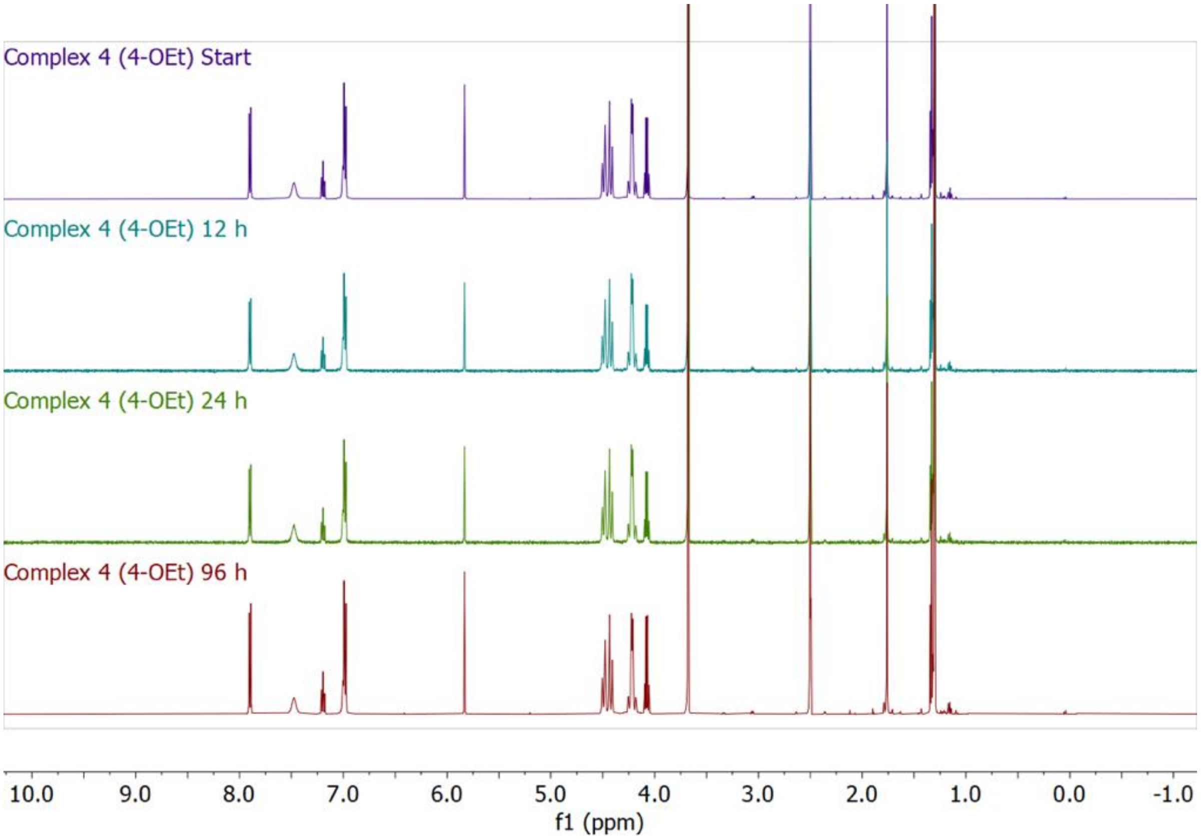


***Figure S 19.*** *$^{1}$H NMR spectra of compound **4** in 90% DMSO-$d_6$ and 10% $D_2O$. Spectra are shown for the start (red), then after 12 h (green), 24 h (blue) and 96 h (purple) (500 MHz, 298 K).*

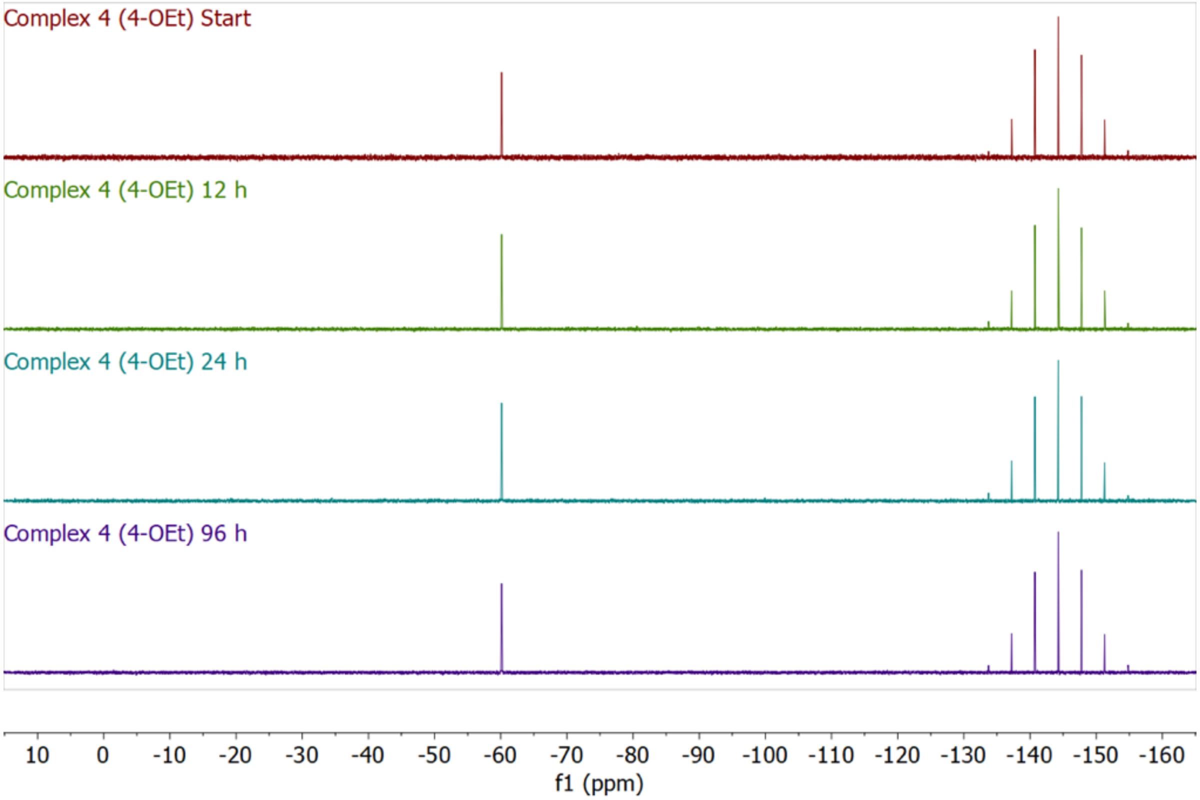


***Figure S 20.*** *$^{31}$P NMR spectra of compound **4** in 90% DMSO-$d_6$ and 10% $D_2O$. Spectra are shown for the start (red), then after 12 h (green), 24 h (blue) and 96 h (purple) (500 MHz, 298 K).*

## NMR Stability Studies in 0.1 M NaCl 70/30 DMSO-d$_6$/D$_2$O

### Compound 1

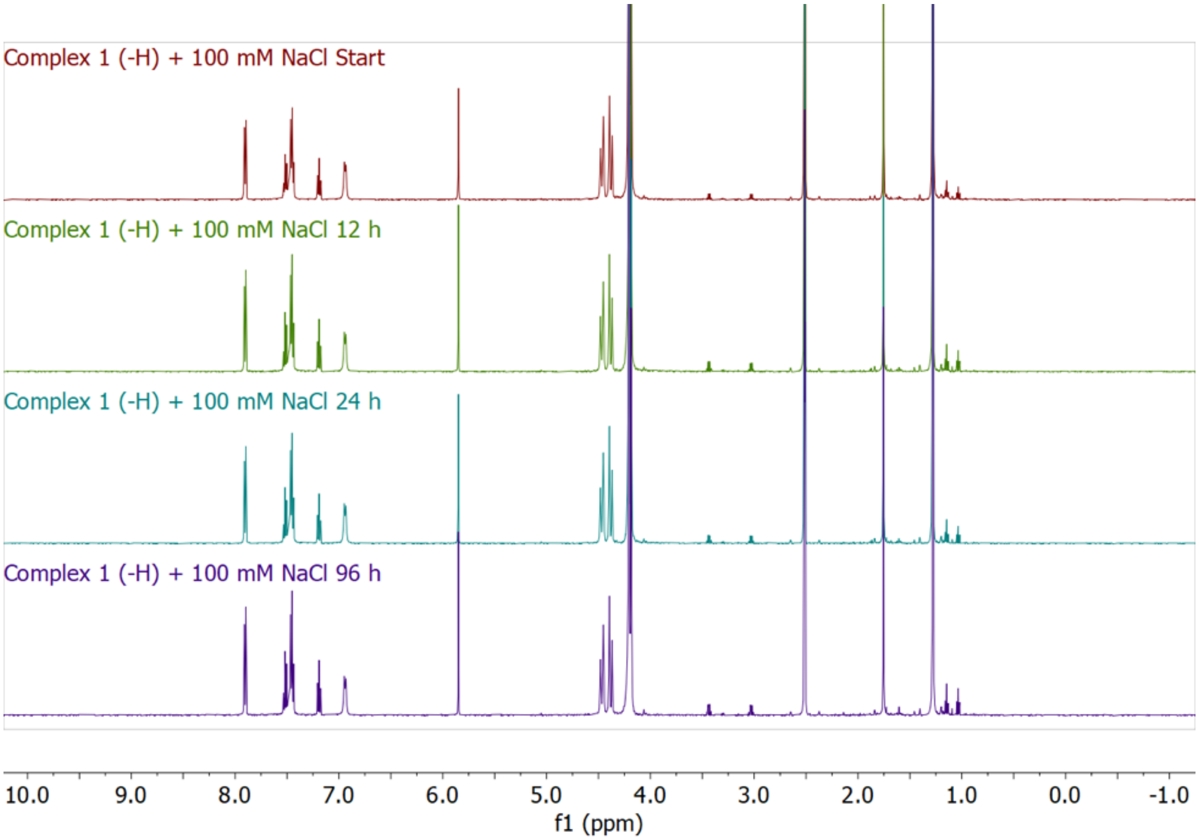


***Figure S 21.*** *$^1$H NMR spectra of compound **1** in a 0.1 M solution of NaCl in 70% DMSO-d$_6$ and 30% D$_2$O. Spectra are shown for the start (red), then after 12 h (green), 24 h (blue) and 96 h (purple) (500 MHz, 298 K).*

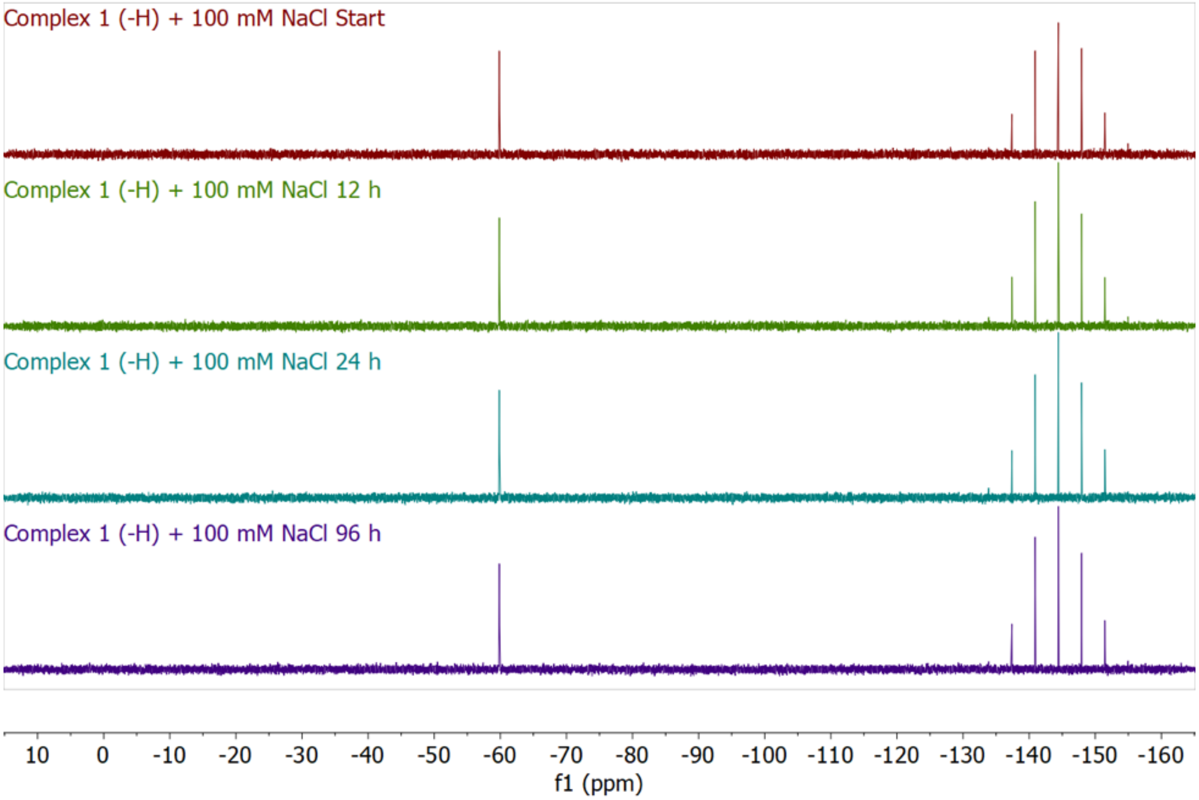


***Figure S 22.*** *$^{31}$P NMR spectra of compound* ***1*** *in a 0.1 M solution of NaCl in 70% DMSO-$d_6$ and 30% $D_2O$. Spectra are shown for the start (red), then after 12 h (green), 24 h (blue) and 96 h (purple) (500 MHz, 298 K).*

**Compound 2**

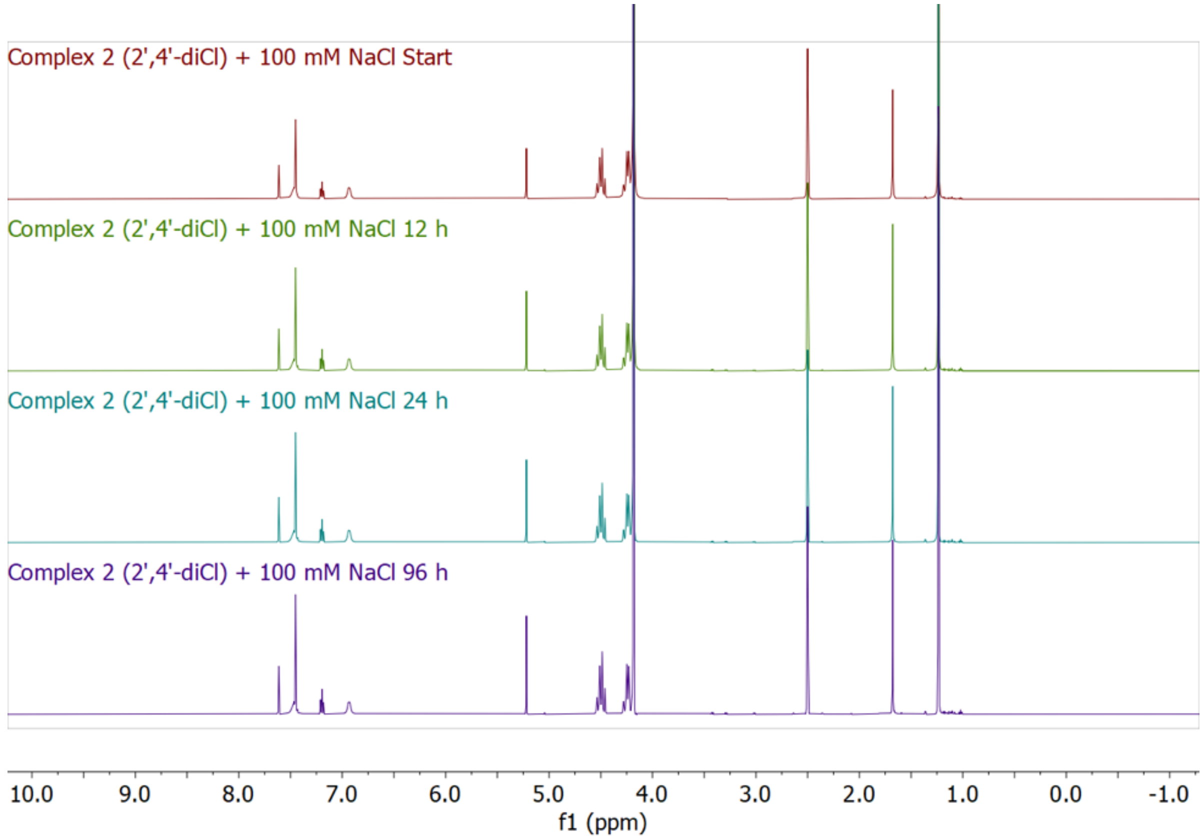


***Figure S 23.*** *$^{1}$H NMR spectra of compound **2** in a 0.1 M solution of NaCl in 70% DMSO-$d_6$ and 30% $D_2O$. Spectra are shown for the start (red), then after 12 h (green), 24 h (blue) and 96 h (purple) (500 MHz, 298 K).*

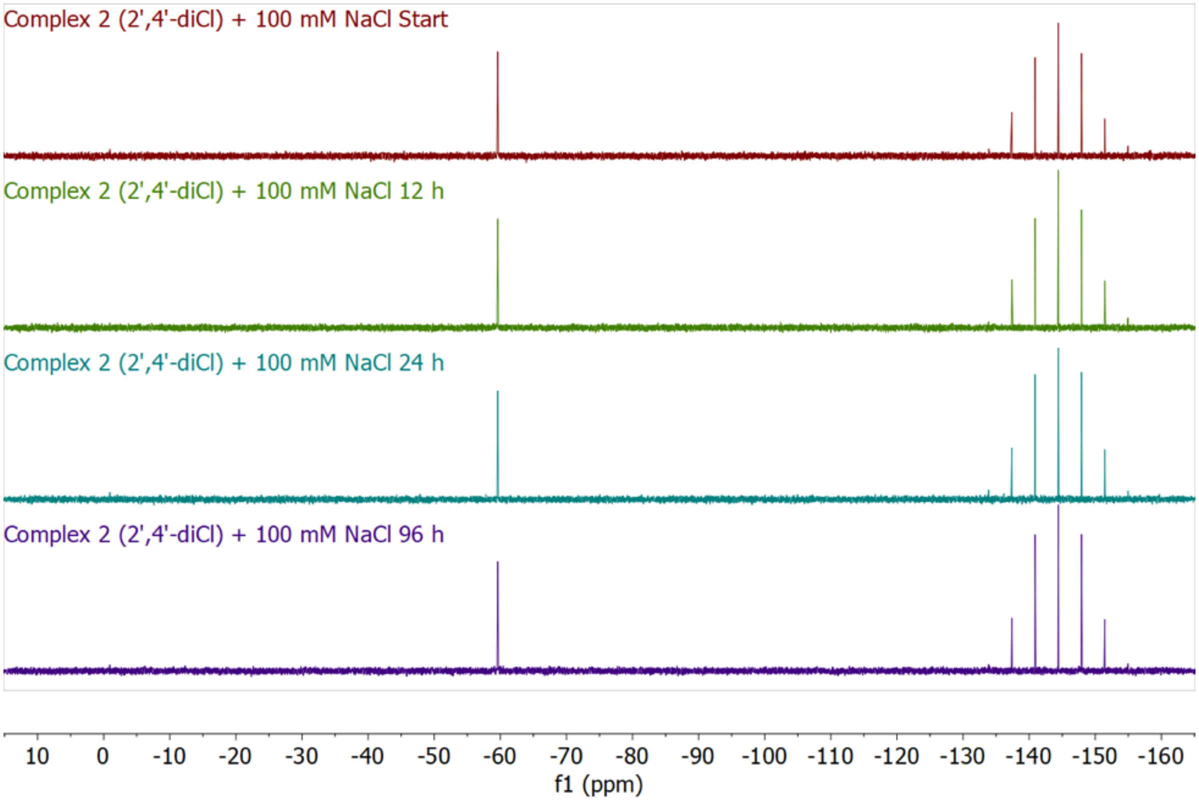


***Figure S 24.*** *$^{31}P$ NMR spectra of compound **2** in a 0.1 M solution of NaCl in 70% DMSO-$d_6$ and 30% $D_2O$. Spectra are shown for the start (red), then after 12 h (green), 24 h (blue) and 96 h (purple) (500 MHz, 298 K).*

**Compound 3**

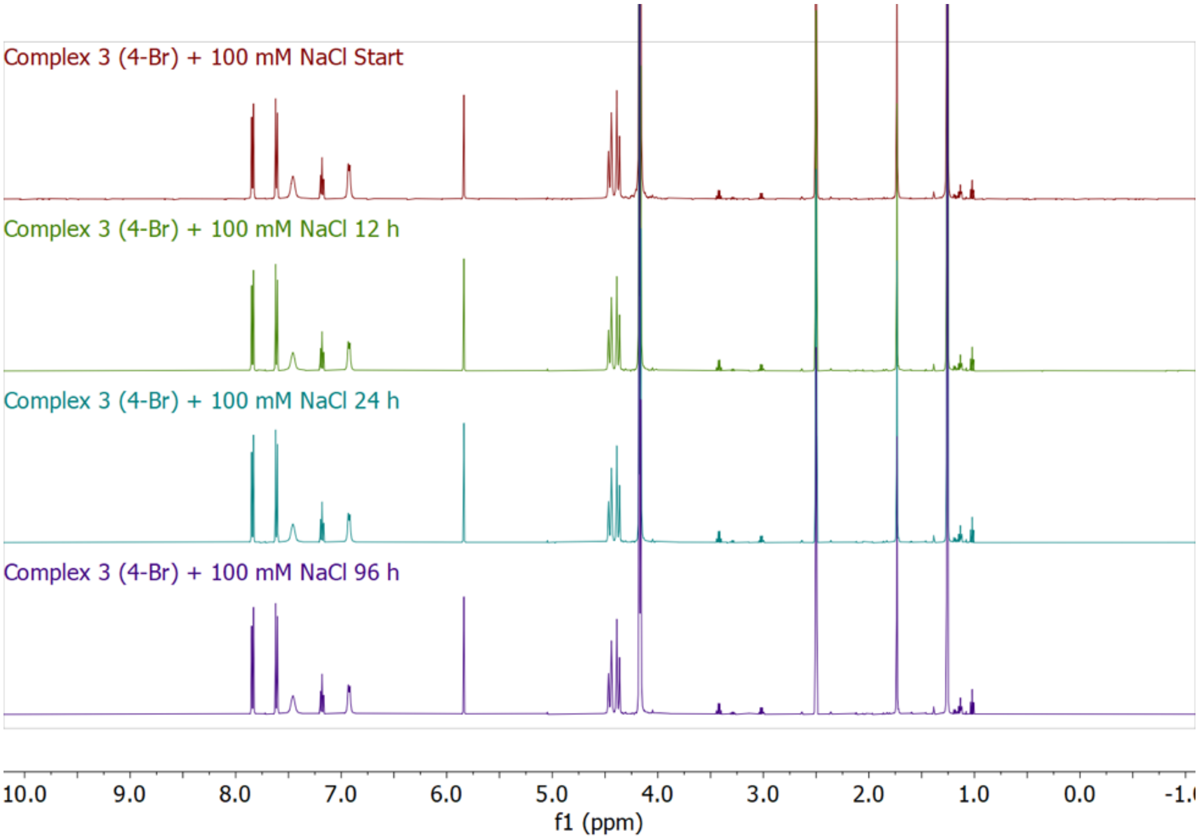


***Figure S 25.*** *$^{1}$H NMR spectra of compound **3** in a 0.1 M solution of NaCl in 70% DMSO-$d_6$ and 30% $D_2O$. Spectra are shown for the start (red), then after 12 h (green), 24 h (blue) and 96 h (purple) (500 MHz, 298 K).*

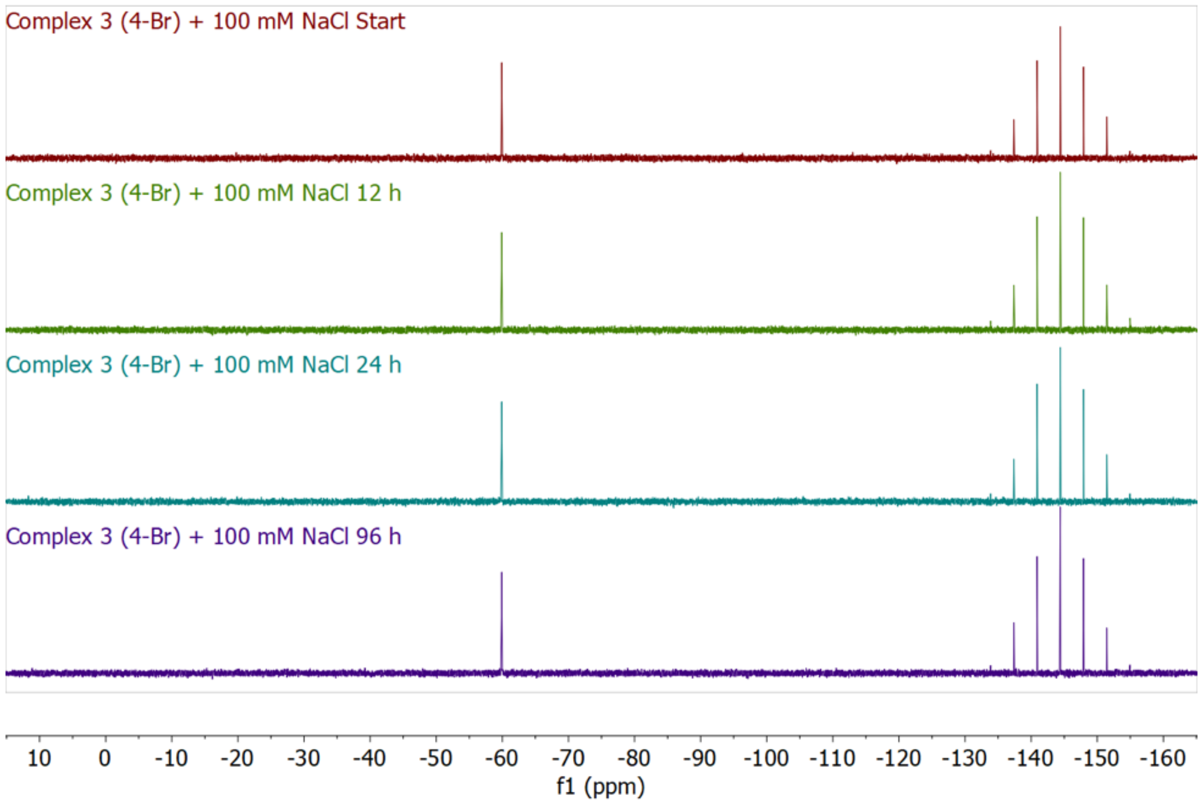


***Figure S 26.*** *$^{31}$P NMR spectra of compound **3** in a 0.1 M solution of NaCl in 70% DMSO-d$_6$ and 30% $D_2O$. Spectra are shown for the start (red), then after 12 h (green), 24 h (blue) and 96 h (purple) (500 MHz, 298 K).*

**Compound 4**

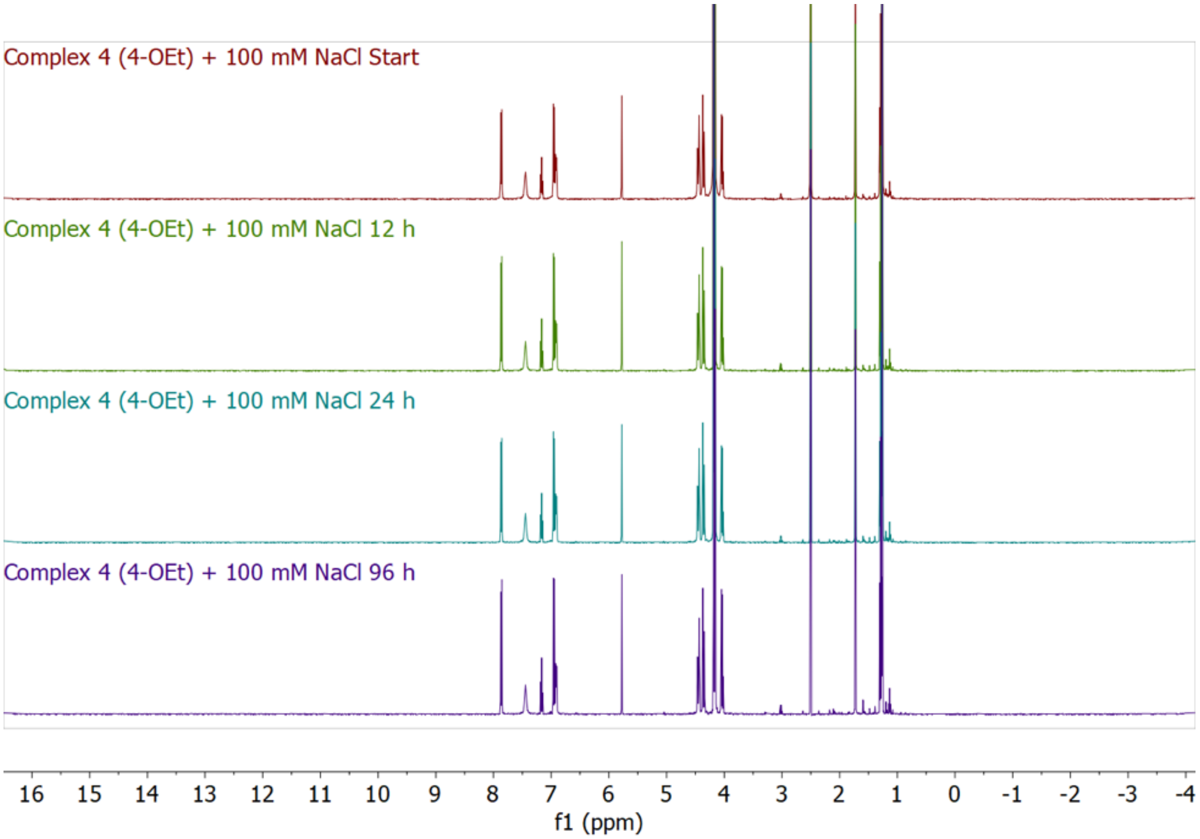


***Figure S 27.*** *$^{1}$H NMR spectra of compound **4** in a 0.1 M solution of NaCl in 70% DMSO-$d_6$ and 30% $D_2O$. Spectra are shown for the start (red), then after 12 h (green), 24 h (blue) and 96 h (purple) (500 MHz, 298 K).*

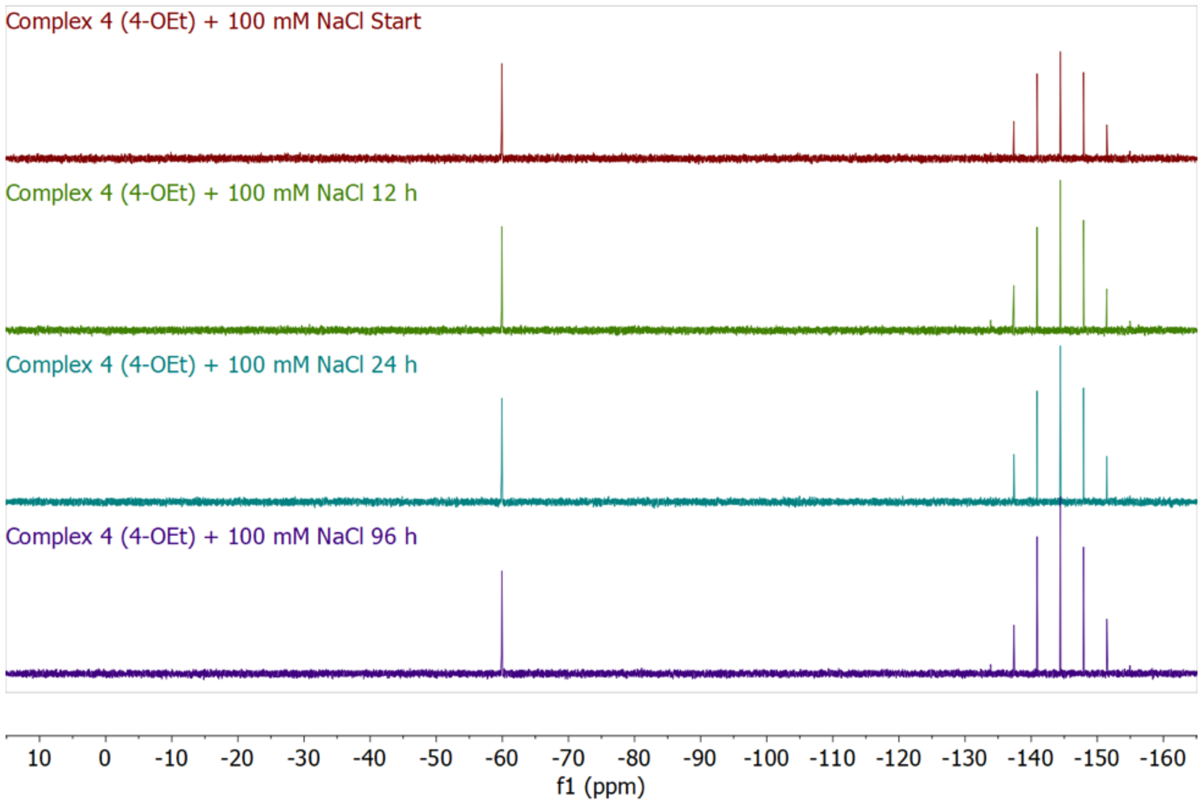


***Figure S 28.*** *$^{31}$P NMR spectra of compound **4** in a 0.1 M solution of NaCl in 70% DMSO-$d_6$ and 30% $D_2O$. Spectra are shown for the start (red), then after 12 h (green), 24 h (blue) and 96 h (purple) (500 MHz, 298 K).*

## Stability of Compounds 1-4 in Supplemented Media over 96 hours

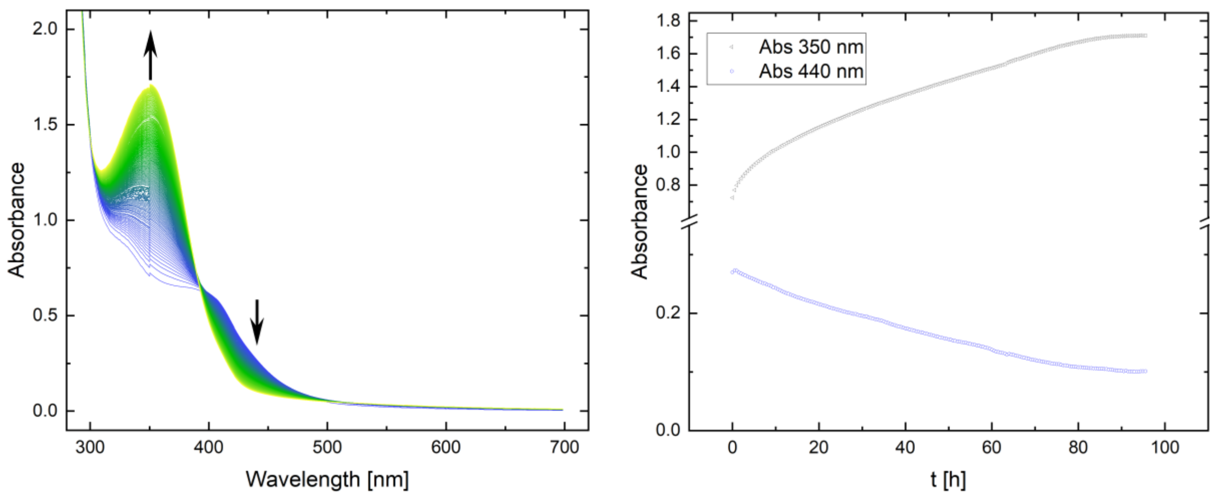


***Figure S 29.*** *(left) UV/vis spectra of compound **1** over 96 h (blue t = 0, yellow t = 96 h) and (right) plot of changes in absorbance at 350 nm and 440 nm, when monitored over 96 h in fully supplemented DMEM phenol-red free media at 37 °C.*

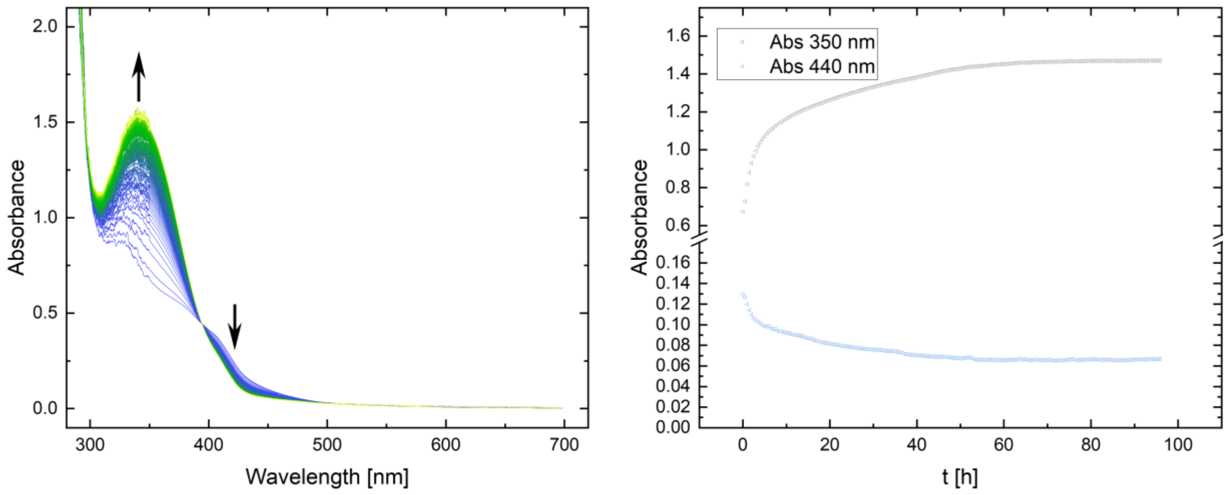


***Figure S 30.*** *(left) UV/vis spectra of compound **2** over 96 h (blue t = 0, yellow t = 96 h) and (right) plot of changes in absorbance at 350 nm and 440 nm, when monitored over 96 h in fully supplemented DMEM phenol-red free media at 37 °C.*

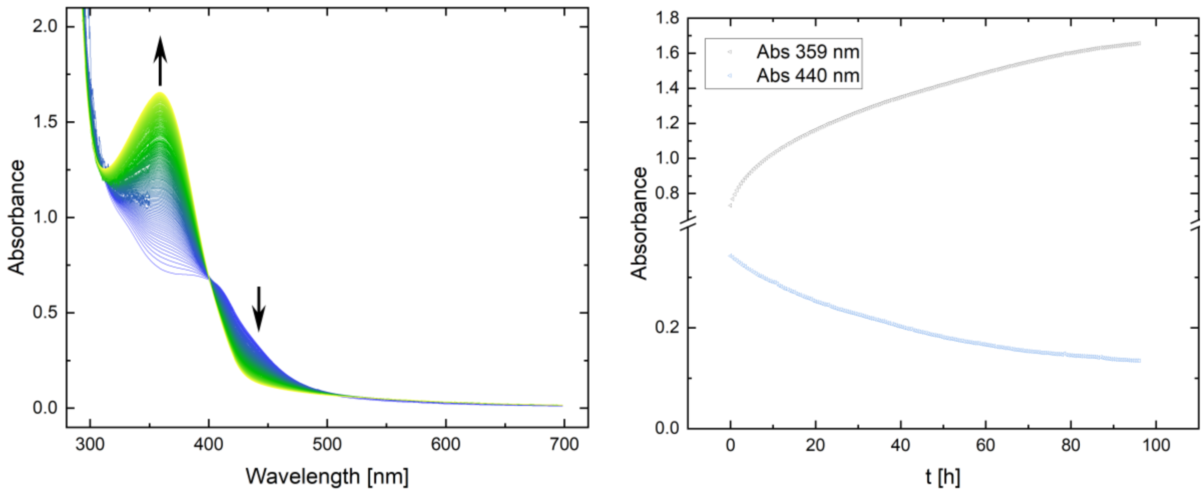


***Figure S 31.*** *(left) UV/vis spectra of compound **3** over 96 h (blue t = 0, yellow t = 96 h) and (right) plot of changes in absorbance at 359 nm (peak maximum) and 440 nm, when monitored over 96 h in fully supplemented DMEM phenol-red free media at 37 °C.*

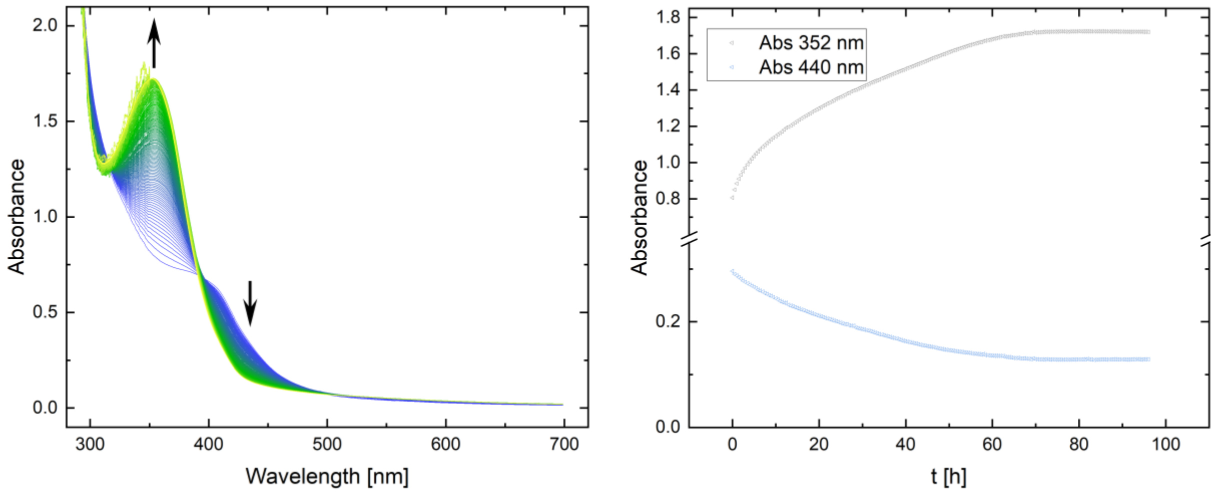


***Figure S 32.*** *(left) UV/vis spectra of compound **4** over 96 h (blue t = 0, yellow t = 96 h) and (right) plot of changes in absorbance at 352 nm (peak maximum) and 440 nm, when monitored over 96 h in fully supplemented DMEM phenol-red free media at 37 °C.*

## High Resolution Mass Spectrometry for Compounds 1-4

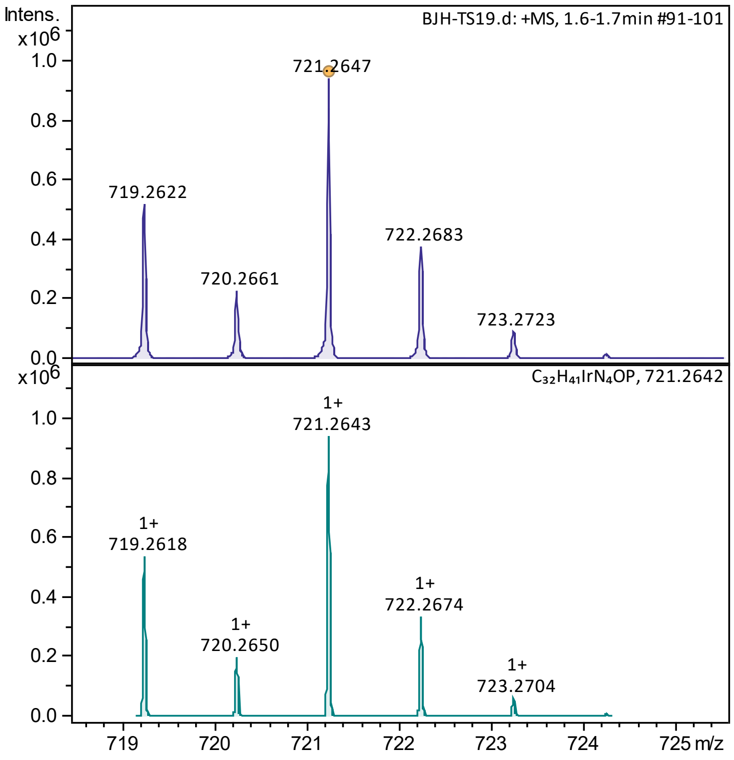


***Figure S 33.*** *Simulated (bottom) and experimental (top) high-resolution mass spectra of compound* ***1****-PF*$_6$*.*

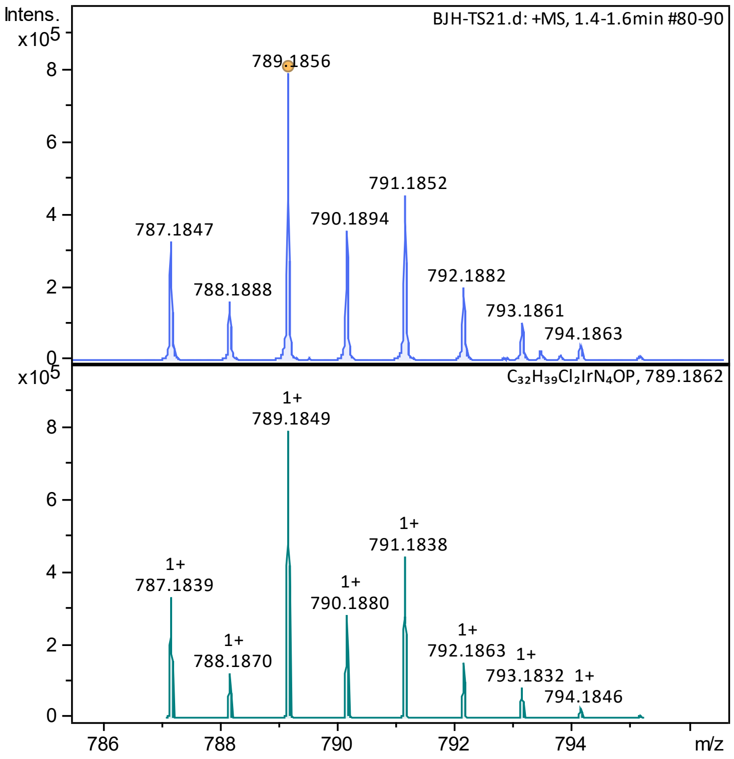


***Figure S 34.*** *Simulated (bottom) and experimental (top) high-resolution mass spectra of compound* ***2****-PF*$_6$*.*

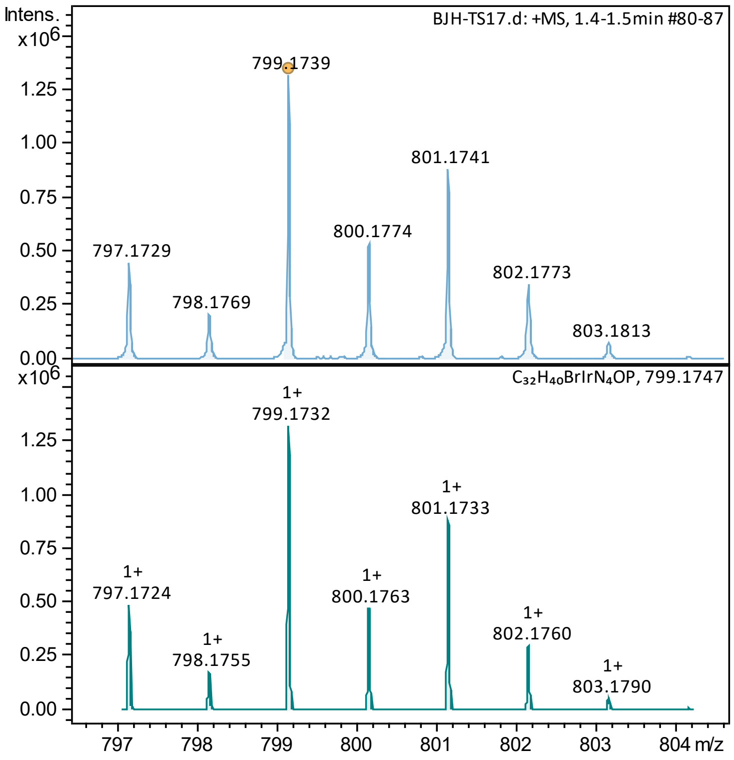


***Figure S 35.*** *Simulated (bottom) and experimental (top) high-resolution mass spectra of compound* ***3****-PF$_6$.*

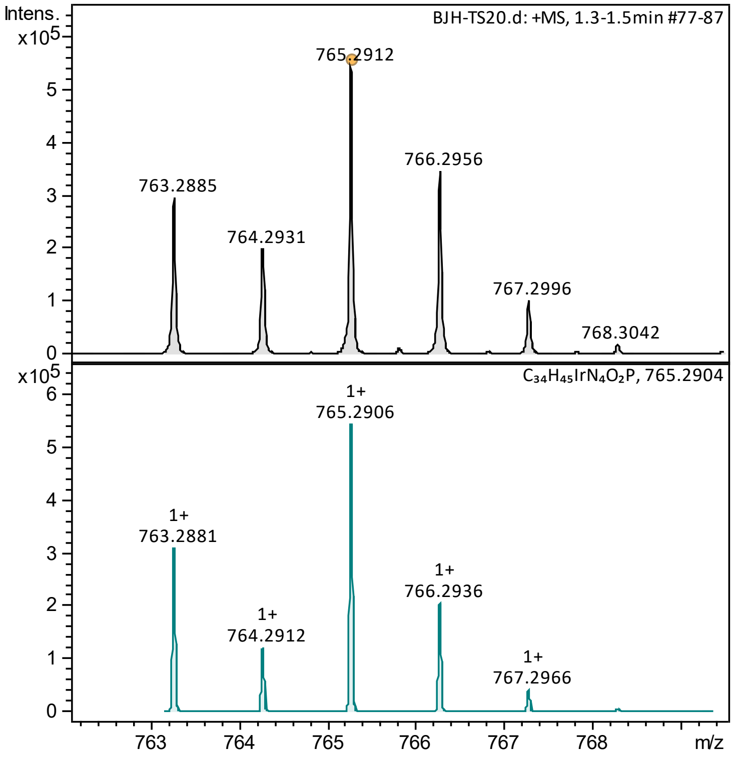


***Figure S 36.*** *Simulated (bottom) and experimental (top) high-resolution mass spectra of compound* ***4****-PF$_6$.*

## High Resolution Mass Spectrometry for Compounds 1-4 after 96 hours in DMSO

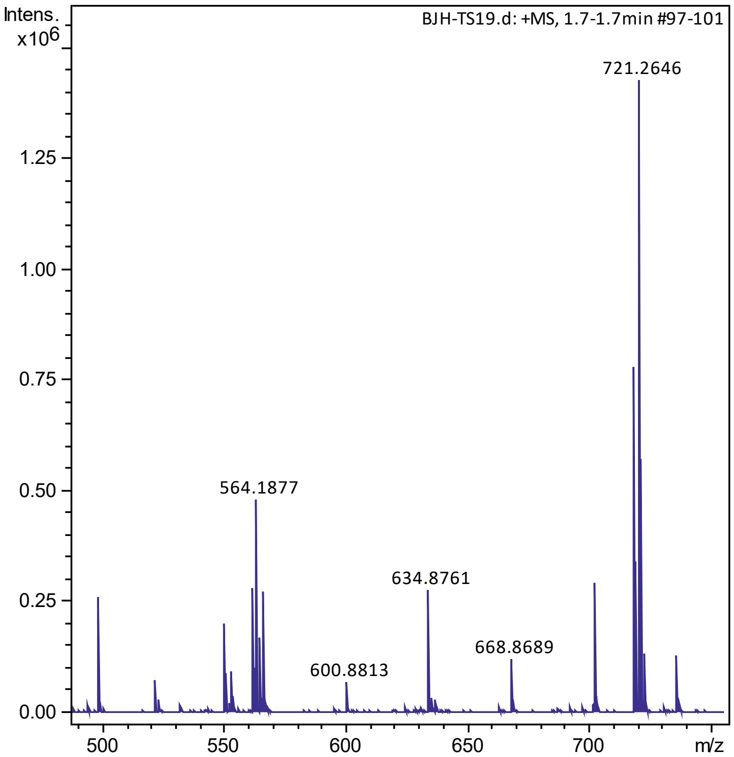


***Figure S 37.*** *HR-MS of compound **1** after incubation for 96 h in DMSO-$d_6$; m/z 721.2646 is observed for [M-$PF_6$] and the DMSO-$d_6$ adduct after PTA dissociation would be observed at* m/z = *648.24.*

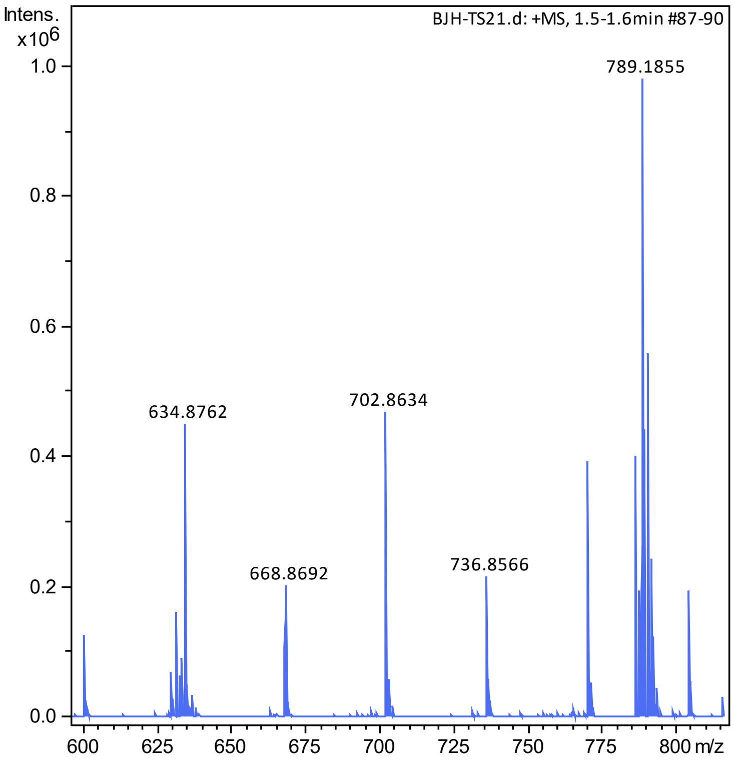


***Figure S 38.*** *HR-MS of compound* ***2*** *after incubation for 96 hours in DMSO-$d_6$; m/z 789.1855 is observed for $[M\text{-}PF_6]^+$ and the DMSO-$d_6$ adduct after PTA dissociation would be observed at m/z 716.16.*

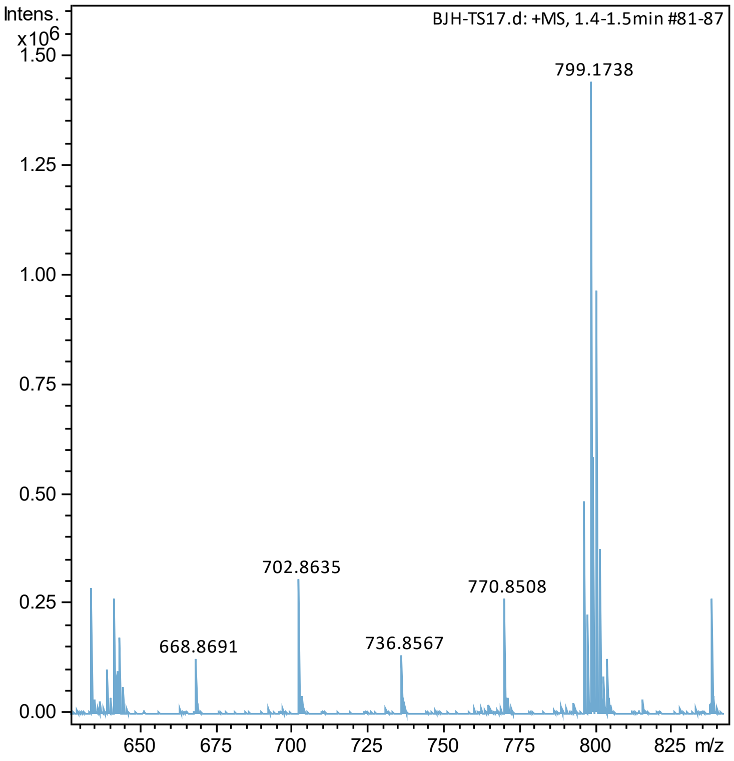


***Figure S 39.*** *HR-MS of compound* ***3*** *after incubation for 96 hours in DMSO-$d_6$; m/z 799.1738 is observed for $[M\text{-}PF_6]$ and the DMSO-$d_6$ adduct after PTA dissociation would be observed at m/z 726.15.*

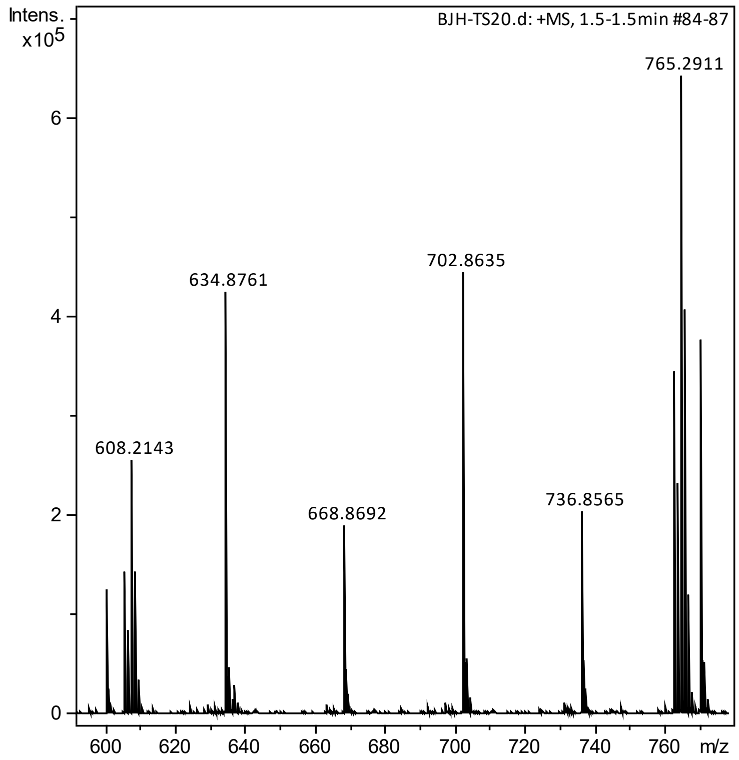


***Figure S 40.*** *HR-MS of compound **4** after incubation for 96 hours in DMSO-d$_6$; m/z 799.1738 is observed for [M-PF$_6$] and the DMSO-d$_6$ adduct after PTA dissociation would be observed at m/z 692.27.*

## Single Crystal X-ray Diffraction

***Table S 1.*** *scXRD data for compounds **1-4** with s.u.s shown in parentheses*

| Compound | 1 | 2 | 3 | 4 |
|---|---|---|---|---|
| CCDC | 2392862 | 2392865 | 2392864 | 2392863 |
| Empirical formula | $C_{32}H_{41}F_6IrN_4OP_2$ | $C_{32}H_{39}Cl_2F_6IrN_4OP_2$ | $C_{32}H_{40}BrF_6IrN_4OP_2$ | $C_{34}H_{45}F_6IrN_4O_2P_2$ |
| Formula weight | 865.83 | 934.71 | 944.73 | 909.88 |
| Temperature/K | 99.99(10) | 100.00(10) | 100.2(7) | 100.00(10) |
| Crystal system | monoclinic | monoclinic | monoclinic | triclinic |
| Space group | $P2_1$/c | $P2_1$/n | $P2_1$/n | $P$-1 |
| a/Å | 10.0797(8) | 9.12360(14) | 11.4398(3) | 15.6128(2) |
| b/Å | 12.2269(8) | 19.9657(3) | 20.5719(5) | 17.3600(2) |
| c/Å | 26.6118(19) | 19.2148(3) | 14.5640(4) | 20.8319(3) |
| α/° | 90 | 90 | 90 | 80.1640(10) |
| β/° | 90 | 90.5277(13) | 94.098(2) | 78.8380(10) |
| γ/° | 90 | 90 | 90 | 82.7470(10) |
| Volume/Å$^3$ | 3279.7(4) | 3499.99(9) | 3418.71(15) | 5432.59(12) |
| Z | 4 | 4 | 4 | 6 |
| $\rho_{calc}$g/cm$^3$ | 1.753 | 1.774 | 1.835 | 1.669 |
| μ/mm$^{-1}$ | 4.236 | 4.124 | 5.234 | 3.843 |
| F(000) | 1720.0 | 1848.0 | 1856.0 | 2724.0 |
| Crystal size/mm$^3$ | 0.409 × 0.168 × 0.132 | 0.237 × 0.190 × 0.166 | 0.313 × 0.198 × 0.108 | 0.277 × 0.143 × 0.143 |
| Radiation | Mo Kα (λ = 0.71073) | Mo Kα (λ = 0.71073) | Mo Kα (λ = 0.71073) | Mo Kα (λ = 0.71073) |
| 2Θ range/° | 4.524 to 62.012 | 4.598 to 62 | 4.082 to 61.8 | 4.208 to 61.938 |
| Index ranges | -10 ≤ h ≤ 12, -15 ≤ k ≤ 14, -37 ≤ l ≤ 29 | -12 ≤ h ≤ 12, -28 ≤ k ≤ 27, -25 ≤ l ≤ 27 | -16 ≤ h ≤ 15, -28 ≤ k ≤ 29, -18 ≤ l ≤ 20 | -21 ≤ h ≤ 22, -23 ≤ k ≤ 23, -28 ≤ l ≤ 25 |
| Reflections collected | 16638 | 53652 | 29645 | 90844 |
| Independent reflections | 7019 [$R_{int}$ = 0.0479, $R_{sigma}$ = 0.0613] | 9519 [$R_{int}$ = 0.0415, $R_{sigma}$ = 0.0322] | 8785 [$R_{int}$ = 0.0540, $R_{sigma}$ = 0.0573] | 27296 [$R_{int}$ = 0.0686, $R_{sigma}$ = 0.0745] |
| Data/restraints/ parameters | 7019/0/421 | 9519/0/439 | 8785/0/470 | 27296/0/1359 |
| GOOF on F$^2$ | 1.037 | 1.027 | 1.042 | 1.052 |
| Final R indexes [I>=2σ (I)] | $R_1$ = 0.0396, $wR_2$ = 0.0854 | $R_1$ = 0.0282, $wR_2$ = 0.0609 | $R_1$ = 0.0333, $wR_2$ = 0.0712 | $R_1$ = 0.0602, $wR_2$ = 0.1541 |
| Final R indexes [all data] | $R_1$ = 0.0589, $wR_2$ = 0.0912 | $R_1$ = 0.0372, $wR_2$ = 0.0632 | $R_1$ = 0.0450, $wR_2$ = 0.0744 | $R_1$ = 0.0832, $wR_2$ = 0.1658 |
| Largest diff. peak/hole / e Å$^{-3}$ | 1.44/-1.08 | 1.98/-0.65 | 2.40/-1.89 | 6.95/-2.32 |

***Table S 2.*** *Bond angles (°) of complexes **1-4** with s.u.s in parentheses.*

| Bond Angles | 1 | 2 | 3 | 4 |
|---|---|---|---|---|

| | | | | |
|---|---|---|---|---|
| O1-Ir1-P1 | 89.19(10) | 84.40(5) | 87.83(7) | 86.32(14)/ 83.22(13)/ 86.29(15) |
| O1-Ir1-N1 | 87.72(15) | 88.84(8) | 87.74(10) | 88.4(2)/ 88.35(19)/ 88.9(2) |
| N1-Ir1-P1 | 86.06(11) | 88.76(6) | 86.29(8) | 88.94(16)/ 90.37(15)/ 88.35(17) |
| O1-C1-C2 | 123.7(5) | 128.1(2) | 124.8(3) | 125.2(6)/125.6(6)/ 126.0(7) |
| C1-C2-C3 | 127.1(5) | 127.3(3) | 126.8(3) | 128.4(6)/ 128.9(7)/ 125.8(7) |
| N1-C3-C2 | 125.8(5) | 124.3(2) | 124.6(3) | 124.1(6)/ 124.1(7)/ 126.2(6) |

**Chemosensitivity Studies**

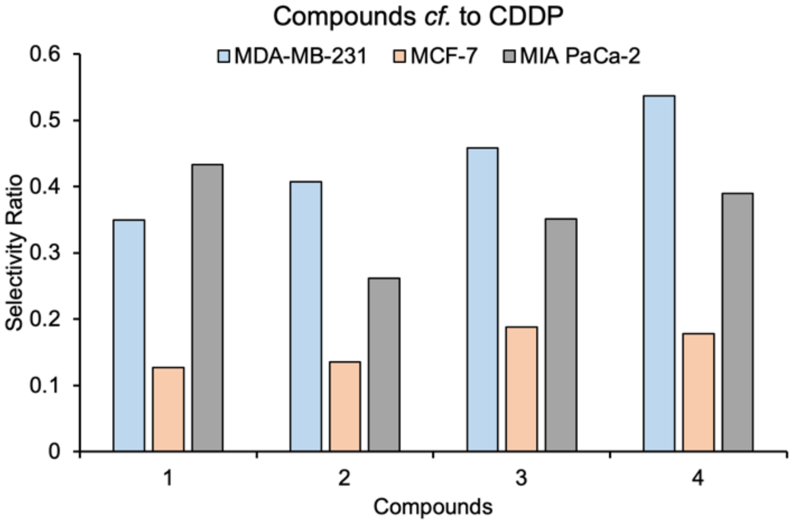


***Figure S 41.*** *Selectivity ratios (SR) when comparing the $IC_{50}$ values of compounds **1-4** with the $IC_{50}$ values of CDDP. SR > 1 indicates a selectivity for the compounds **1-4** and SR <1 indicates a selectivity for CDDP.*

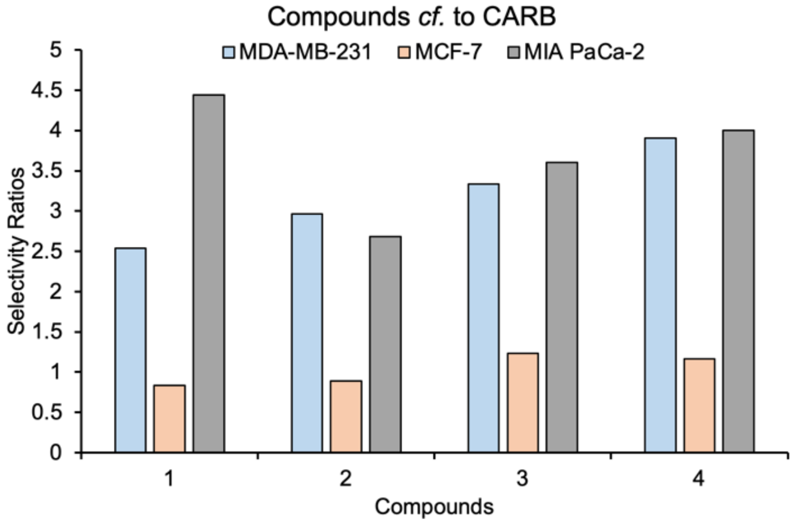


***Figure S 42.*** *Selectivity ratios (SR) when comparing the* $IC_{50}$ *values of compounds* ***1-4*** *with the* $IC_{50}$ *values of CARB. SR > 1 indicates a selectivity for the compounds* ***1-4*** *and SR <1 indicates a selectivity for CARB.*

## LogP Predictions

The logP values were predicted using SwissADME (http://www.swissadme.ch).[8] All compounds follow the order 2',4'-diCl > 4'-Br > 4'-OEt > H and whilst there are no direct trends between the predicted logP values and the cytotoxicity values, the most active Ir(III)-PTA complex (**1**) is the least lipophilic of the library, and this lipophilicity increases by changing from a neutral chlorido complex (1-Cl to 4-Cl) to a charged PTA complex. These logP values are significantly higher in comparison to experimental values, where the logP of cisplatin is reported as -2.19.[9]

***Table S 3.*** *Predicted logP values of ligands* ***L1-L4****, previously reported Ir(III)-Cl complexes* ***1-Cl - 4-Cl*** *and the new Ir(III)-PTA complexes* ***1-4****.*

| Compound | Formula | Mol Wt (g/mol) | Average of 5 logP prediction tools |
|---|---|---|---|
| **L1** | $C_{16}H_{15}NO$ | 237.30 | 3.43 |
| **L2** | $C_{16}H_{13}Cl_2NO$ | 306.19 | 4.39 |
| **L3** | $C_{16}H_{14}BrNO$ | 316.20 | 3.98 |
| **L4** | $C_{18}H_{19}NO_2$ | 281.35 | 3.67 |
| **1-Cl** | $C_{26}H_{29}ClIrNO$ | 599.19 | 5.02 |
| **2-Cl** | $C_{26}H_{27}Cl_3IrNO$ | 668.08 | 5.97 |
| **3-Cl** | $C_{26}H_{28}BrClIrNO$ | 678.09 | 5.56 |
| **4-Cl** | $C_{28}H_{33}ClIrNO_2$ | 643.24 | 5.21 |
| **1** | $C_{32}H_{41}IrN_4OP_2F_6$ | 865.86 | 5.80 |
| **2** | $C_{32}H_{39}Cl_2IrN_4OP_2F_6$ | 934.75 | 6.75 |
| **3** | $C_{32}H_{40}BrIrN_4OP_2F_6$ | 944.76 | 6.33 |
| **4** | $C_{34}H_{45}IrN_4O_2P_2F_6$ | 909.91 | 6.00 |
| **CDDP** | $Cl_2H_6N_2Pt$ | 300.05 | 0.99 |
| **CARB** | $C_6H_{12}N_2O_4Pt$ | 371.26 | 0.37 |

## Results against the MCF-7 cell line

### DNA Morphology with Compound 1

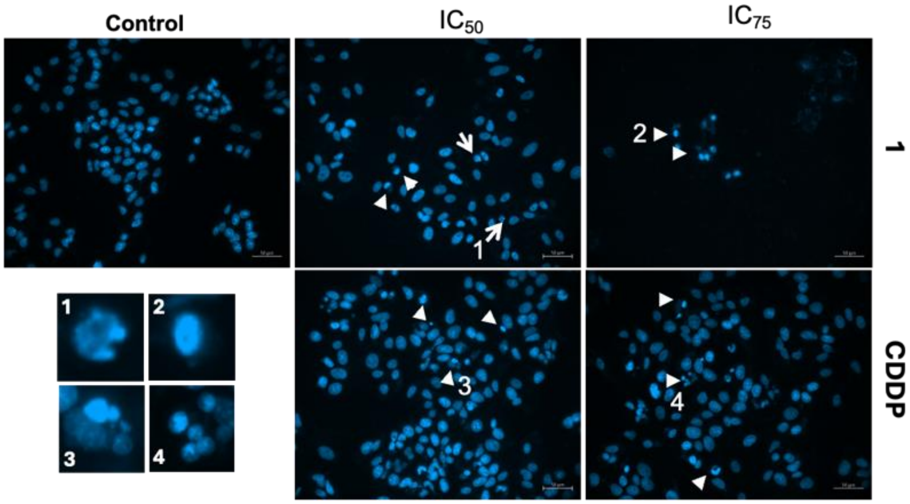


***Figure S 43.*** *DAPI staining of MCF-7 cells after 48 h treatment with compound* ***1*** *or CDDP. Arrowheads mark condensed nuclei, and arrows indicate fragmented nuclei. Insets show representative morphologies: condensed nuclei (1, 2) and fragmented nuclei forming apoptotic bodies (3, 4). Images were acquired with a Leica DMI8 fluorescence microscope at 40× magnification. Scale bar = 50 µm.*

### Induction of Apoptosis with Compound 1

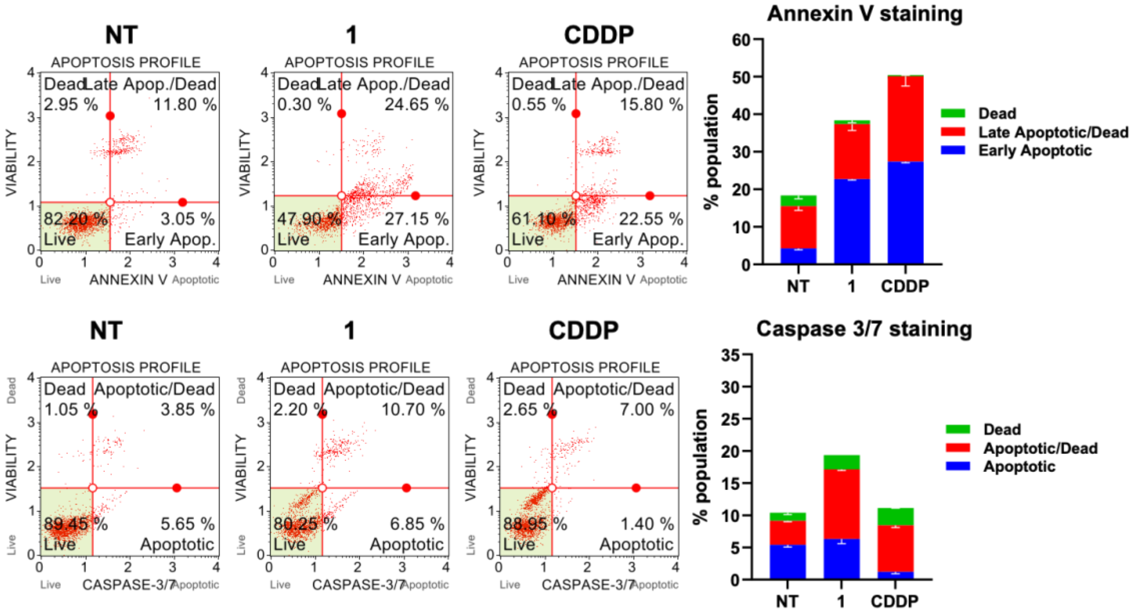


*Figure S 44. Apoptosis induction following treatment with compound **1** and CDDP: (A) Annexin V / 7-AAD positivity and (B) Caspase 3/7 activity was assessed in MCF-7 cells through flow cytometric analysis. Cells were treated with compound 1 or CDDP at half-maximal inhibitory concentrations for 72 h and analyzed using the Muse Cell Analyzer.*

### Quantification of PARP with Compound 1

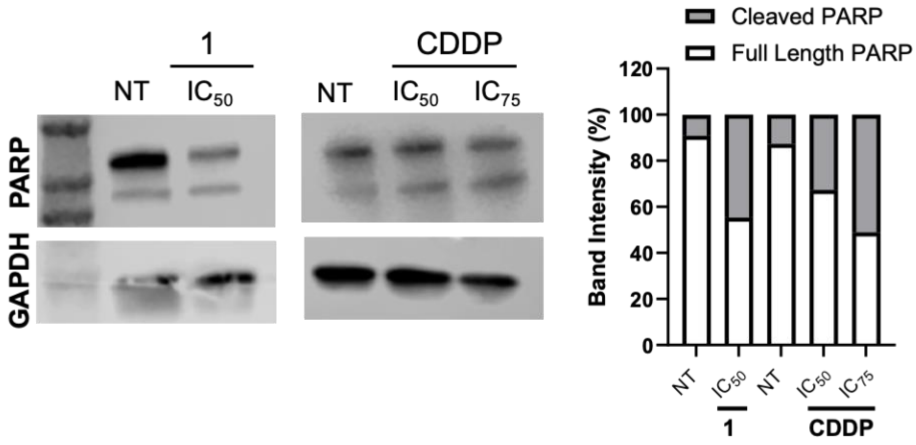


*Figure S 45. Quantification of PARP cleavage as an indicator of apoptosis: MCF-7 cells were treated with $IC_{50}$ and $IC_{75}$ doses of compound **1** or CDDP as a positive control for 48 h. The left panel displays Western blot images of cleaved and full-length PARP, while the graph shows the quantification of band intensities.*

## References


(1) Cosier, B. J.; Glazer, A. M. A Nitrogen-Gas-Stream Cryostat for General X-Ray Diffraction Studies. *J Appl Crystallogr* **1986**, *19* (2), 105–107. https://doi.org/10.1107/S0021889886089835.

(2) CrystAlis PRO. CrysAlis PRO. Agilent Technologies Ltd: Yarnton, Oxfordshire, England. 2014.

(3) Sheldrick, G. M. Foundations and Advances SHELXT-Integrated Space-Group and Crystal-Structure Determination. *Acta Cryst* **2015**, *71*, 3–8. https://doi.org/10.1107/S2053273314026370.

(4) Dolomanov, O. V.; Bourhis, L. J.; Gildea, R. J.; Howard, J. A. K.; Puschmann, H. OLEX2: A Complete Structure Solution, Refinement and Analysis Program. *urn:issn:0021-8898* **2009**, *42* (2), 339–341. https://doi.org/10.1107/S0021889808042726.

(5) Lucas, S. J.; Lord, R. M.; Wilson, R. L.; Phillips, R. M.; Sridharan, V.; McGowan, P. C. Synthesis of Iridium and Ruthenium Complexes with (N,N), (N,O) and (O,O) Coordinating Bidentate Ligands as Potential Anti-Cancer Agents. *Dalton Trans* **2012**, *41* (45), 13800–13802. https://doi.org/10.1039/C2DT32104A.

(6) Lord, R. M.; Hebden, A. J.; Pask, C. M.; Henderson, I. R.; Allison, S. J.; Shepherd, S. L.; Phillips, R. M.; McGowan, P. C. Hypoxia-Sensitive Metal β-Ketoiminato Complexes Showing Induced Single-Strand DNA Breaks and Cancer Cell Death by Apoptosis. *J Med Chem* **2015**, *58* (12), 4940–4953. https://doi.org/10.1021/acs.jmedchem.5b00455.

(7) Lord, R. M.; Zegke, M.; Henderson, I. R.; Pask, C. M.; Shepherd, H. J.; McGowan, P. C. β-Ketoiminato Iridium(III) Organometallic Complexes: Selective Cytotoxicity towards Colorectal Cancer Cells HCT116 P53-/-. *Chem Eur J* **2019**, *25* (2), 495–500. https://doi.org/10.1002/CHEM.201804901.

(8) *SwissADME*. http://www.swissadme.ch/ (accessed 2025-07-16).

(9) Chen, T. Y.; Tsai, M. J.; Chang, L. C.; Wu, P. C. Co-Delivery of Cisplatin and Gemcitabine via Viscous Nanoemulsion for Potential Synergistic Intravesical Chemotherapy. *Pharmaceutics 2020, Vol. 12, Page 949* **2020**, *12* (10), 949. https://doi.org/10.3390/PHARMACEUTICS12100949.
